# Effectiveness of interventions for improving social inclusion outcomes for people with disabilities in low‐ and middle‐income countries: A systematic review

**DOI:** 10.1002/cl2.1316

**Published:** 2023-03-21

**Authors:** Ashrita Saran, Xanthe Hunt, Howard White, Hannah Kuper

**Affiliations:** ^1^ Campbell South Asia Delhi India; ^2^ Institute for Life Course Health Research, Department of Global Health Stellenbosch University Cape Town South Africa; ^3^ International Centre for Evidence on Disability London School of Hygiene & Tropical Medicine London UK

## Abstract

**Background:**

People with disabilities—more than a billion people worldwide—are frequently excluded from social and political life, and often experience stigmatising attitudes and behaviours from people without disabilities. This stigma, coupled with inaccessible environments and systems and institutional barriers (e.g., lack of inclusive legislation), may result in discrimination against people with disabilities (and their families) to the degree that they are not able to enjoy their rights on an equal basis with others.

**Objectives:**

This review examines the effectiveness of interventions for improving social inclusion outcomes (acquisition of skills for social inclusion, broad‐based social inclusion, and improved relationships) for people with disabilities in low‐ and middle‐income countries (LMICs).

**Search Methods:**

We searched academic and online databases, carried out citation tracking of included studies, and contacted experts to ensure our search was as comprehensive as possible. We also ran the searches with search terms specific to social inclusion review using Open Alex in EPPI reviewer.

**Selection Criteria:**

We included all studies which reported on impact evaluations of interventions to improve social inclusion outcomes for people with disabilities in LMIC.

**Data Collection and Analysis:**

We used review management software EPPI Reviewer to screen the search results. Two review authors independently extracted the data from each study report, including for the confidence in study findings appraisal. Data and information were extracted regarding available characteristics of participants, intervention characteristics and control conditions, research design, sample size, risk of bias and outcomes, and results. Random‐effects inverse variance weighted meta‐analytic methods were used to synthesise standardised mean differences for the outcomes.

**Main Results:**

We identified 37 experimental and quasi‐experimental studies. Studies were conducted in 16 countries, with the majority of the included studies (*n* = 13) from South Asia and nine each from East Asia, the Pacific, the Middle East, and North Africa. Most studies targeted children with disabilities (*n* = 23), and 12 targeted adults with disabilities. Most focused on people with intellectual disabilities (*n* = 20) and psychosocial disabilities (*n* = 13). Regarding intervention content, most (*n* = 17) of the included programmes aimed to improve the social and communication skills of people with disabilities through social skills training programmes. Ten studies aimed at providing personal assistance and support and evaluated the effects of a parent training programme on the interactive skills of parents of children and their children with disabilities. We calculated effect sizes from experimental and quasi‐experimental studies for outcomes on skills for social inclusion, relationships of people with disabilities with family and community members, and broad‐based social inclusion among people with disabilities. A meta‐analysis of 16 studies indicates an overall positive, statistically significant and large effect of the interventions for skills for social inclusion with standardised mean difference (SMD) = 0.87, confidence interval (CI) = 0.57 to 1.16, *k* = 26, *I* ^2^ = 77%, *p* < 0.001). For relationships across 12 studies, we find a positive but moderate effect (SMD = 0.61, CI = 0.41 to 0.80, *k* = 15, *I* ^2^ = 64%, *p* < 0.01). As for the overall effect on broad‐based social inclusion, we find the average effect size was large, and there was significant dispersion across studies (SMD = 0.72, CI = 0.33 to 1.11, *k* = 2, *I* ^2^ = 93%, *p* < 0.01). Despite the significant and large effects estimated by the studies, some limitations must be noted. Although there was a consensus on the direction of the effects, the studies presented considerable heterogeneity in the size of the effects. A majority (*n* = 27) of studies were assessed to be of low confidence related to methodological limitations, so the findings must be interpreted with caution. Tests for publication bias show that the effect sizes of social skills (*p* < 0.01) and social inclusion (*p* = 0.01) are all likely to be inflated by the existence of the publication bias.

**Authors’ Conclusions:**

The review's findings suggest that various interventions to improve the social inclusion of people with disabilities have a significant positive effect. Interventions such as social and communication training and personal assistance led to significant improvement in the social behaviour and social skills of people with disabilities. Studies targeting broad‐based social inclusion showed a large and significant positive effect. A moderate effect was reported from interventions designed to improve relationships between people with disabilities and their families and communities. However, the findings of this review must be interpreted cautiously, given the low confidence in study methods, severe heterogeneity and significant publication bias. The available evidence focused primarily on individual‐level barriers such as interventions for improving social or communications skills of people with disabilities and not the systemic drivers of exclusions such as addressing societal barriers to inclusion, such as stigma reduction, and interventions to strengthen legislation, infrastructure, and institutions.

## 1 PLAIN LANGUAGE SUMMARY

### 1.1 Social inclusion interventions in low‐ and middle‐income settings have a meaningful positive effect on people with disabilities

There is promising evidence that interventions can improve the social skills and relationships of people with disabilities in low‐ and middle‐income country (LMIC) settings. However, there is a lack of evidence on what works to improve social inclusion and community participation for this group.

### 1.2 What is this review about?

There are approximately one billion people with disabilities. They are frequently excluded from social and political activities, which is a violation of their fundamental rights. A core reason for the exclusion is that people with disabilities often experience stigmatising attitudes and behaviours from others. Inaccessible environments and systems, and institutional barriers also contribute to discrimination against people with disabilities.

Social inclusion outcomes can be improved through interventions designed to develop skills for social inclusion (e.g., social and communication skill training), broad‐based social inclusion (e.g., enhancing access and participation in sports and the arts) and improved relationships (e.g., social support and violence prevention).

### 1.3 What is the aim of this review?

In this review, we examine the effectiveness of interventions designed to improve social inclusion outcomes for people with disabilities in LMICs.

### 1.4 What studies are included?

We identified a broad range of interventions that reported improvements in social inclusion outcomes for people with disabilities in LMICs. Many of the studies had methodological limitations, which means that the confidence in the study findings was generally low.

We present the findings from 37 studies that evaluated the effectiveness of interventions on social inclusion outcomes for people with disabilities in LMICs. The studies were conducted between 2000 and 2022. Studies were conducted in 16 countries, with 12 in India and 6 in China.

### 1.5 What are the main findings of this review?

The findings of the review suggest that social inclusion interventions have a substantial and positive effect on the social behaviour, social skills, and broad‐based social inclusion of persons with disabilities.

A moderate effect was reported from interventions designed to improve relationships between people with disabilities and their families and communities.

### 1.6 What do the findings of this review mean?

This review highlights promising evidence on the effectiveness of interventions to improve the social inclusion of people with disabilities.

Evidence on interventions for people with disabilities has, however, been primarily focused on interventions at the individual level, such as enhancing social skills and relationships.

There is a gap in evidence on community‐level interventions that address societal barriers to inclusion, such as stigma reduction, and system‐level interventions that improve legislation, infrastructure and institutions.

### 1.7 How up‐to‐date is this review?

The review authors searched for studies up to March 2022.

## 2 BACKGROUND

### 2.1 The problem, condition or issue

Social inclusion is a multi‐faceted construct but most commonly refers to inclusion in life's social, political, cultural, and economic dimensions (Khan et al., 2015). A United Nations (UN) report on the World Social Situation defines social inclusion as the ‘process of improving participation in society, particularly for disadvantaged people, through enhancing opportunities, access to resources, voice and respect for rights (UN, 2016). A key group that often faces disadvantages is people with disabilities, in terms of poverty, negative attitudes and exclusion from society.

Globally, there are one billion people with disabilities, 80% of whom live in low‐ and middle‐income countries (LMICs) (WHO, 2011). People with disabilities are among the most marginalised in society. In addition to experiencing stigmatising attitudes and behaviours from people without disabilities, family members and society at large, they often experience self‐stigma (e.g., a feeling of worthlessness) (Bond Disability and Development Group, 2017). Negative and inaccurate perceptions and beliefs can be widespread in society, often leading to exclusion, exploitation, abuse and violence (WHO, 2010) (Jones et al., 2012) and feelings of shame (Bond Disability and Development Group, 2017). It is common for the families and caregivers of those with disabilities to be stigmatised or discriminated against (DFID, 2018). These stigmatising attitudes, coupled with inaccessible environments and systems and institutional barriers (e.g., lack of inclusive legislation), may result in discrimination against people with disabilities and potentially their families so that they are not able to enjoy their rights on an equal basis with others. Disability discrimination means any disparity, exclusion, or restriction that prevents people with disabilities from accessing their rights (MacKay, 2006; UN, 2016), and it is widespread (Mactaggart et al., 2016). According to WHO/UNFPA, prejudicial attitudes and misconceptions contribute to discrimination against people with disabilities by denying them opportunities, such as setting up relationships, expressing their sexuality, getting married, and raising a family (WHO/UNFPA, 2009). A person with a disability may experience discrimination across all aspects of their lives, including political, economic, social, cultural, civil, or any other field. Therefore, stigmatising attitudes and discrimination against people with disabilities manifest as a lack of social inclusion.

These exclusions result in lower participation of people with disabilities in education, economics and policies compared to others in the population (WHO, 2010). Consequently, people with disabilities tend to have a lower level of educational attainment, poorer health, fewer economic opportunities, and increased poverty risk (Banks et al., 2017; Bright & Kuper, 2018). As a result of social exclusion, people with disabilities encounter various challenges in accessing services that others have long taken for granted, including healthcare, education, employment, and transportation (UN, 2018). These difficulties are exacerbated in less advantaged communities and increase the risk of social exclusion and poverty (WHO, 2010). These exclusions contradict the UN Convention on the Rights of Persons with Disabilities (UNCRPD), which supports the fulfilment of rights for persons with disabilities across diverse areas, including education, employment, and social participation.

Social exclusion impacts people with disabilities differently depending on their impairment type, gender, socioeconomic and cultural background, and other characteristics and contexts (WHO, 2010). For example, older people with disabilities are often discriminated against because of their age and disability, and older women may be particularly disenfranchised (UN Women, 2020). People with certain impairment types may face exceptionally high levels of discrimination. For instance, people with albinism are often targeted in many parts of the world due to deep‐rooted discriminatory beliefs, such as that their body parts can bring good fortune (Nebre, 2018). Societal stigma can result in people with psychosocial and intellectual disabilities being segregated, constrained in their homes, or institutionalised (Scior et al., 2015; p.6).

Social inclusion of people with disabilities is recognised as a fundamental right in the UNCRPD, including in ‘participation in cultural life, recreation, leisure, and sport’ (Article 30) and ‘participation in political and public life (Article 29). Furthermore, other rights, for example, the right to education (Article 24), may not be realised without social inclusion. The Sustainability Development Goals (SDGs) are also relevant to this issue (UN, 2016), including SDG4 ‘Guaranteeing equal and accessible education by building inclusive learning environments and providing the needed assistance for persons with disabilities’, and SDG 8 ‘Promote sustained, inclusive and sustainable economic growth, full and productive employment and decent work for all’, SDG 10 ‘Emphasising the social, economic and political inclusion of persons with disabilities’ *and* SDG 11 ‘Creating accessible cities and water resources, affordable, accessible and sustainable transport systems, providing universal access to safe, inclusive, accessible and green public spaces’. The SDGs may not be achieved if people with disabilities are excluded from equal participation in all aspects of life.

In addition to the value of the contributions people with disabilities make to society, there are costs associated with exclusion and gains associated with inclusion (Banks; Polack, 2014). Moreover, meaningful inclusion of people with disabilities, such as in the arts, sports, and community processes, can challenge stigmatising attitudes and norms and, in turn, reduce discrimination and social exclusion (Lundberg et al., 2011). In addition to enhancing health, well‐being, self‐esteem, dignity, and social connections, social inclusion of individuals with disabilities promotes economic opportunities and social connections in many ways (World Bank, 2020). The importance of inclusion in education cannot be overstated, as education is essential for skill development. Schools are crucial for developing social networks, peer relationships, friendships and influential linkages that may further lead to job opportunities or promote entrepreneurship (Hanushek & Wößmann, 2007). Similarly, employment facilitates social participation and improves human dignity and social cohesion. Providing education and livelihood inclusion for children with disabilities can also facilitate the achievement of other rights; for instance, schools and workplaces function as critical healthcare providers, including distributing food and drugs at school and accessing social assistance (UN, 2018).

Despite the benefits of social inclusion, WHO (2010) reports that people with disabilities suffer from widespread social exclusion, stigma, and discrimination in LMICs. For instance, studies conducted in India, Cameroon, and Guatemala show that adults with disabilities face more significant participation restrictions in interpersonal relationships and social, community, and civic with disabilities and a lack of opportunity for engagement in activities outside the home (Pinilla‐Roncancio et al., 2020). A study conducted in refugee camps in Tanzania and conflict‐affected Ukraine found that older people have a high degree of social isolation in all aspects of their lives—political, economic, social, cultural, civil, and other—including denial of reasonable accommodation (Sheppard et al., 2018). It is therefore essential to develop and implement interventions that overcome the barriers limiting the social inclusion of people with disabilities, including physical barriers (e.g., inaccessible transport and buildings, community centres and sports facilities), as well as informational barriers (e.g., lack of sign‐language interpreters at cultural events).

### 2.2 Description of the intervention

#### 2.2.1 The intervention

This review examines a broad range of interventions that may improve the social inclusion of people with disabilities. Social inclusion is considered per WHO's Community‐Based Rehabilitation Guidelines (CBR) (WHO, 2010). The WHO has endorsed the concept of CBR as a way to improve the lives of persons with disabilities. One of the five pillars of CBR is ‘social’ (WHO, 2010). To classify interventions, we used five components of the ‘social’ pillar of the CBR matrix: personal assistance, relationship, marriage and family, culture and arts, recreation, leisure and sports, and justice. Table 1 lists specific interventions for each category (e.g., formal assistance and support, informal assistance and support). Therefore, the CBR will serve as a guiding framework for the intervention categories, as listed below, to realise the full inclusion and empowerment of persons with disabilities.

**Table 1:** Intervention and sub‐intervention categories.

Intervention category	Intervention sub‐category	Description
Personal assistance	Formal personal assistance and support (including trainings)	Governmental, non‐governmental organisations and the private sector offer a formal assistance programme. Sometimes, personal assistance can be funded by disability pensions, guardianship awards, or caregiver allowances (Khasnabis, 2010).
	Informal personal assistance and support (including training)	Assistance from family members, friends, neighbours and/or volunteers (Khasnabis, 2010).
Relationship, marriage and family	Networking and social support	Providing people with disabilities with social support and networking opportunities includes linking them with support networks available in their community, such as disabled people's organisations (DPOs) and self‐help groups (Khasnabis, 2010).
	Improving community attitude	Efforts to promote positive images and role models of people with disabilities (e.g., through the media); and information on services available (Khasnabis, 2010).
	Community living	Supporting people with disabilities to access their preferred living arrangements and helping people with disabilities who are homeless to find appropriate accommodation, preferably in the community.
	Social and communication skill training	Training may focus on verbal and nonverbal behaviours common in social relationships, or on improving communication skills.
	Violence prevention interventions	Interventions to prevent violence such as raising awareness, establishing links to local stakeholders for support, access to health care services, etc. (Khasnabis, 2010).
Culture and arts	Access and participation in cultural programme, arts, drama and theatres	Provision of cultural materials, television programmes, films, theatre and other cultural activities, in accessible formats; accessibility of cultural performances or services, including theatres, museums, cinemas, libraries, tourism services, monuments and sites of national cultural importance (UNCRPD, 2007).
	Access and participation in religious activities	Provision of religious and spiritual activities in accessible formats (e.g., making prayers, songs, chanting, and sermons accessible with signed translation, and making religious texts available in large print, audio and Braille), accessibility of places of worship and reasonable accommodations in religious practices (e.g., inclusive services) (UNCRPD, 2007).
Recreation, leisure and sports	Access and participation in sports events	Strategies that encourage people with disabilities to have access and provide opportunities to participate in mainstream sporting activities at all levels through inclusive sports event; have an opportunity to organise, develop and participate in disability‐specific sporting and recreational activities through provision of support and links with OPDs for people with disabilities, assisting them to develop strategic, national and international partnerships and have access to adapted sports equipment (Khasnabis, 2010)
	Access and participation in recreation and leisure	Strategies that encourage people with disabilities to have access and provide opportunities to participate in mainstream sporting activities, recreation, tourism and leisure whether as a participant or observer (Khasnabis, 2010)
Justice	Accessibility^a^ of legal system and justice	Support with access the legal system and justice, for instance through Examples include accessible built infrastructure of courts and police stations (e.g., such as ramps, etc.)
	Access to legal system and justice	Suppor to access the systems, procedures, information, and locations used in the administration of justice (Lord & Stein, 2008) This includes activities such as legal awareness through OPDsDPOs and media, legal aid.
Assistive Technology and rehabilitation	Assistive Technology (AT)	Assistive technology is an umbrella term covering the systems and services related to the delivery of assistive products and services. Assistive products maintain or improve an individual's functioning and independence, thereby promoting their well‐being (e.g., wheelchairs, hearing aids).
	Rehabilitation	Rehabilitation is a process intended to eliminate or at least minimise – restrictions on the activities of people with disabilities, permitting them to become more independent and enjoy the highest possible quality of life (Bailey; Angell, 2005). This will include physiotherapy, occupational therapy and psychological support activities as provision of mobility, hearing, visual devices, and therapies to use these devices.
	Medical care	Provision of medical services to ensure that people with disabilities can access services designed to identify, prevent, minimise and/or correct health conditions and impairments (Khasnabis, 2010).
Policies and programmes	International legislations and policies	International legislations and policies through which countries abolish discrimination against persons with disabilities and eliminate barriers towards the full enjoyment of their rights and their inclusion in society (UN, 2018)
	Social inclusion policies	Inclusive policies on employment, educational and provision of housing and accommodation to people with disabilities.

^a^ ‘Accessibility’, in this publication refers to a feature or quality of any physical or virtual environment, space, facility or service that is capable of accommodating the needs of people with disabilities to understand, get access to or interact with legal system. Accessibility also refers to technical standards that are mandated nationally or internationally for the design and construction of a physical or virtual environment, space, facility and service

CBR's social pillar consists of five components:

- Personal assistance: Personal assistance may be helpful as many people with disabilities have impairments and functional difficulties that make it difficult to carry out activities and tasks independently in their current environment. Personal assistance interventions include formal and informal personal assistance and support and personal assistance training (UNCRPD, 2017).
- Relationship, marriage and family: Achieving social inclusion necessitates supporting people with disabilities to establish relationships, marry and become parents if they choose. This may require peer support, social networks, appropriate living conditions, community facilities and violence prevention interventions (UNCRPD, 2017).
- Culture and arts: People with disabilities must be free to access cultural materials in accessible formats; television programmes, films, theatre and other cultural activities in accessible formats; places for cultural performances or services, such as theatres, museums, cinemas, libraries and tourism services. The interventions to support this goal range from inclusive art education, sing‐language interpreters, cultural programme, theatres, arts and dramas, complementary therapy in the form of art and music and participation in religious activities (UNCRPD, 2017).
- Recreation, leisure and sports: People with disabilities should be enabled to participate actively and as spectators in recreational, leisure and sporting activities on an equal basis with others. Relevant interventions include networking and capacity building, organisation of inclusive sports events, provision of adapted sports equipment, recreation and sports clubs, community concerts and media, and sports‐based disability programme (UNCRPD, 2017).
- Justice: People with disabilities must have access to justice on an equal basis to ensure full enjoyment and respect of human rights. Interventions include inheritance rights and provision of procedural and age‐appropriate accommodations as witnesses in all legal proceedings at investigative and other stages. (UNCRPD, 2017).

We have added two additional categories to the CBR framework social pillar, which are relevant to promoting social inclusion: (1) Assistive Technologies (AT), Rehabilitation, and (2) Policies. We will consider interventions that specifically target people with disabilities, as well as mainstream programmes that are inclusive of people with disabilities.

### 2.3 How the intervention might work

For people with disabilities to be able to participate fully in society, it is imperative to consider the barriers that prevent them from doing so. People with disabilities are not a homogenous group, and the reasons for exclusion will vary for women and men in different settings and people with different impairment types. It is important to note that barriers are often experienced at three levels: the individual, the community, and the system.

#### 2.3.1 Individual‐level barriers

There are a variety of barriers to social inclusion at the individual level, including poor social and communication skills, a lack of assistive devices, a lack of personal assistance and support, or a lack of access to adapted equipment (e.g., for sport). Difficulties with social and communication skills—associated with some impairments—may also result in people with disabilities being excluded due to ableist norms around social behaviours and communication. Individuals with disabilities can suffer from internalised stigma, which can impact their dignity and confidence, limiting their chances of establishing relationships, expressing their sexuality, marrying, and having children. Social inclusion will be further hampered for people with disabilities who live in segregated or institutionalised housing or are constrained within their homes.

#### 2.3.2 Community‐level barriers

Among the community's barriers are physical barriers (e.g., inaccessible transport and buildings such as community centres and sports facilities) and informational barriers (e.g., non‐availability of sign‐language interpreters at cultural events), negative attitudes and beliefs among the community towards the participation of people with disabilities, as well as a lack of advocacy and volunteer groups (Organizations of People with Disabilities [OPDs]).

#### 2.3.3 System‐level barriers

Among the system‐level barriers are inadequate resource allocation to facilitate social inclusion for people with disabilities (e.g., personal assistance, supported independent living), the lack of legislation and policies that affirm the rights of people with disabilities to social inclusion, and the lack of inclusion of people with disabilities in decision‐making processes. Existing laws and regulations that require accessible programmes and activities are not recognised or enforced.

**Figure 1:**
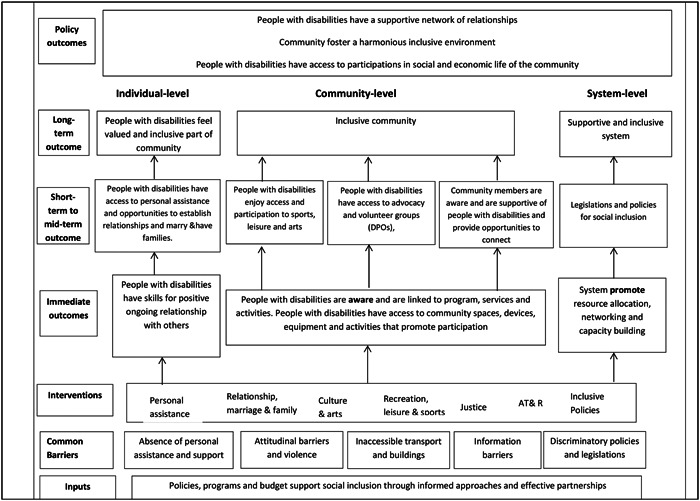
Logic model on interventions for improving social inclusion of people with disabilities.

To improve the social inclusion and outcomes of people with disabilities, addressing the barriers they encounter is necessary. In other words, they must operate at the level of the individual (e.g., personal assistance training and support), community (e.g., access to buildings such as community centres and recreation centres), and system (e.g., improving policy and legislation) (Figure 1).

#### 2.3.4 Individual‐level interventions

At the individual level, interventions may include the provision of mobility aids, communication aids, or assistive devices, rehabilitation and treatment, as well as personal assistance and transportation support as necessary. Raising awareness for and supporting the autonomy of people with disabilities is critical. Some interventions for people with disabilities may also focus on social and communication skills training, working under the assumption that improved social and communication skills may increase people with disabilities’ social capital and so opportunities to participate and advocate for their own inclusion.^1^

#### 2.3.5 Community‐level interventions

Community‐level interventions include adaptations to buildings and transportation that are accessible, services and programmes that are accessible to people with disabilities, as well as interventions to promote awareness, reduce stigma, and prevent violence, including raising awareness in the community through media, establishing links with local stakeholders to facilitate support, providing access to health care services, mainstreaming education, sports, recreation and leisure, as well as creating a welcoming and inclusive community.

#### 2.3.6 System‐level interventions

System‐level interventions include effective legislation to promote and protect social inclusion, such as inheritance rights, budget allocation for personal assistance, inclusive events, and media awareness of the rights of people with disabilities.

Programmes or activities may aim to operate at different levels concurrently. For example, FANDIC (Friends of Children with Disability for their Integration into the Community) intended to provide unique opportunities to develop physical and artistic abilities (individual‐level), to integrate children with disabilities into the community (community‐level), also provide unique opportunities to develop physical and creative skills (community‐level). It may involve coordination to increase awareness about disability at various levels, including the individual, community, organisational and governmental (system‐level).

### 2.4 Why it is important to do this review

Social inclusion of people with disabilities is recognised as a fundamental right in the UNCRPD, including in ‘participation in cultural life, recreation, leisure, and sport’ (article 30) and in participation in political and public life (Article 29). Furthermore, without social inclusion other rights (e.g., right to education) may not be realised. Social inclusion is also fundamental to implementing the 2030 Agenda; as long as people with disabilities are excluded from equal participation in all aspects of life, the SDGs arguably cannot be achieved. The wider society also benefits from the valuable contributions that people with disabilities make. Promoting social inclusion for people with disabilities, will also mean that ‘People with disabilities have meaningful social roles and responsibilities in their families and communities, and are treated as equal members of society’ (Khasnabis, 2010).

Several relevant systematic reviews and protocols exist that are relevant to the topic, but none which addresses the stated objectives of this review. Two reviews focussed on components of social inclusion. Almerie et al. (2015) conducted a review of social skills programmes for people with schizophrenia and identified 13 randomised controlled trials (RCTs) (Almerie et al., 2015). They concluded that social skills training may be effective at improving the social skills of people with schizophrenia, but that the data is limited and of very low quality. A systematic review of the effectiveness of interventions to prevent and respond to violence against persons with disabilities (Mikton et al., 2014). They identified 10 eligible studies, of which only one was from an LMIC. The studies were rated as poor quality, and the authors concluded ‘The current evidence base offers little guidance to policymakers, program commissioners, and persons with disabilities for selecting interventions’. Velema and colleagues assessed the evidence for effectiveness of rehabilitation‐in‐the‐community programmes, and concluded that CBR activities result in social processes that change the way community members view persons with disabilities, increase their level of acceptance and social inclusion and mobilise resources to meet their needs. However, the individual studies included in the review did not focus on improving social inclusion (Velema et al., 2008). Finally, a rapid Evidence Assessment of ‘What Works’ to Improve Social Inclusion and Empowerment for People with Disabilities in Low and Middle Income Countries was undertaken, but did not constitute a full systematic review. Therefore, currently a lack of evidence from LMICs on the effectiveness of interventions on adopting a disability inclusive approach to development, even in the presence of international efforts and despite the benefits of social inclusion. These outcomes remain complex and difficult to quantify (Walton, 2012). Hence, evidence on ‘what works’ to improve social inclusion of people with disabilities is needed to inform policy, practice, and further research.

## 3 OBJECTIVES

The objectives of this review are to (1) examine the effectiveness of interventions for improving social inclusion outcomes for people with disabilities (physical, visual, hearing, intellectual, or mental health conditions) in LMICs; and (2) to critically appraise the confidence in study finding of the included studies.

Key questions include:

1. Are interventions to improve social inclusion outcomes for people with disabilities in LMICs effective? What is the confidence level in the evidence base that supports these interventions?
2. What types of intervention, or intervention design features, are most effective in improving social inclusion outcomes for people with disabilities in LMICs?
3. What interventions appear to be most effective for people with different types of impairment?

## 4 METHODS

### 4.1 Criteria for considering studies for this review

#### 4.1.1 Types of studies

For this review, the primary research designs of interest were experimental and quasi‐experimental study designs and non‐randomised studies with a control group, including controlled before‐and‐after (CBA). We included studies using the following study designs:

(a) participants are randomly assigned (using a process of random allocation, such as a random number generation),
(b) a quasi‐random method of assignment has been used,
(c) participants are non‐randomly assigned but matched on pre‐tests and/or relevant demographic characteristics (using observables or propensity scores) and/or according to a cut‐off on an ordinal or continuous variable (regression discontinuity design),
(d) participants are non‐randomly assigned, but statistical methods have been used to control for differences between groups (e.g., using multiple regression analysis or instrumental variables’ regression),
(e) the design attempts to detect whether the intervention has had an effect more significant than any underlying trend over time, using observations at multiple time points before and after the intervention (interrupted time‐series design),
(f) participants receiving an intervention are compared with a similar group from the past who did not (i.e., a historically controlled study), or
(g) observations are made on a group of individuals before and after an intervention, but with no control group (single‐group before‐and‐after study).

#### 4.1.2 Types of participants

The target populations are people with disabilities living in LMICs. Population subgroups of interest include: women, vulnerable children (particularly children in care), conflict (conflict and post‐conflict settings), migrants, ethnic minority groups and people with different impairment types including visual impairment, hearing impairment, physical impairment and intellectual impairment. Studies with multiple populations were included if one of the population subgroups is people with disabilities.

#### 4.1.3 Types of interventions

The goal of WHO's Community Based Rehabilitation (CBR) social component is that people with disabilities have meaningful social roles and responsibilities in their families and communities and are treated as equal member of the society. It focusses on improving social inclusion, which can be achieved through the intervention categories listed below:

#### 4.1.4 Types of outcome measures

Eligible outcomes will relate to the social inclusion pillar of the CBR matrix. The outcome of interest include outcomes listed in Table 2.

**Table 2:** Outcome and outcome sub‐categories.

Outcome category	Outcome sub‐category	Description
Skills for social inclusion	Social and communication skills	Social skills as learned verbal and non‐verbal behaviour performed within a specific social context of an aggressiveness‐shyness continuum, and view adjustment in relation to an individual's social perceptual accuracy (i.e., the ability to understand subtle nuances and define critical elements in social environment) (Kratochwill & French, 1984). Communication skills is the ability to transfer information. It may be vocally (using voice), written (using printed or digital media such as books, magazines, websites or emails), visually (using logos, maps, charts or graphs) or non‐verbally (using body language, gestures and the tone and pitch of voice). This includes availability and use of communication aids and speech and reading devices
	Social behaviour	Social behaviour can be defined as all behaviour that influences, or is influenced by, other members of the same species. The term thus covers all behaviour that tends to bring individuals together as well as all forms of aggressive behaviour (Grant, 1963). This includes conduct problems, peer problems, pro‐social behaviours.
Broad based social inclusion and participation measure	Social inclusion and community participation	Social inclusion is defined as the process of improving the terms of participation in society, particularly for people who are disadvantaged, through enhancing opportunities, access to resources, voice and respect for rights. (UN, 2010). These will include measures such as people with disabilities spending more time out of the house, and travelling further away from the house and a greater number and depth of social interactions. People with disabilities have access, accessibility and opportunities to participate in community activities such as leisure activities, such as hobbies, arts, and sports, political and civic activities or organisations and productive activities, like employment or education; consumption, or access to goods and services; religious and cultural activities and groups.
	Access to justice	People with disabilities get access to or interact with legal system
Relationships	Interpersonal and Family relationship	People with disabilities have strong relationships with family members, staff, friends, acquaintances, and intimate partners (Clarkson et al., 2009) and other people with disabilities, and feeling a sense of belonging to a network when they have different people fulfilling different needs (McVilly et al., 2006). This also includes aspects of participation in household, behaviour of the family towards people with disabilities
	Peer and community relationships	People with disabilities have meaningful relationships, marry and have children. (Community‐Based Rehabilitation: CBR Guidelines)
	Violence and abuse	People with disabilities are protected against violence, and all relevant stakeholders work together to address the issue. (Community‐Based Rehabilitation: CBR Guidelines)

##### Duration of follow‐up

Any duration of follow‐up was included.

##### Types of settings

All settings were eligible, provided that the study is situated within a LMIC, as defined by the World Bank, 2018 (https://datahelpdesk.worldbank.org/knowledgebase/articles/906519-world-bank-country-and-lending-groups).

### 4.2 Search methods for identification of studies

The search for this systematic review is based on the searches performed for the evidence and gap map on interventions for people with disabilities in LMICs (Saran et al., 2020). The EGM presents studies on the effectiveness of interventions for people with disabilities in LMICs. We updated the database search in February 2020 and screened the references to identify additional studies (Supporting Information: Appendix 1). To identify any relevant articles that may have been missed during the EGM processes, we ran the searches with search terms specific to social inclusion review using Open Alex in EPPI reviewer (Thomas & Stansfield, 2018).

#### 4.2.1 Electronic searches

The authors searched the following electronic databases.

- MEDLINE(R)
- Embase Classic+Embase
- PsycINFO
- CAB Global Health
- CINAHL
- ERIC
- Scopus
- Web of Science (Social Sciences Citation Index)
- WHO Global Health Index
- MEDLINE
- Embase
- PsychINFO
- CAB Global Health
- OVID
- ERIC
- CINAHL
- Ebsco
- PubMED

Search strategies were tailored for each of the databases.

#### 4.2.2 Searching other resources

To maximise the coverage of unpublished and grey literature and minimise publication bias, we searched the following organisation's websites and databases using keyword searches.

- ILO
- DFID (including Research for Development [R4D])
- UNESCO
- WHO
- Disability Programme of the UN Economic and Social Commission for Asia and the Pacific (UNSCAP)
- United States Agency for International Development (USAID)
- Dissertation Abstracts, Conference Proceedings and Open Grey.
- Humanity and Inclusion (HI) http://www.hi-us.org/publications
- CBM https://www.cbm.org/Publications-252011.php
- Plan international https://plan-international.org/publications

We also ran the searches with search terms specific to social inclusion review using Open Alex in EPPI reviewer 4.

### 4.3 Data collection and analysis

#### 4.3.1 Description of methods used in primary research

**Figure 2:**
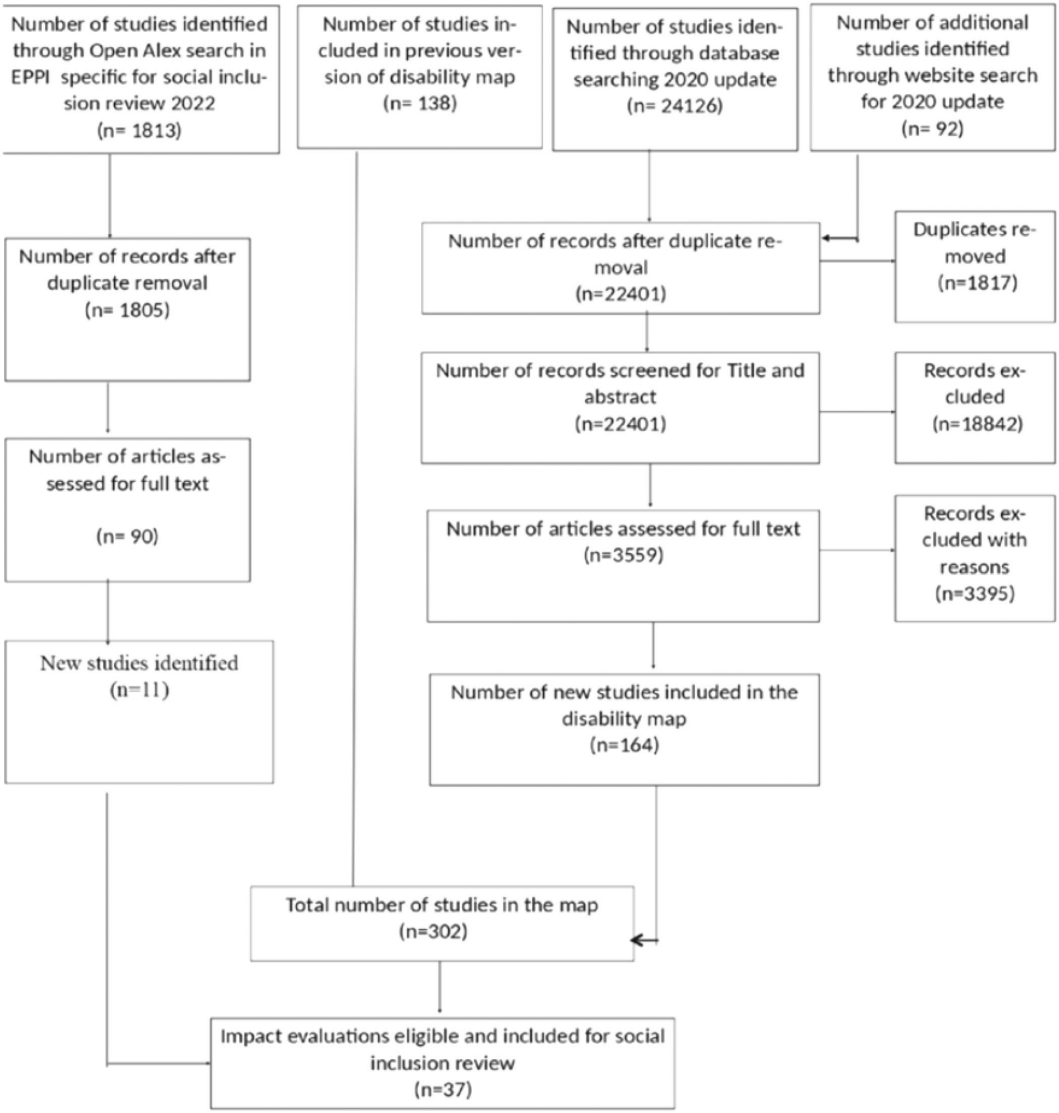
PRISMA flowchart.

The titles and abstracts of all documents included were screened by two independent reviewers using EPPI Reviewer 4. Two reviewers evaluated the full texts of studies that met or appeared to meet the inclusion criteria. If there were any disagreements, they were resolved in discussion until a consensus was reached. The flow of studies through the screening process is documented in a PRISMA flow chart (Figure 2).

#### 4.3.2 Criteria for determination of independent findings

Before initiating the synthesis (detailed below), we ensured that all articles reporting on the same study were appropriately linked, as several articles could be published using data from the same sample. Furthermore, studies can report multiple outcomes, in which case we selected the most relevant measure for analysis using the following decision rules:

Outcomes measured via validated formal scales are more relevant than those measured using a single‐item question. We only extracted data on the intervention and control groups eligible for this review for studies with multiple intervention arms. Should a multi‐arm study report multiple relevant intervention arms, the findings from the different arms were reported and analysed separately.

#### 4.3.3 Selection of studies

Two review authors independently screened the articles for title and abstract and full‐text with a third‐party arbiter in case of disagreement.

#### 4.3.4 Data extraction and management

Two review authors independently extracted the necessary data from each study report. Data and information were extracted on available characteristics of participants, intervention characteristics and control conditions, research design, sample size, risk of bias and outcomes, and results. Extracted data were stored electronically. The coding sheet for this review is included in Supporting Information: Appendix 2.

#### 4.3.5 Assessment of risk of bias in included studies

Two review authors independently assessed the risk of bias for each included study. We resolved any disagreements by discussion or by involving a third review author. We evaluated the risk of bias according to the following domains. Confidence in study findings was rated high, medium, or low for each criterion, applying the standards as shown in Supporting Information: Annex 3. Overall confidence in study findings was determined to be the lowest rating across the criteria—the weakest link in the chain principle.

- Study design
- Masking
- Presence of a power calculation
- Attrition, which we applied
- Clear definition of disability
- Clear definition of outcome
- Baseline balance

#### 4.3.6 Measures of treatment effect

Effect size estimates with 95% confidence intervals (CIs) were extracted from included studies. Effect sizes were measured as SMDs with their 95% CIs. In all studies, treatment effects were reported as continuous outcomes. Treatment effects were estimated using SMDs for RCTs and quasi‐experiments with two independent groups by entering the required data into metafor package in R (M, SD, *n*). SMDs were calculated using baseline‐adjusted mean differences (i.e., mean change scores) in studies reporting baseline and post‐intervention outcome data. The formulae for these effect sizes are presented in other Campbell reviews (Waddington et al., 2021).

#### 4.3.7 Unit of analysis issues

The unit of analysis of interest to the present review was individual people with disabilities, their caregivers, carers, or those working with them. If a study had more than two intervention arms, we included only intervention and control groups that met the eligibility criteria. Where multi‐arm studies were included, we ensured not to double‐count participants and separately reported eligible interventions and their respective outcomes.

#### 4.3.8 Dealing with missing data

Attrition was calculated for each study, and an evaluation was conducted to assess the overall quality of the study. No included study was eliminated from the analysis due to missing data.

#### 4.3.9 Assessment of heterogeneity

Heterogeneity analysis was conducted for participant, intervention, and outcome characteristics. Because multiple effect sizes may be attributable to sampling error, a random effects model and the associated inverse variance weight at the 95% confidence level was used for all analysis. The random effects model provides for an assumption of population variation from which the sample is drawn and calculates the effect size's impact by estimating that population's parameters. An *I* ^2^ of 0%–40% was interpreted to be low heterogeneity, 41%–80% moderate heterogeneity and 81% and above to mean high heterogeneity (Higgins et al., 2009).

#### 4.3.10 Assessment of reporting biases

Publication bias was assessed visually with funnel plots produced using the *metafor* package in R and tested more formally with Egger's meta‐regression test (Egger et al., 1997). A funnel plot involves plotting the effect size (horizontal axis) against the study's precision (vertical axis). There should be a symmetric distribution of effect sizes between the different studies without publication bias (the vertical line in the centre). In theory, studies with a low degree of precision (at the bottom of the graph) will deviate more from the pooled effect size than studies with a high degree of precision (at the top of the graph), creating a funnel distribution. An asymmetric funnel plot indicates publication bias. Egger's test involves a linear regression between the intervention effect estimates and their standard errors weighted by the inverse variance (Egger et al., 1997).

#### 4.3.11 Data synthesis

Coding included: (1) basic study characteristics, (2) narrative summary (including annotation of any adverse effects), (3) summary of findings/results table, and (4) assessment of confidence in study findings. This coding was conducted by pairs of coders, with comparison and discussion to resolve discrepancies. There was a 93% agreement rate between coders for the study characteristics. Data were extracted from the studies using an extraction form piloted before use. After coding each study and extracting/calculating each effect size, the *metafor* package in R was used to conduct random effects inverse variance meta‐analyses with 95% CIs. The magnitudes of the mean effect size (SMD) were then interpreted according to Cohen's (1988) convention: *d* < 0.2 (small effect size), *d* = 0.2–0.6 (moderate effect size), and *d* > 0.6 (large effect size) (Cohen, 1988).

#### 4.3.12 Subgroup analysis and investigation of heterogeneity

The review protocol outlined the intended approach for investigating heterogeneity through specific sub‐group analyses. Subgroup analysis was only conducted for the type of impairment because of insufficient information on settings, socio‐economic status and gender of the target group.

#### 4.3.13 Sensitivity analysisliag

#### 4.3.14 Treatment of qualitative research

We did not include qualitative research.

##### Summary of findings and assessment of the certainty of the evidence

Findings of the review were summarised and the certainty of the evidence was assessed as outlined in the protocol Saran et al., 2021.

## 5 RESULTS

### 5.1 Description of studies

#### 5.1.1 Results of the search

The Preferred Reporting Items for Systematic Reviews and Meta‐Analyses (PRISMA) flowchart (Figure 2) outlines the steps in the review process. The electronic database searches for the EGM yielded 24,126 potentially relevant documents for review; additional 92 studies were identified from the grey literature search, reference and citation searching. The results from all three searches were combined, exported, and deduplicated using the reference management software EPPI reviewer 4, and we identified 1817 duplicates. We reviewed the titles and abstracts of the remaining 22,401 documents to determine potential relevance, excluding 18,842 due to irrelevance to the review, leaving 3559 articles for full paper review to determine inclusion. Of these, 3395 were excluded, and 164 new studies were deemed relevant for the updated review. These 164 were pooled with the 138 studies identified from the previous EGM search, bringing the total count of included studies for this effectiveness map to 302. Of these 302, 26 impact evaluations were found eligible for inclusion in the social inclusion review.

As noted in the methods section, to identify any relevant articles that may have been missed during the EGM processes, we also ran the searches with search terms specific to social inclusion review using Open Alex in EPPI reviewer. We identified an additional 1813 studies, the results were deduplicated, and we identified 1805 studies that were screened for title and abstract. Only 90 studies were included for full‐text review. Only eleven studies were included for data extraction from this update, totalling 37 included studies in this review.

#### 5.1.2 Included studies

We identified 37 studies as meeting the inclusion criteria. The characterstics of included studies is detailed in Supporting Information: Appendix 3.

##### Participant characteristics

###### Target group

Twenty‐three studies targeted children with disabilities (Abazari et al., 2017; Dai et al., 2018; De Villiers et al., 2013; Devries et al., 2018; Esmaili et al., 2019; Golzari et al., 2015; Juneja et al., 2012; Kalgotra & Warwal, 2017; Karanth et al., 2010; Koo & Thomas, 2019; Lal, 2010; Lee et al., 2019; Liang et al., 2022; Manohar et al., 2019; McConachie et al., 2000; Nair et al., 2014; Pajareya & Nopmaneejumruslers, 2011; Pop et al., 2013; Rahman et al., 2016; Shin et al., 2009; Shore & Juillerat, 2012; Wang, 2008; Zuurmond et al., 2018). Twelve studies targeted adults with disabilities (Amaresha et al., 2018; Govindaraj et al., 2018; Hanlon et al., 2020; Karaman et al., 2020; Khalil et al., 2019; Li et al., 2018; Lund et al., 2013; Rami et al., 2018; Ran et al., 2003; Ravindren et al., 2018; Shore & Juillerat, 2012; Yildiz et al., 2004). Two studies specifically targeted family members and caregivers (Azari et al., 2019; Rahmani et al., 2015). All interventions targeted both men and women. Twelve studies specifically noted participants’ socioeconomic status.

###### Impairment groups

Seventeen studies reported on interventions which targeted individuals with a single type of impairment, with only one reporting on an intervention for people with a range of impairments (Devries et al., 2018). Twenty studies targeted people with intellectual disabilities (Abazari et al., 2017; Azari et al., 2019; Dai et al., 2018; Devries et al., 2018; Esmaili et al., 2019; Golzari et al., 2015; Juneja et al., 2012; Kalgotra & Warwal, 2017; Karanth et al., 2010; Koo & Thomas, 2019; Lal, 2010; Lee et al., 2019; Liang et al., 2022; Manohar et al., 2019; Nair et al., 2014; Pajareya & Nopmaneejumruslers, 2011; Pop et al., 2013; Rahman et al., 2016; Shin et al., 2009; Wang, 2008). Thirteen studies reported on interventions which targeted people with psychosocial/mental impairments (Amaresha et al., 2018; Govindaraj et al., 2018; Hanlon et al., 2020; Karaman et al., 2020; Khalil et al., 2019; Li et al., 2018; Li et al., 2019; Lund et al., 2013; Rahmani et al., 2015; Rami et al., 2018; Ran et al., 2003; Ravindren et al., 2018; Yildiz et al., 2004). Five studies reported on interventions which targeted people with physical impairments (De Villiers et al., 2013; Devries et al., 2018; McConachie et al., 2000; Shore & Juillerat, 2012; Zuurmond et al., 2018).

###### Geographical setting of the intervention

Eight studies (Amaresha et al., 2018; Dai et al., 2018; Devries et al., 2018; Kalgotra & Warwal, 2017; Li et al., 2018; Rami et al., 2018; Ravindren et al., 2018; Shore & Juillerat, 2012) reached a mix of rural and urban participants, and fifteen explicitly targeted urban populations (Azari et al., 2019; Khalil et al., 2019; Lee et al., 2019; Manohar et al., 2019). Four studies explicitly reported targeting rural populations (Hanlon et al., 2020; Lund et al., 2013; McConachie et al., 2000; Ran et al., 2003). In ten of the studies, the setting was not reported; in a few of these, whether rural or urban settings were covered was unclear from the districts in which the intervention took place.

###### Country

**Figure 3:**
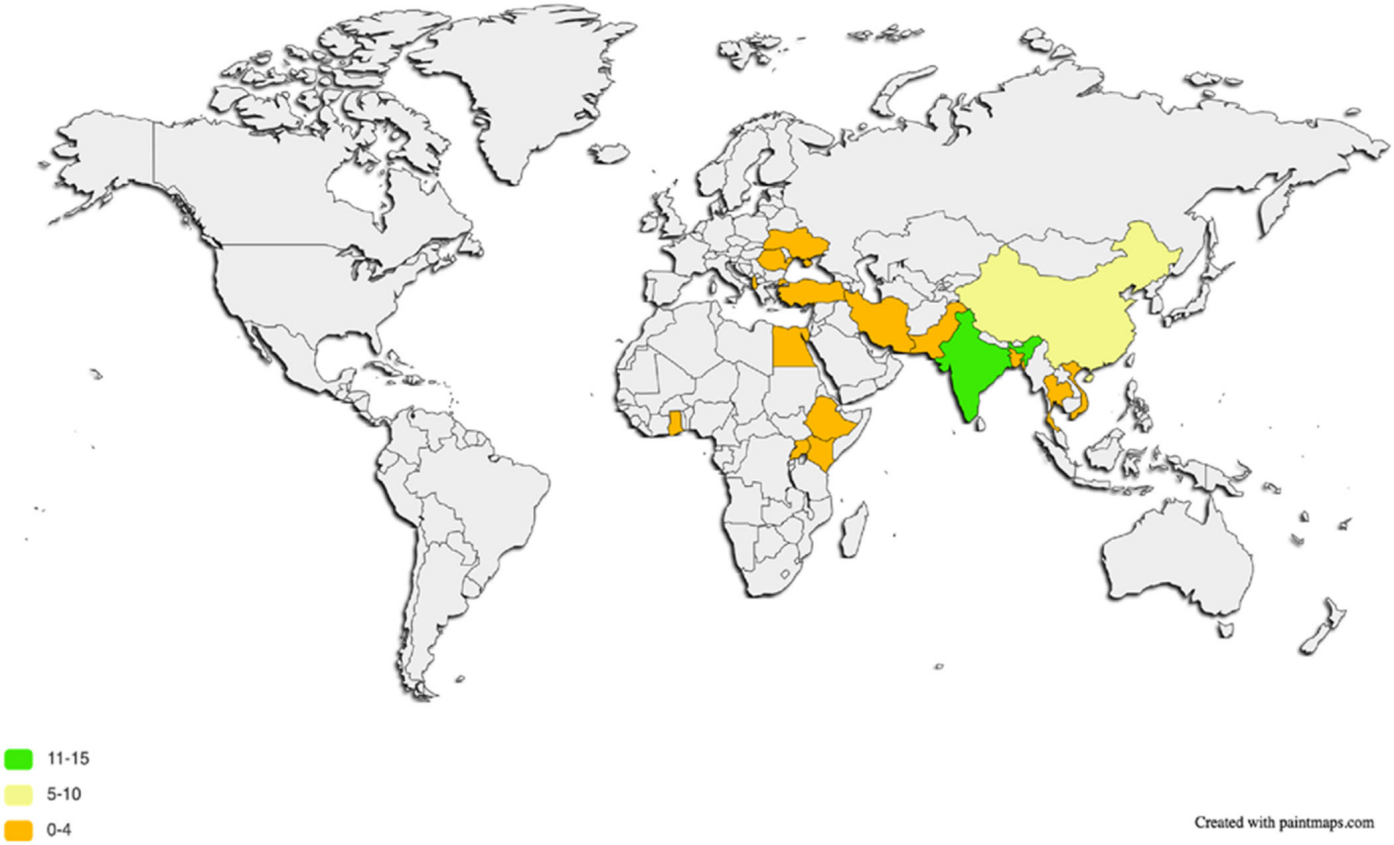
Distribution of impact evaluations.

Studies were conducted in 16 different countries with 12 studies conducted in India (Amaresha et al., 2018; Govindaraj et al., 2018; Juneja et al., 2012; Kalgotra & Warwal, 2017; Karanth et al., 2010; Koo & Thomas, 2019; Lal, 2010; Manohar et al., 2019; Nair et al., 2014; Rahman et al., 2016; Ravindren et al., 2018; Shore & Juillerat, 2012), 6 studies conducted in China (Lee et al., 2019; Li et al., 2018; Li et al., 2019; Liang et al., 2022; Ran et al., 2003; Wang, 2008), 5 studies conducted in Iran (Abazari et al., 2017; Azari et al., 2019; Esmaili et al., 2019; Golzari et al., 2015; Rahmani et al., 2015) and 2 in Turkey (Karaman et al., 2020; Yildiz et al., 2004. Only one study each was reported in Albania (Dai et al., 2018), Ghana (Lund et al., 2013; Zuurmond et al., 2018; Romania (Pop et al., 2013), South Africa (De Villiers et al., 2013), Thailand (Pajareya & Nopmaneejumruslers, 2011), Uganda (Devries et al., 2018), Vietnam (Shin et al., 2009), Bangladesh (McConachie et al., 2000), Pakistan (Rahman et al., 2016), Ethiopia (Hanlon et al., 2020) (Figure 3).

##### Study characteristics

###### Study design

The research designs of the studies included 18 RCT [Azari et al., 2019; Devries et al., 2018; Esmaili et al., 2019; Karaman et al., 2020; Karanth et al., 2010; Li et al., 2018; Li et al., 2019; Liang et al., 2022; Manohar et al., 2019; McConachie et al., 2000; Pajareya & Nopmaneejumruslers, 2011; Pop et al., 2013; Rahman et al., 2016; Rami et al., 2018; Ran et al., 2003; Shin et al., 2009; Wang, 2008], 13 quasi‐randomised controlled trial [Abazari et al., 2017; Amaresha et al., 2018; Dai et al., 2018; De Villiers et al., 2013; Hanlon et al., 2020; Juneja et al., 2012; Kalgotra & Warwal, 2017; Khalil et al., 2019; Koo & Thomas, 2019; Lal, 2010; Lund et al., 2013; Nair et al., 2014; Yildiz et al., 2004], six uncontrolled before and after studies [Govindaraj et al., 2018; Lee et al., 2019; Rahmani et al., 2015; Ravindren et al., 2018; Shore & Juillerat, 2012; Zuurmond et al., 2018].

###### Intervention characteristics

Many of the interventions were multi‐component and fell into several categories. The details and implementation of interventions for each study are described in Supporting Information: Appendix 2. Ten studies aimed at providing personal assistance and support and evaluated the effects of a parent training programme on the interactive skills of parents of children with disabilities [Azari et al., 2019; Dai et al., 2018; Li et al., 2018; Manohar et al., 2019; Pajareya & Nopmaneejumruslers, 2011; Rami et al., 2018; Ran et al., 2003; Shin et al., 2009; Wang, 2008; Zuurmond et al., 2018].

Seventeen studies evaluated impact of social skills training programmes on enhancing social and communication skills of people with disabilities (Abazari et al., 2017; Esmaili et al., 2019; Golzari et al., 2015; Juneja et al., 2012; Karaman et al., 2020; Karanth et al., 2010; Khalil et al., 2019; Lal, 2010; Lee et al., 2019; Li et al., 2018; Liang et al., 2022; McConachie et al., 2000; Nair et al., 2014; Pop et al., 2013; Rahman et al., 2016; Ravindren et al., 2018; Yildiz et al., 2004).

Two studies aimed at improving community attitude through community‐based comprehensive intervention programmes and anti‐stigma training [Li et al., 2018, 2019]. Only one study aimed at preventing and reducing violence against children with disabilities and evaluated The Good School Toolkit as an effective intervention to reduce violence perpetrated by peers and school staff against young adolescents with disabilities [Devries et al., 2018].

Two included programmes aimed at increasing access and participation in cultural programmes, arts, drama and theatres; one [Kalgotra & Warwal, 2017] evaluated the effect of music intervention on behaviour disorders and the other evaluated impact of art therapy sessions on improving social skills of children with Autism Spectrum Disorder [Koo & Thomas, 2019].

Three studies aimed at increasing access and participation in recreation and leisure [De Villiers et al., 2013; Esmaili et al., 2019; Govindaraj et al., 2018]. Three aimed at improving the social inclusion of people with disabilities by improving access to rehabilitation [Amaresha et al., 2018; Lund et al., 2013], provision of wheelchairs [Shore & Juillerat, 2012] and provision of integrated district‐level mental health care (Hanlon et al., 2020).

There were several categories of possible intervention, including access and participation in religious activities, access and participation in sports events, access to justice and policy change, where no eligible studies were identified. Almost all the studies evaluated the effectiveness of interventions targeted at the individual level, i.e. people with disabilities and their family members (e.g., improving social skills). Two studies aimed at improving community attitude through community‐based comprehensive intervention programmes and anti‐stigma training (Li et al., 2019; Li et al., 2018), and one study aimed at preventing and reducing violence against children with disabilities (Devries et al., 2018) There were no studies evaluating system‐level interventions (e.g., policy change).

##### Outcome characteristics

Table 3 shows these categories, with corresponding descriptions.

**Table 3:** Outcome categories and sub‐categories.

Outcome Category	Examples of outcome sub‐types included
Skills for social inclusion	Social skills
	Social behaviour
Relationships	Personal assistance
	Interpersonal and family relationship
	Violence and abuse
	Peer and community relationship
Broad‐based social inclusion	Social inclusion including community participation
	Access to justice

Most outcomes fell into the category of improving social skills, with 13 studies examining social and communication skills [Abazari et al., 2017; Esmaili et al., 2019; Golzari et al., 2015; Govindaraj et al., 2018; Kalgotra & Warwal, 2017; Karanth et al., 2010; Lal, 2010; Lee et al., 2019; Liang et al., 2022; Nair et al., 2014; Pajareya & Nopmaneejumruslers, 2011; Rahman et al., 2016; Shin et al., 2009] and 12 social behaviour [Abazari et al., 2017; Esmaili et al., 2019; Govindaraj et al., 2018; Karanth et al., 2010; Khalil et al., 2019; Lal, 2010; Lee et al., 2019; Li et al., 2018; Liang et al., 2022; Pajareya & Nopmaneejumruslers, 2011; Rami et al., 2018; Yildiz et al., 2004]. Thirteen studies reported relationship outcomes, of which six examined interpersonal and family relationship (Abazari et al., 2017; Amaresha et al., 2018; McConachie et al., 2000; Rahmani et al., 2015; Wang, 2008; Zuurmond et al., 2018) and seven on personal assistance (Azari et al., 2019; Dai et al., 2018; Manohar et al., 2019; McConachie et al., 2000; Rahmani et al., 2015; Ran et al., 2003; Zuurmond et al., 2018). Nine studies assessed broad‐based social inclusion (De Villiers et al., 2013; Karanth et al., 2010; Khalil et al., 2019; Li et al., 2018; Rami et al., 2018; Ran et al., 2003; Shore & Juillerat, 2012; Yildiz et al., 2004; and Zuurmond et al., 2018). We found only one study assessing violence and abuse (Devries et al., 2018) and peer and community relationship (Hanlon et al., 2020). No studies were identified on access to justice.

#### 5.1.3 Excluded studies

Excluded studies with the associated reason for exclusion are presented in Supporting Information: Appendix 4. The most common reasons for exclusion were that the study was not an impact evaluation, presented a protocol for which there were no associated results, focused on an ineligible population, had a social inclusion intervention but no social inclusion outcomes, provided only qualitative data, and—in one case—otherwise relevant findings were not disaggregated for people with disabilities.

### 5.2 Risk of bias in included studies

Overall there is low confidence in the study findings for 27 of the 37 studies (Table 4). Five studies [Juneja et al., 2012; Karaman et al., 2020; Manohar et al., 2019; McConachie et al., 2000; Rahman et al., 2016] scored medium using our assessment tool. We found five studies [Amaresha et al., 2018; Esmaili et al., 2019; Pajareya & Nopmaneejumruslers, 2011; Rami et al., 2018; Yildiz et al., 2004] that scored high confidence in the findings. There is diversity within low ratings as we employed the weakest link in the chain principle to assess confidence in study findings (Supporting Information: Appendix 3). However, the findings of a study receiving a low rating on a single item (e.g., for reporting of attrition) should not be treated in the same manner as those derived from a study rating low on multiple items. The latter approach allows for valuable learning not to be overlooked due to an overall ‘low’ confidence in study findings score, in studies which had many areas of strength.

**Table 4:** Critical Appraisal of included studies.

Study author and year	Study design (Potential confounders taken into account)	Blinding (RCTs only)	Loss to follow up are presented and acceptable	Disability impairment measure is clearly defined and reliable	Outcome measurement	Baseline Balance (N.A for before vs. after)	Overall confidence in study findings
Abazari et al. (2017)	low	Not applicable	Moderate	High	High	Not applicable	Low
Amaresha et al. (2018)	High	High	High	High	High	High	High
Azari et al. (2019)	High	Moderate	High	Low	High	High	Low
De Villiers et al. (2013)	Low	Not applicable	Low	Low	High	Not applicable	Low
Dai et al. (2018)	Low	Not applicable	High	High	High	Not applicable	Low
Devries et al. (2018)	High	Moderate	Low	High	High	High	Low
Esmaili et al. (2019)	High	High	High	High	High	High	High
Golzari et al. (2015)	Low	Not applicable	High	low	High	Not applicable	Low
Govindaraj et al. (2018)	Low	Not applicable	High	High	High	Not applicable	Low
Hanlon et al. (2020)	Low	Not applicable	High	High	High	Not applicable	Low
Juneja et al. (2012)	Moderate	Not applicable	High	High	High	Not applicable	Moderate
Kalgotra & Warwal (2017)	Low	Not applicable	High	High	High	Not applicable	Low
Karaman et al. (2020)	High	Moderate	High	High	High	High	Moderate
Karanth et al. (2010)	Low	Not applicable	low	High	High	Not applicable	Low
Khalil et al. (2019)	Low	Not applicable	High	High	High	Not applicable	Low
Koo & Thomas (2019)	Low	Not applicable	High	High	High	Not applicable	Low
Lal (2010)	low	Not applicable	High	Moderate	High	Not applicable	Low
Lee et al. (2019)	low	Not applicable	High	High	High	Not applicable	Low
Li et al. (2018)	High	Low	Moderate	High	High	High	Low
Li et al. (2019)	High	Low	Moderate	High	High	High	Low
Liang et al. (2022)	High	Low	Low	High	High	High	Low
Lund et al. (2013)	low	Not applicable	High	High	High	Not applicable	Low
Manohar et al. (2019)	High	Moderate	High	High	High	High	Moderate
McConachie et al. (2000)	High	High	Moderate	High	High	High	Moderate
Nair et al. (2014)	low	Not applicable	High	High	High	Not applicable	Low
Pajareya & Nopmaneejumruslers (2011)	High	High	High	High	High	High	High
Pop et al. (2013)	High	High	Low	High	High	High	low
Rahman et al. (2016)	High	Moderate	High	High	High	High	Moderate
Rahmani et al. (2015)	Low	Not applicable	Low	High	High	Not applicable	Low
Ravindren et al. (2018)	Low	Not applicable	High	High	High	Not applicable	Low
Rami et al. (2018)	High	High	High	High	High	High	High
Ran et al. (2003)	High	Low	High	High	High	High	Low
Shin et al. (2009)	High	Low	High	High	High	High	Low
Shore & Juillerat (2012)	Low	Not applicable	High	High	Moderate	Not applicable	Low
Wang et al. (2008)	High	Low	High	High	High	Low	Low
Yildiz et al. (2004)	High	High	High	High	High	High	High
Zuurmond et al. (2018)	Low	Not applicable	High	High	High	Not applicable	Low

#### 5.2.1 Appraisal by criterion

##### Study design

Nineteen studies were rated ‘low’ on study design as many used before and after designs. Eighteen studies were rated high in our assessment of confidence in study findings based on design, as they were randomised controlled trials.

##### Masking

Of the 18 randomised controlled trials, only 7 studies were rated as high and were masked for data collection (where feasible) and masking for analysis and 5 studies were rated medium they were masked for analysis. For six studies masking was not mentioned and was rated as low in our assessment of confidence in study findings.

##### Losses to follow‐up presented and acceptable

The issue of incomplete outcome data was not addressed adequately in six studies putting them at high risk of attrition bias due to significant loss to follow‐up from both intervention and control groups**.** Studies that were at low risk addressed incomplete outcome data adequately in 27 studies and 4 were assessed to be of medium‐risk of bias.

##### Disability/impairment measure definition and reliability

One of the areas which received relatively good ratings across studies was the use of disability/impairment measures or definitions which were consistently clear and reliable. Only four studies received a rating of ‘low’ and one a ‘medium’ rating. In all the remaining studies, rigorous and replicable criteria were used, and high ratings were given. Li et al. (2018) included individuals with psychosocial disabilities (schizophrenia) and the Brief Psychiatric Rating Scale was used to assess the participants’ psychiatric status. In the study Azari et al. (2019); a detailed measure of impairment, the Washington Group questionnaire was used.

##### Outcome measures definition and reliability

Outcome measures were largely well‐defined, perhaps reflective of the tendency of the studies to be outcome‐driven interventions, and so primarily concerned with operationalizing and then acting upon, a particular dimension of social inclusion. All but one study received high ratings on this item.

##### Baseline balance

As with masking, baseline balance was only relevant for the 18 studies, including the randomised controlled trial. Randomised controlled trial studies reported acceptable baseline balance and were coded as high on this item for all but one study.

### 5.3 Effects of interventions

#### 5.3.1 Synthesis of results

We conducted meta‐analysis with 37 studies, categorised into three outcome categories according to the pathway developed earlier in the study (Figure 1). Skills for social inclusion, relationships, and broad‐based social inclusion were the outcomes of interest. To conduct the meta‐analysis, we used independent estimates, I2 statistics, and their corresponding p‐values to determine the differences between the effect sizes across the different outcome types. For each outcome of interest, effect sizes are calculated using SMDs, which indicate changes in scores between the control and intervention groups. SMD scores are interpreted as the number of standard deviation changes in the outcome. Table 5, it is shown that high heterogeneity is evident in all three areas: social skills (*I* = 94%), social behaviour (*I* = 90%), and social inclusion (*I* = 93%). A relatively small number of studies were used in the analysis for personal assistance (*n* = 7), social inclusion (*n* = 9) and interpersonal relationships (*n* = 6). Only one study was identified on violence and peer relationships.

**Table 5:** Summary of findings.

	Outcome	Effect	Summary
Skills for social inclusion	Social skills	*d* = 0.80 (0.37–1.23) *k* = 13 *n* = 441 *I* ^2^ = 94% Egger's test 3.60 (*t* = 3.48, *p*< 0.01)	Large effect based on a moderate number of studies with high heterogeneity and a significant publication bias
	Social behaviour	*d* = 0.94 (0.50–1.38) *k* = 13 *n* = 1018 *I* ^2^ = 90% Eggers test 1.08 (*t* = 0.75, *p* = 0.46)	Large effect based on a moderate number of studies with high heterogeneity and no publication bias
Relationships	Personal assistance	*d* = 0.58 (0.25–0.90) *k* = 7 *n* = 650 *I* ^2^ = 59% Eggers test 1.37 (*t* = 1.38, *p* = 0.19)	Moderate effect based on low number of studies with medium heterogeneity and no publication bias
	Interpersonal and family relationship	*d* = 0.73 (0.34–1.13) *k* = 6 *n* = 294 *I* ^2^ = 72% Eggers test 1.36 (*t* = 1.58, *p*= 0.15)	Large effect based on low number of studies with medium heterogeneity and no publication bias
Broad‐based social inclusion	Social inclusion	*d* = 0.72 (0.33–1.11) *k* = 9 *n* = 1497 *I* ^2^ = 93% Eggers test 3.71 (*t* = 3.14, *p*= 0.01)	Large effect based on low number of studies with high heterogeneity and significant publication bias

*Note*: *d* < 0.2 small, 0.2 < *d* < 0.6 moderate and *d* > 0.6 large. *k* < 10 small, 10 ≤ *k* < 20 moderate and *k* ≥ 20 large (*k* = number of studies). *I* ^2^ < 0.4 low, 0.4 ≤ *I* ^2^ < 0.8 moderate and *I* ^2^ ≥ 0.8 high.

Abbreviation: *n*, total number of participants.

##### Skills for social inclusion

**Figure 4:**
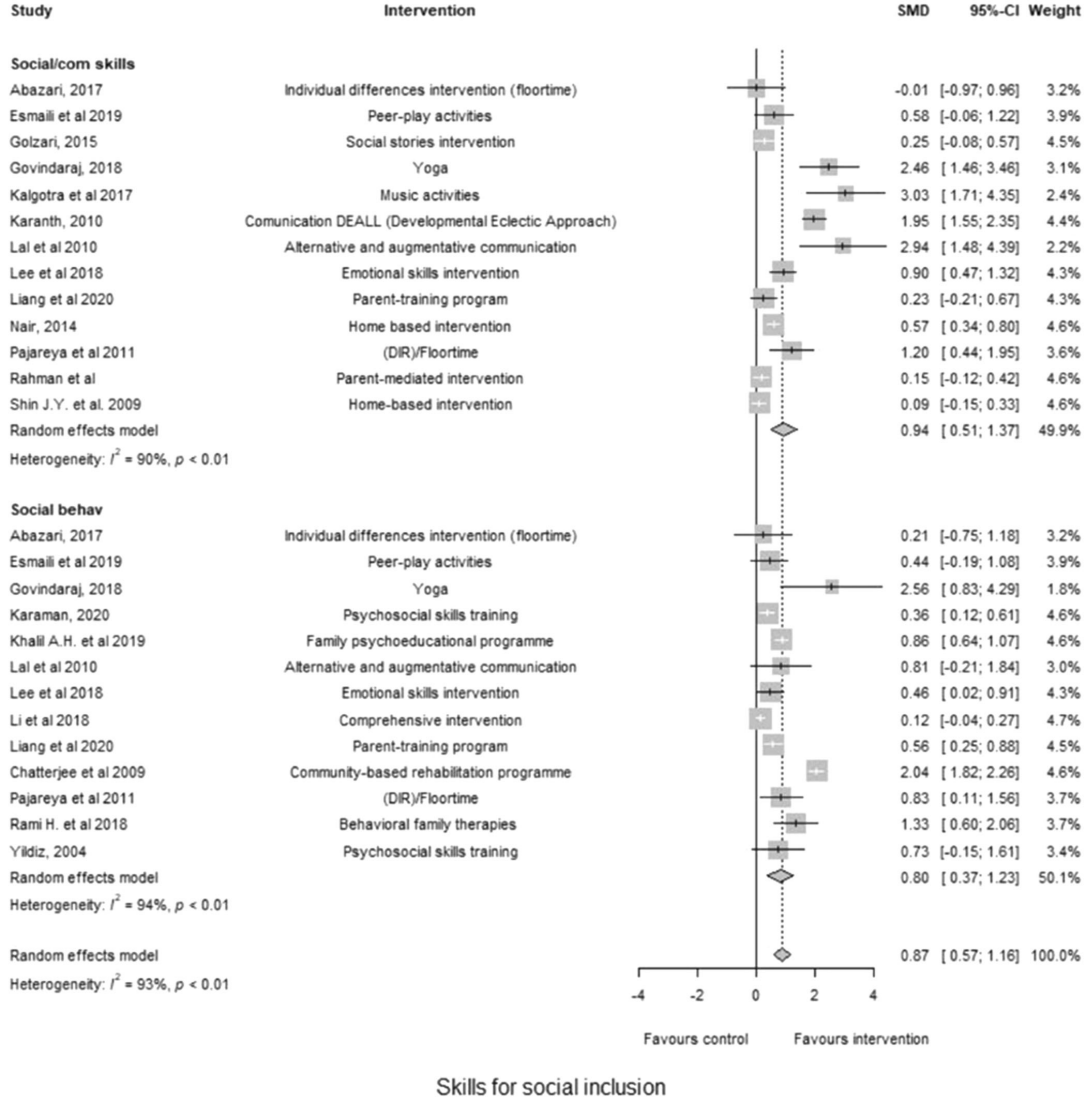
Forest plot showing the observed outcomes and the estimate of a random‐effects model on skills for social inclusion.

Of the 26 studies reported the skills for social inclusion, 14 (51.8%) reported significant improvement after intervention. A random effect model produced an overall large effect for the 26 studies (SMD = 0.87, 95% CI = 0.57 to 1.16) (Figure 4). Examination of the *I* ^2^ suggested high levels of heterogeneity (*I* ^2^ = 93%, *p* < 0.001). The influence of the intervention is also apparent for sub‐categories of this outcome, such as social behaviour (SMD = 0.94, CI = 0.50 to 1.38, *k* = 13, *I* ^2^ = 90%, *p* < 0.001) and social skills (SMD = 0.80, CI = 0.37 to 1.23, *k* = 13, *I* ^2^ = 94%, *p* < 0.001). The heterogeneity measure is high across most outcomes, as studies are dispersed in methods, study design and quality.

##### Relationship

**Figure 5:**
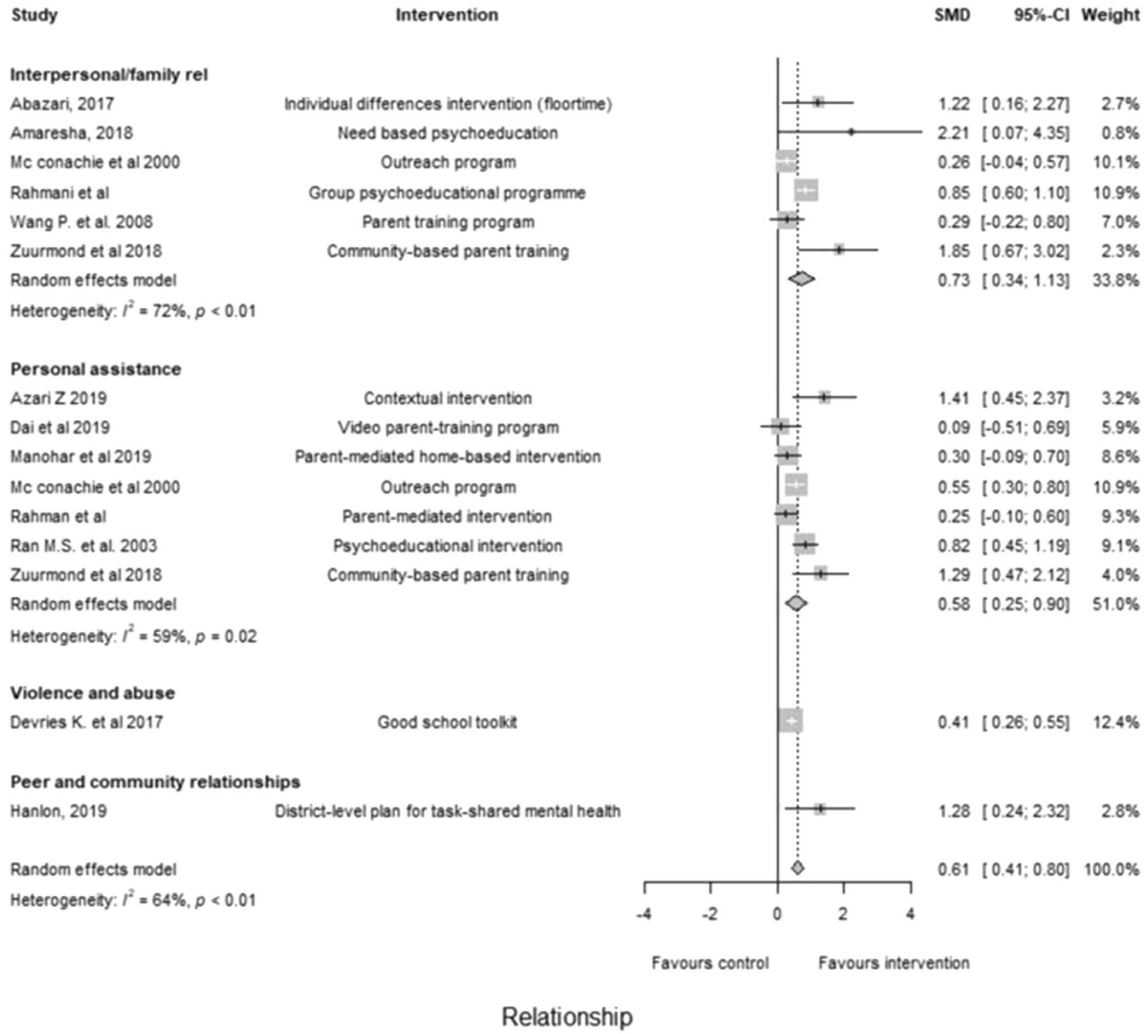
Forest plot showing the observed outcomes and the estimate of the random‐effect model on relationships.

**Figure 6:**
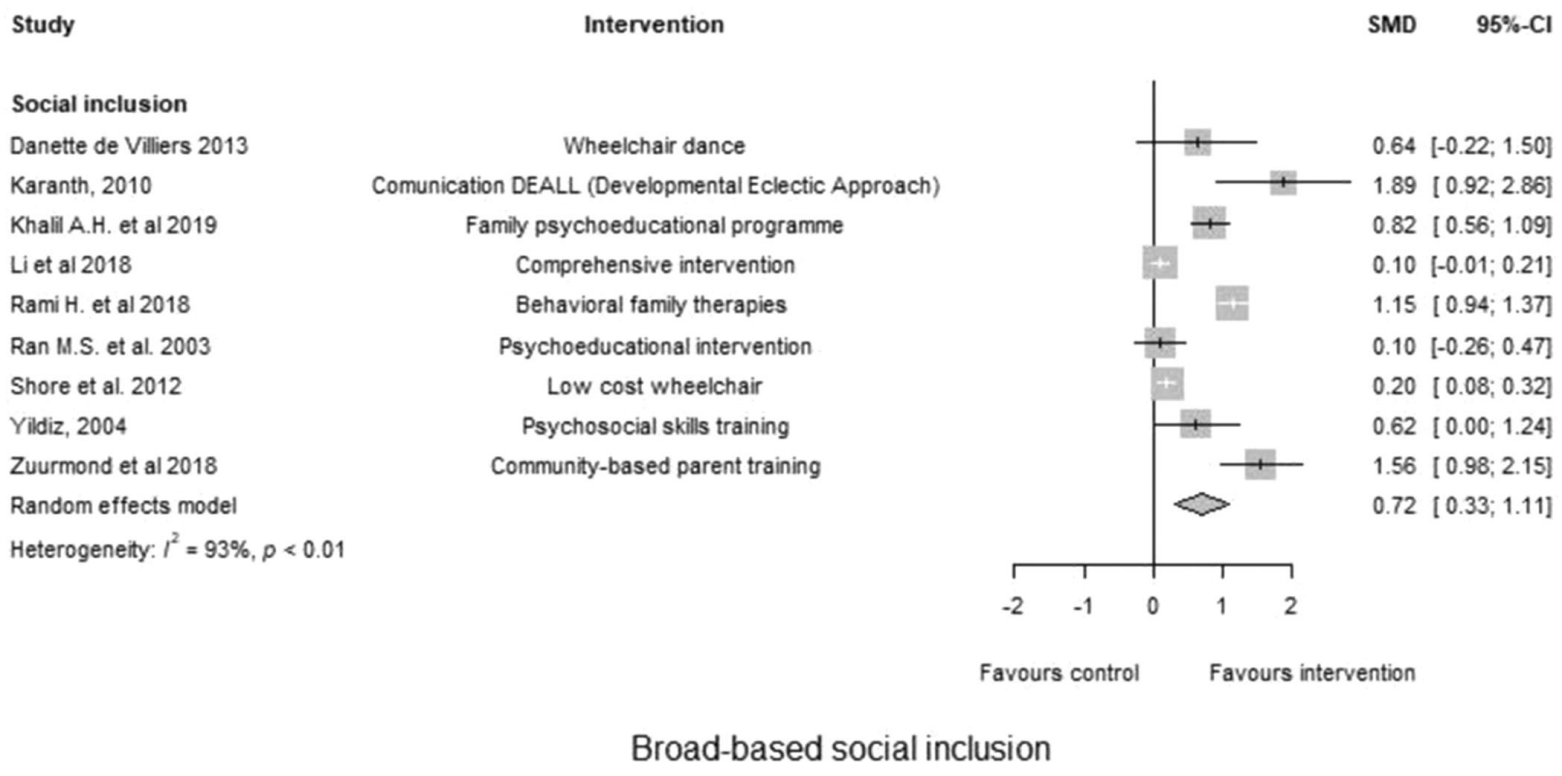
Forest plot showing the observed outcomes and the estimates of the random‐effects model on broad‐based social inclusion.

As shown in Figure 6, the forest plot illustrates the effect of interventions on improving the relationships of people with disabilities with family and community. An overall pooled effect of 15 studies showed a moderate effect with moderate heterogeneity across studies (SMD = 0.61, CI = 0.41 to 0.80, *k* = 15, *I* ^2^ = 64%, *p* = 0.01) on improving relationships between people with disabilities and their families and communities (Figure 5). Of these 15 studies, 7 articles dealt with personal assistance; 6 studies focused on family relationships, 1 investigated violence against people with disabilities and 1 on community relationships. The results of a random‐effects model based on seven studies showed a moderate overall effect following the intervention in access to personal assistance (SMD = 0.58, CI = 0.25 to 0.90, *k* = 7, *I* ^2^ = 59%, *p* = 0.02). Three out of seven studies indicate an insignificant relationship, which is relatively small compared to other output analyses. Six studies explored family relationships and found a large positive effect following intervention (SMD = 0.79, CI = 0.31 to 1.28, *k* = 6, *I* ^2^ = 72%, *p* = 0.001). A single study (Devries et al., 2018) investigated violence against people with disabilities and demonstrated a moderate effect and a significant reduction in violence (SMD = 0.41, CI = 0.26 to 0.55). Hanlon et al. (2020) also demonstrated large effects on community relations (SMD = 1.28, CI = 0.24 to 2.32).

##### Broad‐based social inclusion

Five of the nine studies have demonstrated a significant improvement in broad‐based social inclusion, including the participation of people with disabilities in music, art, recreation, and leisure. Overall, the pooled effect of nine studies showed a large effect with significant dispersion across studies (SMD = 0.72, CI = 0.33 to 1.11, *k* = 2, *I* ^2^ = 93%, *p* = 0.001) (Figure 6).

##### Sub‐group analysis

###### Social skills

**Figure 7:**
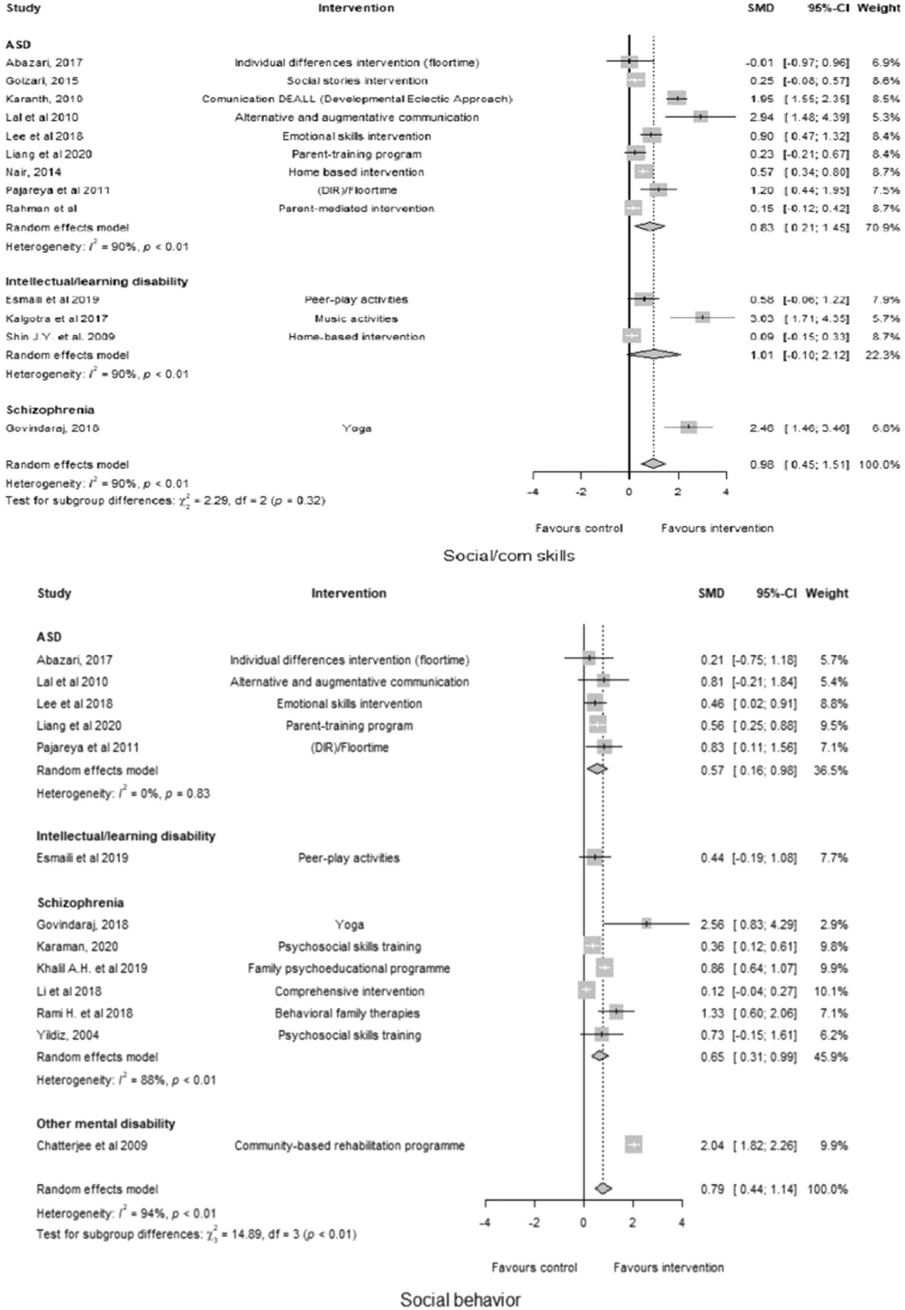
Forest plot showing the observed outcomes and the estimate of a random‐effects model on social skills and social behaviour by type of impairment.

We also analysed how the outcome differs for different types of disabilities (Figure 7). Studies on people with Autism Spectrum Disorder (ASD) reported below‐average effects on social behaviour and social skills improvement. While the effect size for social skills was large and significantly positive (SMD = 0.83, CI = 0.21 to 1.45, *k* = 9, *I* ^2^ = 90%, *p* < 0.01), the effect size for social behaviour, even though positive and significant was moderate (SMD = 0.57, CI = 0.16 to 0.98, *k* = 5, *I* ^2^ = 0%, *p* = 0.83). The estimates for social behaviour are also consistent across all the studies. It can be concluded that the intervention is effective for people with ASD and that social behaviour can potentially be influenced more than social skills.

We found similar results for people with learning or intellectual disabilities other than ASD. The study by Esmaili et al. (2019) found a larger effect size for social skills than for social behaviour, although non‐significant. While the SMD of the intervention on social behaviour based on a single study is 0.44 (CI = −0.19 to 1.08), the effect on social skills is noticeably high (SMD = 1.01, CI = −0.10 to 2.12, *k* = 3, *I*² = 90%, *p* < 0.001), even though the overall effect is not significant.

People with schizophrenia show similar patterns to those with ASD. The effect on social behaviour is medium for most studies, except Govindaraj et al., 2018 as the overall effect is significant and positive (SMD = 0.65, CI = 0.31 to 0.99, *k* = 6, *I*² = 88%, *p* < 0.001). The estimate of social skills improvement is based on a single study Govindaraj et al. (2018) and can be seen as non‐representative.

###### Relationships

**Figure 8:**
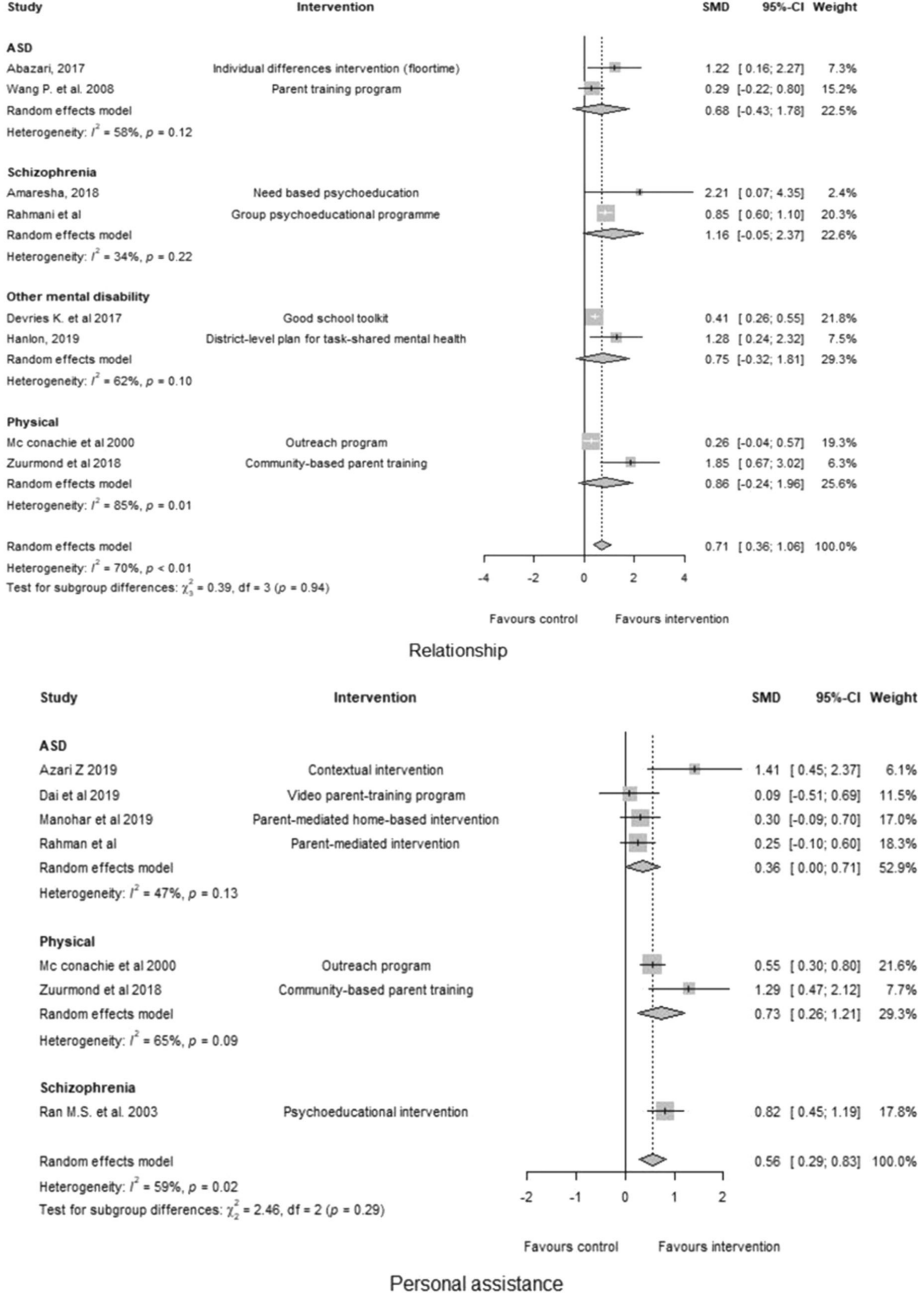
Forest plot showing the observed and the estimate of the random‐effects model on relationships and personal assistance by type of impairment.

The results for the subgroup analysis by type of impairment can be found in (Figure 8). For this analysis, we pooled the three outcome sub‐categories: interpersonal/family relationship, peer/community relationship and violence/abuse into one outcome measure due to a lack of data for the violence and community relationship (any subgroup analysis of size one would be meaningless). The sub‐group test showed no significant difference between groups, even though studies concerned people with ASD had the lowest effect size (SMD = 0.68, CI = −0.43 to 1.78, *k* = 2, *I*² = 58%, *p* = 0.12). None of the subgroups alone was significantly different from zero, mainly due to the high volatility of the effect sizes. The highest average effect size was found in studies of people with schizophrenia (SMD = 1.16, CI = −0.05 to 2.13, *k* = 2, *I* ^2^ = 34%, *p* = 0.22) followed by physical impairment (SMD = 0.86, CI = −0.24 to 1.96, *k* = 2, *I* ^2^ = 85%, *p* = 0.01) and other psychosocial impairment (SMD = 0.75, CI = −0.32 to 1.81, *k* = 2, *I* ^2^ = 62%, *p* = 0.10).

In the studies of personal assistance, the test shows no systematic difference between the groups (*χ* ^2^ = 2.46, *df* = 1, *p*= 0.29) even though the differences in effect sizes are considerable. The smallest, but also the most persistent, is the effect for people with ASD which is just significantly different from 0 (SMD = 0.36, CI = 0.00 to 0.71, *k* = 4, *I* ^2^ = 47%, *p* = 0.13). The studies concerned with people with physical impairment shows an average effect size (SMD = 0.73, CI = 0.26 to 1.21, *k* = 2, *I* ^2^ = 65%, *p* = 0.09). Similar results can be found for people with schizophrenia, even though the results are only based on a single study.

###### Broad‐based social inclusion

**Figure 9:**
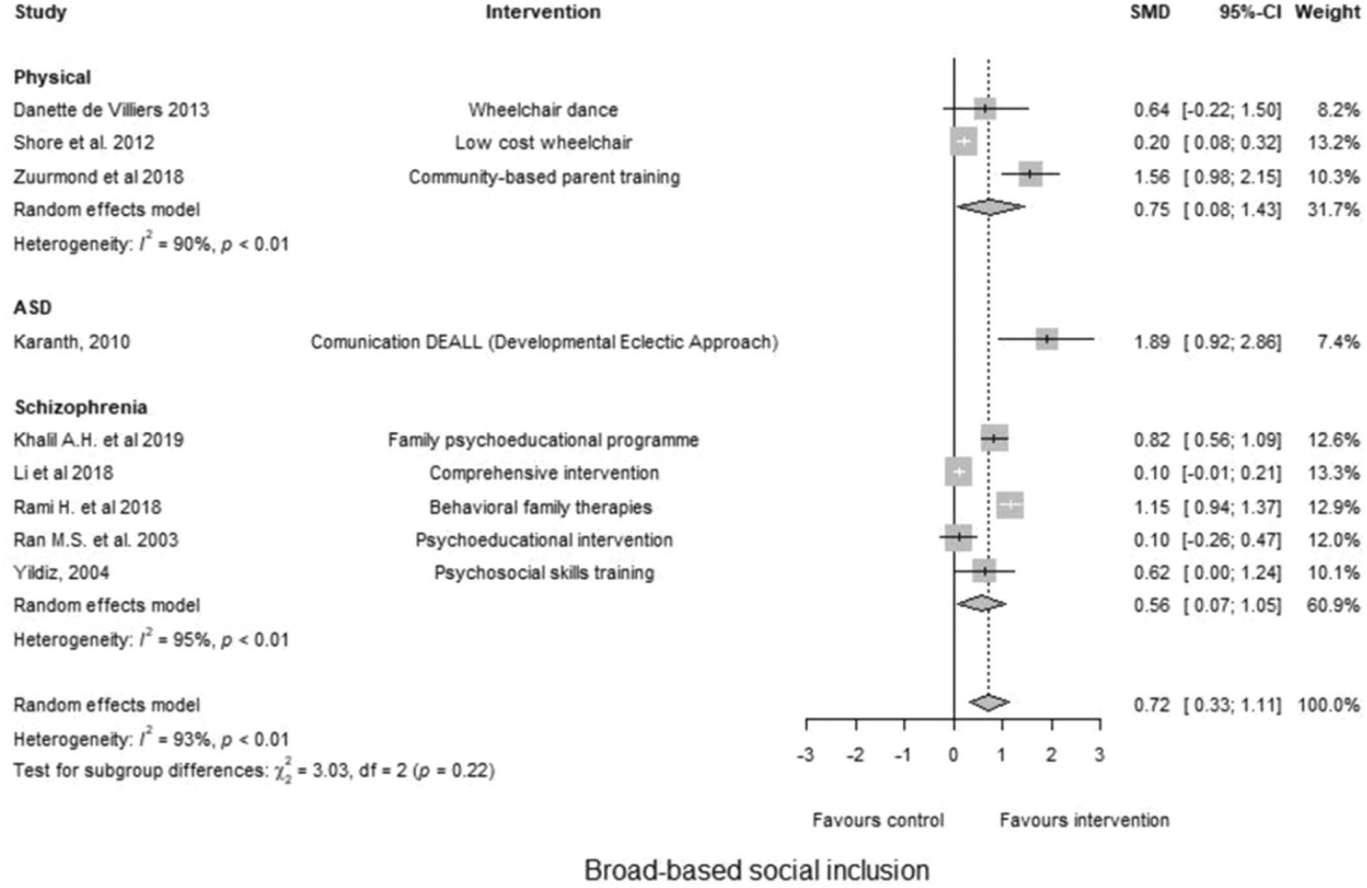
Forest plot showing the observed outcomes and the estimate of the random‐effect model on broad‐based social inclusion by type of impairment.

The overall effect for five studies on schizophrenia showed a moderate effect (SMD = 0.56, CI = 0.07 to 1.05, *k* = 2, *I*² = 95%, *p* < 0.01). Studies on people with physical impairments show a moderate effect (SMD = 0.75, CI = 0.08 to 1.43, *k* = 3, *I* ^2^ = 90%, *p* < 0.01), while the single study concerned with people with ASD reports a large effect size (SMD = 1.89, CI = 0.92 to 2.86). All the subgroups show significantly positive effect sizes (Figure 9).

###### Overall

There is promising evidence that interventions are effective at improving social and communication skills, interpersonal relationships of people with disabilities with families and broad‐based social inclusion and participation measures. However, given the low confidence (*n* = 27) in study findings related to methodological limitations, the findings must be interpreted with caution. Although there was a consensus on the direction of the effects, the studies presented considerable heterogeneity in the size of the effects. The sub‐group analysis by no means considerable, and the effect size was based on only a small number of studies.

#### 5.3.2 Publication bias

**Figure 10:**
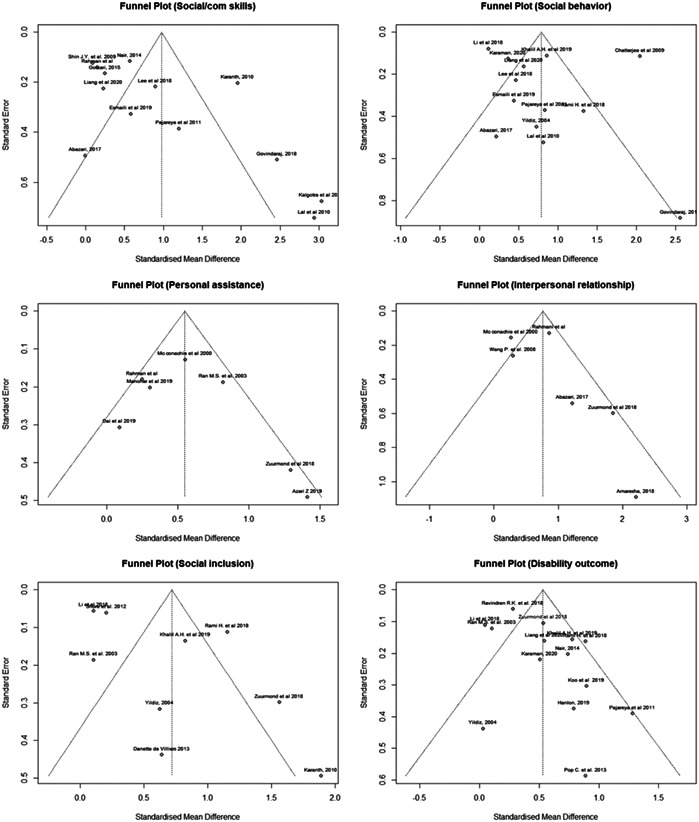
Funnel plots showing publication bias.

We examined publication bias by the funnel plot (Figure 10) and then confirmed by Eggar's test (Table 3). The funnel plot of the studies that measured social skills and broad‐based social inclusion suggests substantial publication bias. The plot is not symmetrical around the pooled effect estimate (*p* < 0.01). Most studies are outside the 95% CI that shows the expected distribution of effect sizes. This asymmetry indicates a strong publication bias, confirmed by Egger's test for social skills (3.60 (*t* = 3.48, *p* < 0.01)) and broad‐based social inclusion Egger's test 3.71 (*t* = 3.14, *p*= 0.01). In all the cases, small studies with higher standard errors systematically report higher values than studies with lower standard errors, which are seen as more precise. Even though these are unlikely to reverse the relatively robust study results, we should consider limitations when interpreting the effect sizes.

On the other hand, studies on personal assistance (Eggers test 1.37 (*t* = 1.38, *p*= 0.19)), interpersonal and family relationships (Eggers test 1.36 (*t* = 1.58, *p*= 0.15)) and social behaviour (Eggers test 1.08 (*t* = 0.75, *p*= 0.46)) shows no publication bias.

## 6 DISCUSSION

This review examines the effectiveness of interventions aimed at improving social inclusion outcomes (acquisition of skills for social inclusion, broad‐based social inclusion, and improved relationships) for people with disabilities in LMIC. We searched academic and online databases, carried out citation tracking of included studies and contacted experts to ensure our search was as comprehensive as possible. We also ran the searches with search terms specific to social inclusion review using Open Alex in EPPI reviewer (Thomas & Stansfield, 2018).

### 6.1 Summary of main results

We identified 37 experimental and quasi‐experimental studies. Most of the included studies (*n* = 13) were undertaken in South Asia, with 12 studies from India and 1 from Bangladesh. Nine studies were included from East Asia and the Pacific; the countries represented were China (9), Vietnam (2), and Thailand (1). Iran (5), Turkey (2), and Egypt (2) represented nine studies from the Middle East and North Africa. Five studies are from sub‐Saharan Africa, with studies concentrated on only a few countries representing Ethiopia, (1) Kenya (1), Ghana (1), South Africa (1) and Uganda (1). Only two studies from Europe and Central Asia represent Albania (1) and Romania (1).

There is a limited evidence base, particularly across certain geographic regions. Two World Bank regions were not widely represented in our review of Europe, Central Asia, the Middle East and North Africa. This gap may be attributable to the reporting language, as our search only covered literature in English, while many of these areas are not English‐language dominant. Further, as this review only included studies conducted in LMIC, regions with a high proportion of high‐income countries (such as Europe) may be underrepresented simply because their constituent nations are not eligible. However, it may also be the case that programming for people with disabilities in these regions is comparatively lacking. Future reviews may benefit from focusing on non‐English literature to examine this question.

Older people and service providers were underrepresented in the studies included in this review. There was also a lack of studies reporting information on the socioeconomic status of the target population. Hence, it is difficult to comment if programmes targeting very low‐income participants were represented. The studies mainly represented people with intellectual disabilities and psychosocial disabilities. This focus is possibly due to the widespread programmes on social and communication skills training that are mainly targeted at these population subgroups as they are also most at risk of stigmatisation (Bond (DDG), 2017, p. 4; Parnes et al., 2013, p. 26; Scior et al., 2015, p. 6). However, there are many opportunities for meaningful intervention with people with other impairment types.

Regarding intervention content, most (*n* = 17) of the included programmes aimed at improving the social and communication skills of people with disabilities through social skills training programmes. Ten studies focussed on providing personal assistance and support and evaluated the effects of a parent training programme on the interactive skills of parents of children with disabilities. Two studies attempted to improve community attitudes through the provision of community‐based comprehensive intervention programmes and anti‐stigma training. Only one study evaluated an intervention for preventing and reducing violence against children with disabilities (The Good School Toolkit). One included a programme that evaluated the effect of music intervention on behaviour disorders of children with intellectual disabilities, and the other evaluated impact of art therapy sessions on improving the social skills of children with ASD. Three aimed at improving the social inclusion of people with disabilities by improving access to rehabilitation, provision of wheelchairs and provision of integrated district‐level mental health care. Several categories of possible intervention were absent from the included studies, including access and participation in religious activities, access and participation in sports events, and access to justice and policy change. Most studies evaluated the effectiveness of interventions targeted at people with disabilities and their family members (e.g., improving social skills). There were no studies evaluating systems (e.g., policy) or community‐level interventions. This means the focus remains on changing individuals rather than addressing societal and community‐level disabling barriers.

A random‐effect model of 26 studies indicates an overall large and statistically significant effect for skills for social inclusion (SMD = 0.87, CI = 0.57 to 1.16). For relationships across 15 studies, we find a moderate and significant effect (SMD = 0.61, CI = 0.41 to 0.80) on improving relationships between people with disabilities and their families and communities. As for the overall effect on broad‐based social inclusion, the nine studies showed a large effect with significant dispersion across studies (SMD = 0.72, CI = 0.33 to 1.11). Although there was a consensus on the direction of the effects, the studies presented considerable heterogeneity in the size of the effects. The lack of data for certain impairment types made comparing intervention effectiveness by impairment group difficult. However, the effect of the intervention was larger and more significant for individuals with Schizophrenia (SMD = 1.16, CI = −0.05 to 2.13, *k* = 2, *I* ^2^ = 34%, *p* = 0.22) than for individuals with Autism Spectrum Disorder (SMD = 0.68, CI = −0.43 to 1.78, *k* = 2, *I* ^2^ = 58%, *p* = 0.12). Nevertheless, these effect sizes are based on a few studies and should be viewed cautiously.

Despite the significant and large effects estimated by the studies, some limitations must be noted. While moderate heterogeneity may be found in all the studies, social skills (*I* = 94%), social behaviour (*I* = 90%) and social inclusion (*I* = 93%) showed very high heterogeneity. The body of evidence used for the analysis was relatively small for outcomes of personal assistance (*k* = 7), social inclusion (*k* = 9), interpersonal relationships (*k* = 6), violence (*k* = 1) and peer relationships (*k* = 1). Evidence also suggested the presence of publication bias, particularly related to social skills (*p* < 0.01) and social inclusion (*p* = 0.01), are all likely to be inflated by the existence of the publication bias. Also, given the low confidence (*n* = 27) in study findings related to methodological limitations, the findings must be interpreted with caution.

Overall, the review's findings suggest that the Interventions such as social and communication training and personal assistance led to significant improvement in the social behaviour and social skills of people with disabilities. Studies targeting relationships in families and communities showed a large and significant positive effect. However, the available evidence focused primarily on individual‐level barriers, such as interventions for improving the social or communications skills of people with disabilities and not the systemic drivers of exclusions, such as addressing societal barriers to inclusion, such as stigma reduction, and interventions to strengthen legislation, infrastructure, and institutions.

### 6.2 Overall completeness and applicability of evidence

The evidence presented here provides emerging support for the efficacy and effectiveness of interventions to improve the social inclusion of people with disabilities in LMIC due to the broad variety of interventions and outcomes assessed under the domain of social inclusion. Our review covers evidence from 17 countries, but the geographical distribution of studies is uneven. We identified a large number of studies from South Asia. However, the low number of studies from the Middle East and North Africa, sub‐Saharan Africa and no studies from Latin America and Central Asia indicates that the need for evidence is especially acute for these regions.

An important gap is the lack of studies addressing community and societal‐level barriers as outcomes or interventions, so that interventions were generally individual targeted. Moreover, only two studies were identified that addressed stigma reduction, albeit these showed improved attitudes to the person with disabilities due to the intervention. This gap is a critical omission, as stigmatising attitudes and norms are major barriers to the social inclusion of people with disabilities and their empowerment.

Disability is a highly heterogeneous category, including people with a broad range of impairments who will face different challenges and facilitators to social inclusion and empowerment. Most studies focused on people with intellectual disabilities and psychosocial impairments. Hence, it was impossible to compare the intervention's effectiveness for people with different or multiple impairments.

People with disabilities experience exclusion and disempowerment in diverse ways depending on their impairment type, gender, ethnicity, and other characteristics and contexts. The studies failed to disaggregate by gender, limiting our ability to discern whether interventions were equally effective for both genders or to explore the intersectionality between disability and other characteristics associated with discrimination, such as age and ethnicity. Studies are needed that assess interventions for a broader range of impairment types, for both genders, in humanitarian contexts and allow disaggregation of effects.

### 6.3 Quality of the evidence

The strength of the review is that we have included studies that used randomised or other rigorous quasi‐experimental study designs to answer our review questions. About 48% of the included studies used an RCT design with randomly allocated treatments to individuals or clusters. Of these, 27% (*n* = 5) were assessed to be of high‐ confidence by the confidence in the study findings tool. Overall, 72% of the studies were assessed to be of low quality and have methodological limitations. The quality of the included studies is therefore generally low For example, losses to follow‐up and other vital dimensions of study rigour were frequently either poorly recorded or poorly reported.

### 6.4 Potential biases in the review process

Studies were only eligible for inclusion if published after 2000 and in English. Our restricted eligibility criteria, requiring that primary studies were impact evaluations and conducted in an LMIC, meant that some potentially informative studies were excluded. This included non‐intervention studies conducted in LMICs (e.g., qualitative studies, process evaluations), interventions of people from LMIC communities living in high‐income settings, or interventions from high‐income settings. Bias could also have been introduced where the research team had different ideas about relevant interventions and outcomes or understandings of disability programming. To address this potential source of bias, all full‐text reviews and coding decisions were made by at least two researchers on the team, coming to a consensus on whether an article should be included and how relevant information should be extracted.

### 6.5 Agreements and disagreements with other studies or reviews

Evidence within the existing systematic reviews is consistent with our finding that interventions were effective at improving social inclusion of people with disabilities For instance, Velema et al. (2008) assessed the evidence for the effectiveness of rehabilitation‐in‐the‐community programmes. They concluded that CBR activities result in social processes that change how community members view persons with disabilities, increase their acceptance and social inclusion, and mobilise resources to meet their needs. However, the individual studies included in the review did not focus on improving social inclusion. Almerie et al. (2015) reviewed social skills programmes for people with schizophrenia and identified 13 RCTs. They concluded that social skills training may be effective at improving the social skills of people with schizophrenia but that the data is limited and generally of low quality in accordance with the findings of our review. Mikton et al. (2014) conducted a systematic review of the effectiveness of interventions to prevent and respond to violence against persons with disabilities and identified 10 eligible studies, of which only one was from an LMIC.

Overall, the broad range of interventions and outcomes employed makes comparison difficult. For instance, Hartling et al. (2014) conducted a systematic review of interventions to support siblings of children with chronic illness or disability. They identified 14 eligible studies but concluded that ‘Study differences made it difficult to determine which sibling care features were most salient.’ Similarly, Iemmi et al. (2015) undertook a systematic review of CBR for people with disabilities in LMICs. They identified only 15 eligible studies, primarily focused on health‐related interventions, used a wide variety of interventions and outcomes, and were mostly of low quality.

## 7 AUTHORS’ CONCLUSIONS

### 7.1 Implications for practice

Our review finds promising evidence that a range of interventions can effectively improve the social skills and relationships of people with disabilities. There is also evidence from one intervention that effectively prevented violence perpetrated by teachers and peers against children with disabilities. Therefore, The current evidence base supports programmes for people with disabilities to assist in relationship and social skills development programmes. However, beyond that, no implications for policy or practice can be identified from the review, as the evidence base was limited to effectiveness analyses.

The review highlighted the gap in systems‐level interventions. Interventions at the structural level, including legislation to tackle stigma and discrimination, documenting violence against people with disabilities and advocacy interventions, are essential for tackling stigma. Legislation, policies, and strategies that comply with the CRPD must be implemented and monitored to support the social inclusion of people with disabilities. Undertaking in‐country analyses of whether social inclusion policies and national and international legislation are in place are needed. Research evaluating programmes for people with disabilities other than intellectual disability and psychosocial impairments are lacking and are needed. Inclusive decision‐making may ensure the active participation of people with disabilities in interpreting evidence on interventions for people with disabilities to inform policy and practice. Advocacy efforts are needed to encourage funders (including governments, multilateral agencies and research institutes) to commit financial support to these studies. The evidence base on stigma reduction interventions for people with disabilities is weak. There is an urgent need for a holistic research approach to stigma reduction aimed at changing behaviour rather than raising awareness.

### 7.2 Implications for research

The evidence base on ‘what works’ is limited to only a few countries and focuses primarily on improving the individual‐level social and communication skills and maintaining family relationships of people with disabilities. More studies are needed to assess the impact of interventions on access to justice, peer and community relationships and community integration. The available evidence focused primarily on individual‐level barriers, such as interventions for improving the social or communications skills of people with disabilities and not the systemic drivers of exclusions, such as addressing societal barriers to inclusion, such as stigma reduction, and interventions to strengthen legislation, infrastructure, and institutions. Evidence is, thus, required to evaluate the effectiveness of interventions targeted at the system (e.g., policy) or community level (e.g., stigma reduction) rather than at people with disabilities and their family members (e.g., improving social skills).

More studies are needed that include people with a broader range of impairment types and from different settings, including humanitarian settings. They must disaggregate results by impairment type, gender, age, ethnicity, and other equity characteristics. Generally, methodological details are reported poorly; transparent reporting (e.g., standard deviations and sample sizes for treatment and control groups) would help support the inclusion of existing research that may have been missed. There is a lack of evidence on several categories of possible intervention, including access and participation in religious activities, access and participation in sports events, access to justice and policy change. There were additional gaps in equity with a lack of studies undertaken in a humanitarian context, and data were lacking on whether outcomes differed according to gender or sexuality or LGBTQ+ community or whether interventions were cost‐effective. Hence, studies undertaken should consistently consider a broad range of characteristics and aspects of identity (e.g., gender, ethnicity, and intersectionality), which may influence outcomes.

## ACKNOWLEDGEMENTS

We would like to acknowledge UK Department of International Development (DFID) under its support for the Centre for Excellence for Development Impact and Learning (CEDIL) and the Programme for Evidence to iNform Disability Action (PENDA) for the funding support.

## CONTRIBUTIONS OF AUTHORS

- Content expertise: Professor Hannah Kuper, Director of the International Centre for Evidence in Disability, a research group at LSHTM that works to expand the research and teaching activities of LSHTM in the field of global disability. Her main research interest is disability in low and middle income countries, with a particular focus on assessment of the prevalence of disability and impairments, including in children, and development of new methods in undertaking these surveys (e.g., use of mobile technologies), investigation of the health and rehabilitation needs of people with disabilities, and how these can be met in low resources settings and research on the relationship between poverty and disability, and the potential role of social protection in breaking this cycle. She has an undergraduate degree from Oxford University in Human Sciences and a doctorate from Harvard University in epidemiology. She has worked at LSHTM since 2002.
- Systematic review method and statistical analysis expertise: All team members have previous experience in systematic review methodology, including search, data collection, statistical analysis, theory‐based synthesis, which mean they are proficient in carrying out the various processes in a systematic review, such as search, eligibility screening, quality assessment and coding. Furthermore, all three authors have experience in statistical analysis of data generated through a systematic review.
- Information retrieval expertise: All authors have previous experience in developing search strategies.

## DECLARATIONS OF INTEREST

The authors have no interests to declare.

Abazari et al., 2017

**Table:** 

**Methods**
**Participants**
**Interventions**
**Outcomes**
**Notes**

Risk of bias table

**Table:** 

Bias	Authors’ judgement	Support for judgement
Random sequence generation (selection bias)	Unclear risk	
Allocation concealment (selection bias)	Unclear risk	
Blinding of participants and personnel (performance bias)	Unclear risk	
Blinding of outcome assessment (detection bias)	Unclear risk	
Incomplete outcome data (attrition bias)	Unclear risk	
Selective reporting (reporting bias)	Unclear risk	
Other bias	Unclear risk	

Amaresha et al., 2018

**Table:** 

**Methods**
**Participants**
**Interventions**
**Outcomes**
**Notes**

Risk of bias table

**Table:** 

Bias	Authors’ judgement	Support for judgement
Random sequence generation (selection bias)	Unclear risk	
Allocation concealment (selection bias)	Unclear risk	
Blinding of participants and personnel (performance bias)	Unclear risk	
Blinding of outcome assessment (detection bias)	Unclear risk	
Incomplete outcome data (attrition bias)	Unclear risk	
Selective reporting (reporting bias)	Unclear risk	
Other bias	Unclear risk	

Azari et al., 2019

**Table:** 

**Methods**
**Participants**
**Interventions**
**Outcomes**
**Notes**

Risk of bias table

**Table:** 

Bias	Authors’ judgement	Support for judgement
Random sequence generation (selection bias)	Unclear risk	
Allocation concealment (selection bias)	Unclear risk	
Blinding of participants and personnel (performance bias)	Unclear risk	
Blinding of outcome assessment (detection bias)	Unclear risk	
Incomplete outcome data (attrition bias)	Unclear risk	
Selective reporting (reporting bias)	Unclear risk	
Other bias	Unclear risk	

Dai et al., 2018

**Table:** 

**Methods**
**Participants**
**Interventions**
**Outcomes**
**Notes**

Risk of bias table

**Table:** 

Bias	Authors’ judgement	Support for judgement
Random sequence generation (selection bias)	Unclear risk	
Allocation concealment (selection bias)	Unclear risk	
Blinding of participants and personnel (performance bias)	Unclear risk	
Blinding of outcome assessment (detection bias)	Unclear risk	
Incomplete outcome data (attrition bias)	Unclear risk	
Selective reporting (reporting bias)	Unclear risk	
Other bias	Unclear risk	

De Villiers et al., 2013

**Table:** 

**Methods**
**Participants**
**Interventions**
**Outcomes**
**Notes**

Risk of bias table

**Table:** 

Bias	Authors’ judgement	Support for judgement
Random sequence generation (selection bias)	Unclear risk	
Allocation concealment (selection bias)	Unclear risk	
Blinding of participants and personnel (performance bias)	Unclear risk	
Blinding of outcome assessment (detection bias)	Unclear risk	
Incomplete outcome data (attrition bias)	Unclear risk	
Selective reporting (reporting bias)	Unclear risk	
Other bias	Unclear risk	

Devries et al., 2018

**Table:** 

**Methods**
**Participants**
**Interventions**
**Outcomes**
**Notes**

Risk of bias table

**Table:** 

Bias	Authors’ judgement	Support for judgement
Random sequence generation (selection bias)	Unclear risk	
Allocation concealment (selection bias)	Unclear risk	
Blinding of participants and personnel (performance bias)	Unclear risk	
Blinding of outcome assessment (detection bias)	Unclear risk	
Incomplete outcome data (attrition bias)	Unclear risk	
Selective reporting (reporting bias)	Unclear risk	
Other bias	Unclear risk	

Esmaili et al., 2019

**Table:** 

**Methods**
**Participants**
**Interventions**
**Outcomes**
**Notes**

Risk of bias table

**Table:** 

Bias	Authors’ judgement	Support for judgement
Random sequence generation (selection bias)	Unclear risk	
Allocation concealment (selection bias)	Unclear risk	
Blinding of participants and personnel (performance bias)	Unclear risk	
Blinding of outcome assessment (detection bias)	Unclear risk	
Incomplete outcome data (attrition bias)	Unclear risk	
Selective reporting (reporting bias)	Unclear risk	
Other bias	Unclear risk	

Golzari et al., 2015

**Table:** 

**Methods**
**Participants**
**Interventions**
**Outcomes**
**Notes**

Risk of bias table

**Table:** 

Bias	Authors’ judgement	Support for judgement
Random sequence generation (selection bias)	Unclear risk	
Allocation concealment (selection bias)	Unclear risk	
Blinding of participants and personnel (performance bias)	Unclear risk	
Blinding of outcome assessment (detection bias)	Unclear risk	
Incomplete outcome data (attrition bias)	Unclear risk	
Selective reporting (reporting bias)	Unclear risk	
Other bias	Unclear risk	

Govindaraj et al., 2018

**Table:** 

**Methods**
**Participants**
**Interventions**
**Outcomes**
**Notes**

Risk of bias table

**Table:** 

Bias	Authors’ judgement	Support for judgement
Random sequence generation (selection bias)	Unclear risk	
Allocation concealment (selection bias)	Unclear risk	
Blinding of participants and personnel (performance bias)	Unclear risk	
Blinding of outcome assessment (detection bias)	Unclear risk	
Incomplete outcome data (attrition bias)	Unclear risk	
Selective reporting (reporting bias)	Unclear risk	
Other bias	Unclear risk	

Hanlon et al., 2020

**Table:** 

**Methods**
**Participants**
**Interventions**
**Outcomes**
**Notes**

Risk of bias table

**Table:** 

Bias	Authors’ judgement	Support for judgement
Random sequence generation (selection bias)	Unclear risk	
Allocation concealment (selection bias)	Unclear risk	
Blinding of participants and personnel (performance bias)	Unclear risk	
Blinding of outcome assessment (detection bias)	Unclear risk	
Incomplete outcome data (attrition bias)	Unclear risk	
Selective reporting (reporting bias)	Unclear risk	
Other bias	Unclear risk	

Juneja et al., 2012

**Table:** 

**Methods**
**Participants**
**Interventions**
**Outcomes**
**Notes**

Risk of bias table

**Table:** 

Bias	Authors’ judgement	Support for judgement
Random sequence generation (selection bias)	Unclear risk	
Allocation concealment (selection bias)	Unclear risk	
Blinding of participants and personnel (performance bias)	Unclear risk	
Blinding of outcome assessment (detection bias)	Unclear risk	
Incomplete outcome data (attrition bias)	Unclear risk	
Selective reporting (reporting bias)	Unclear risk	
Other bias	Unclear risk	

Kalgotra & Warwal, 2017

**Table:** 

**Methods**
**Participants**
**Interventions**
**Outcomes**
**Notes**

Risk of bias table

**Table:** 

Bias	Authors’ judgement	Support for judgement
Random sequence generation (selection bias)	Unclear risk	
Allocation concealment (selection bias)	Unclear risk	
Blinding of participants and personnel (performance bias)	Unclear risk	
Blinding of outcome assessment (detection bias)	Unclear risk	
Incomplete outcome data (attrition bias)	Unclear risk	
Selective reporting (reporting bias)	Unclear risk	
Other bias	Unclear risk	

Karaman et al., 2020

**Table:** 

**Methods**
**Participants**
**Interventions**
**Outcomes**
**Notes**

Risk of bias table

**Table:** 

Bias	Authors’ judgement	Support for judgement
Random sequence generation (selection bias)	Unclear risk	
Allocation concealment (selection bias)	Unclear risk	
Blinding of participants and personnel (performance bias)	Unclear risk	
Blinding of outcome assessment (detection bias)	Unclear risk	
Incomplete outcome data (attrition bias)	Unclear risk	
Selective reporting (reporting bias)	Unclear risk	
Other bias	Unclear risk	

Karanth et al., 2010

**Table:** 

**Methods**
**Participants**
**Interventions**
**Outcomes**
**Notes**

Risk of bias table

**Table:** 

Bias	Authors’ judgement	Support for judgement
Random sequence generation (selection bias)	Unclear risk	
Allocation concealment (selection bias)	Unclear risk	
Blinding of participants and personnel (performance bias)	Unclear risk	
Blinding of outcome assessment (detection bias)	Unclear risk	
Incomplete outcome data (attrition bias)	Unclear risk	
Selective reporting (reporting bias)	Unclear risk	
Other bias	Unclear risk	

Khalil et al., 2019

**Table:** 

**Methods**
**Participants**
**Interventions**
**Outcomes**
**Notes**

Risk of bias table

**Table:** 

Bias	Authors’ judgement	Support for judgement
Random sequence generation (selection bias)	Unclear risk	
Allocation concealment (selection bias)	Unclear risk	
Blinding of participants and personnel (performance bias)	Unclear risk	
Blinding of outcome assessment (detection bias)	Unclear risk	
Incomplete outcome data (attrition bias)	Unclear risk	
Selective reporting (reporting bias)	Unclear risk	
Other bias	Unclear risk	

Koo & Thomas, 2019

**Table:** 

**Methods**
**Participants**
**Interventions**
**Outcomes**
**Notes**

Risk of bias table

**Table:** 

Bias	Authors’ judgement	Support for judgement
Random sequence generation (selection bias)	Unclear risk	
Allocation concealment (selection bias)	Unclear risk	
Blinding of participants and personnel (performance bias)	Unclear risk	
Blinding of outcome assessment (detection bias)	Unclear risk	
Incomplete outcome data (attrition bias)	Unclear risk	
Selective reporting (reporting bias)	Unclear risk	
Other bias	Unclear risk	

Lal, 2010

**Table:** 

**Methods**
**Participants**
**Interventions**
**Outcomes**
**Notes**

Risk of bias table

**Table:** 

Bias	Authors’ judgement	Support for judgement
Random sequence generation (selection bias)	Unclear risk	
Allocation concealment (selection bias)	Unclear risk	
Blinding of participants and personnel (performance bias)	Unclear risk	
Blinding of outcome assessment (detection bias)	Unclear risk	
Incomplete outcome data (attrition bias)	Unclear risk	
Selective reporting (reporting bias)	Unclear risk	
Other bias	Unclear risk	

Lee et al., 2019

**Table:** 

**Methods**
**Participants**
**Interventions**
**Outcomes**
**Notes**

Risk of bias table

**Table:** 

Bias	Authors’ judgement	Support for judgement
Random sequence generation (selection bias)	Unclear risk	
Allocation concealment (selection bias)	Unclear risk	
Blinding of participants and personnel (performance bias)	Unclear risk	
Blinding of outcome assessment (detection bias)	Unclear risk	
Incomplete outcome data (attrition bias)	Unclear risk	
Selective reporting (reporting bias)	Unclear risk	
Other bias	Unclear risk	

Li et al., 2018

**Table:** 

**Methods**
**Participants**
**Interventions**
**Outcomes**
**Notes**

Risk of bias table

**Table:** 

Bias	Authors’ judgement	Support for judgement
Random sequence generation (selection bias)	Unclear risk	
Allocation concealment (selection bias)	Unclear risk	
Blinding of participants and personnel (performance bias)	Unclear risk	
Blinding of outcome assessment (detection bias)	Unclear risk	
Incomplete outcome data (attrition bias)	Unclear risk	
Selective reporting (reporting bias)	Unclear risk	
Other bias	Unclear risk	

Li et al., 2019

**Table:** 

**Methods**
**Participants**
**Interventions**
**Outcomes**
**Notes**

Risk of bias table

**Table:** 

Bias	Authors’ judgement	Support for judgement
Random sequence generation (selection bias)	Unclear risk	
Allocation concealment (selection bias)	Unclear risk	
Blinding of participants and personnel (performance bias)	Unclear risk	
Blinding of outcome assessment (detection bias)	Unclear risk	
Incomplete outcome data (attrition bias)	Unclear risk	
Selective reporting (reporting bias)	Unclear risk	
Other bias	Unclear risk	

Liang et al., 2022

**Table:** 

**Methods**
**Participants**
**Interventions**
**Outcomes**
**Notes**

Risk of bias table

**Table:** 

Bias	Authors’ judgement	Support for judgement
Random sequence generation (selection bias)	Unclear risk	
Allocation concealment (selection bias)	Unclear risk	
Blinding of participants and personnel (performance bias)	Unclear risk	
Blinding of outcome assessment (detection bias)	Unclear risk	
Incomplete outcome data (attrition bias)	Unclear risk	
Selective reporting (reporting bias)	Unclear risk	
Other bias	Unclear risk	

Lund et al., 2013

**Table:** 

**Methods**
**Participants**
**Interventions**
**Outcomes**
**Notes**

Risk of bias table

**Table:** 

Bias	Authors’ judgement	Support for judgement
Random sequence generation (selection bias)	Unclear risk	
Allocation concealment (selection bias)	Unclear risk	
Blinding of participants and personnel (performance bias)	Unclear risk	
Blinding of outcome assessment (detection bias)	Unclear risk	
Incomplete outcome data (attrition bias)	Unclear risk	
Selective reporting (reporting bias)	Unclear risk	
Other bias	Unclear risk	

Manohar et al., 2019

**Table:** 

**Methods**
**Participants**
**Interventions**
**Outcomes**
**Notes**

Risk of bias table

**Table:** 

Bias	Authors’ judgement	Support for judgement
Random sequence generation (selection bias)	Unclear risk	
Allocation concealment (selection bias)	Unclear risk	
Blinding of participants and personnel (performance bias)	Unclear risk	
Blinding of outcome assessment (detection bias)	Unclear risk	
Incomplete outcome data (attrition bias)	Unclear risk	
Selective reporting (reporting bias)	Unclear risk	
Other bias	Unclear risk	

McConachie et al., 2000

**Table:** 

**Methods**
**Participants**
**Interventions**
**Outcomes**
**Notes**

Risk of bias table

**Table:** 

Bias	Authors’ judgement	Support for judgement
Random sequence generation (selection bias)	Unclear risk	
Allocation concealment (selection bias)	Unclear risk	
Blinding of participants and personnel (performance bias)	Unclear risk	
Blinding of outcome assessment (detection bias)	Unclear risk	
Incomplete outcome data (attrition bias)	Unclear risk	
Selective reporting (reporting bias)	Unclear risk	
Other bias	Unclear risk	

Nair et al., 2014

**Table:** 

**Methods**
**Participants**
**Interventions**
**Outcomes**
**Notes**

Risk of bias table

**Table:** 

Bias	Authors’ judgement	Support for judgement
Random sequence generation (selection bias)	Unclear risk	
Allocation concealment (selection bias)	Unclear risk	
Blinding of participants and personnel (performance bias)	Unclear risk	
Blinding of outcome assessment (detection bias)	Unclear risk	
Incomplete outcome data (attrition bias)	Unclear risk	
Selective reporting (reporting bias)	Unclear risk	
Other bias	Unclear risk	

Pajareya & Nopmaneejumruslers, 2011

**Table:** 

**Methods**
**Participants**
**Interventions**
**Outcomes**
**Notes**

Risk of bias table

**Table:** 

Bias	Authors’ judgement	Support for judgement
Random sequence generation (selection bias)	Unclear risk	
Allocation concealment (selection bias)	Unclear risk	
Blinding of participants and personnel (performance bias)	Unclear risk	
Blinding of outcome assessment (detection bias)	Unclear risk	
Incomplete outcome data (attrition bias)	Unclear risk	
Selective reporting (reporting bias)	Unclear risk	
Other bias	Unclear risk	

Pop et al., 2013

**Table:** 

**Methods**
**Participants**
**Interventions**
**Outcomes**
**Notes**

Risk of bias table

**Table:** 

Bias	Authors’ judgement	Support for judgement
Random sequence generation (selection bias)	Unclear risk	
Allocation concealment (selection bias)	Unclear risk	
Blinding of participants and personnel (performance bias)	Unclear risk	
Blinding of outcome assessment (detection bias)	Unclear risk	
Incomplete outcome data (attrition bias)	Unclear risk	
Selective reporting (reporting bias)	Unclear risk	
Other bias	Unclear risk	

Rahman et al., 2016

**Table:** 

**Methods**
**Participants**
**Interventions**
**Outcomes**
**Notes**

Risk of bias table

**Table:** 

Bias	Authors’ judgement	Support for judgement
Random sequence generation (selection bias)	Unclear risk	
Allocation concealment (selection bias)	Unclear risk	
Blinding of participants and personnel (performance bias)	Unclear risk	
Blinding of outcome assessment (detection bias)	Unclear risk	
Incomplete outcome data (attrition bias)	Unclear risk	
Selective reporting (reporting bias)	Unclear risk	
Other bias	Unclear risk	

Rahmani et al., 2015

**Table:** 

**Methods**
**Participants**
**Interventions**
**Outcomes**
**Notes**

Risk of bias table

**Table:** 

Bias	Authors’ judgement	Support for judgement
Random sequence generation (selection bias)	Unclear risk	
Allocation concealment (selection bias)	Unclear risk	
Blinding of participants and personnel (performance bias)	Unclear risk	
Blinding of outcome assessment (detection bias)	Unclear risk	
Incomplete outcome data (attrition bias)	Unclear risk	
Selective reporting (reporting bias)	Unclear risk	
Other bias	Unclear risk	

Rami et al., 2018

**Table:** 

**Methods**
**Participants**
**Interventions**
**Outcomes**
**Notes**

Risk of bias table

**Table:** 

Bias	Authors’ judgement	Support for judgement
Random sequence generation (selection bias)	Unclear risk	
Allocation concealment (selection bias)	Unclear risk	
Blinding of participants and personnel (performance bias)	Unclear risk	
Blinding of outcome assessment (detection bias)	Unclear risk	
Incomplete outcome data (attrition bias)	Unclear risk	
Selective reporting (reporting bias)	Unclear risk	
Other bias	Unclear risk	

Ran et al., 2003

**Table:** 

**Methods**
**Participants**
**Interventions**
**Outcomes**
**Notes**

Risk of bias table

**Table:** 

Bias	Authors’ judgement	Support for judgement
Random sequence generation (selection bias)	Unclear risk	
Allocation concealment (selection bias)	Unclear risk	
Blinding of participants and personnel (performance bias)	Unclear risk	
Blinding of outcome assessment (detection bias)	Unclear risk	
Incomplete outcome data (attrition bias)	Unclear risk	
Selective reporting (reporting bias)	Unclear risk	
Other bias	Unclear risk	

Ravindren et al., 2018

**Table:** 

**Methods**
**Participants**
**Interventions**
**Outcomes**
**Notes**

Risk of bias table

**Table:** 

Bias	Authors’ judgement	Support for judgement
Random sequence generation (selection bias)	Unclear risk	
Allocation concealment (selection bias)	Unclear risk	
Blinding of participants and personnel (performance bias)	Unclear risk	
Blinding of outcome assessment (detection bias)	Unclear risk	
Incomplete outcome data (attrition bias)	Unclear risk	
Selective reporting (reporting bias)	Unclear risk	
Other bias	Unclear risk	

Shin et al., 2009

**Table:** 

**Methods**
**Participants**
**Interventions**
**Outcomes**
**Notes**

Risk of bias table

**Table:** 

Bias	Authors’ judgement	Support for judgement
Random sequence generation (selection bias)	Unclear risk	
Allocation concealment (selection bias)	Unclear risk	
Blinding of participants and personnel (performance bias)	Unclear risk	
Blinding of outcome assessment (detection bias)	Unclear risk	
Incomplete outcome data (attrition bias)	Unclear risk	
Selective reporting (reporting bias)	Unclear risk	
Other bias	Unclear risk	

Shore & Juillerat, 2012

**Table:** 

**Methods**
**Participants**
**Interventions**
**Outcomes**
**Notes**

Risk of bias table

**Table:** 

Bias	Authors’ judgement	Support for judgement
Random sequence generation (selection bias)	Unclear risk	
Allocation concealment (selection bias)	Unclear risk	
Blinding of participants and personnel (performance bias)	Unclear risk	
Blinding of outcome assessment (detection bias)	Unclear risk	
Incomplete outcome data (attrition bias)	Unclear risk	
Selective reporting (reporting bias)	Unclear risk	
Other bias	Unclear risk	

Wang, 2008

**Table:** 

**Methods**
**Participants**
**Interventions**
**Outcomes**
**Notes**

Risk of bias table

**Table:** 

Bias	Authors’ judgement	Support for judgement
Random sequence generation (selection bias)	Unclear risk	
Allocation concealment (selection bias)	Unclear risk	
Blinding of participants and personnel (performance bias)	Unclear risk	
Blinding of outcome assessment (detection bias)	Unclear risk	
Incomplete outcome data (attrition bias)	Unclear risk	
Selective reporting (reporting bias)	Unclear risk	
Other bias	Unclear risk	

Yildiz et al., 2004

**Table:** 

**Methods**
**Participants**
**Interventions**
**Outcomes**
**Notes**

Risk of bias table

**Table:** 

Bias	Authors’ judgement	Support for judgement
Random sequence generation (selection bias)	Unclear risk	
Allocation concealment (selection bias)	Unclear risk	
Blinding of participants and personnel (performance bias)	Unclear risk	
Blinding of outcome assessment (detection bias)	Unclear risk	
Incomplete outcome data (attrition bias)	Unclear risk	
Selective reporting (reporting bias)	Unclear risk	
Other bias	Unclear risk	

Zuurmond et al., 2018

**Table:** 

**Methods**
**Participants**
**Interventions**
**Outcomes**
**Notes**

Risk of bias table

**Table:** 

Bias	Authors’ judgement	Support for judgement
Random sequence generation (selection bias)	Unclear risk	
Allocation concealment (selection bias)	Unclear risk	
Blinding of participants and personnel (performance bias)	Unclear risk	
Blinding of outcome assessment (detection bias)	Unclear risk	
Incomplete outcome data (attrition bias)	Unclear risk	
Selective reporting (reporting bias)	Unclear risk	
Other bias	Unclear risk	

## SOURCES OF SUPPORT

**Internal sources**

- No sources of support provided

**External sources**

- This systematic review is supported by the UK Department of International Development (DFID) under its support for the Centre for Excellence for Development Impact and Learning (CEDIL) and the Programme for Evidence to iNform Disability Action (PENDA), UK.

This systematic review is supported by the UK Department of International Development (DFID) under its support for the Centre for Excellence for Development Impact and Learning (CEDIL) and the Programme for Evidence to iNform Disability Action (PENDA).

## Supporting information

Supporting information.

## Footnotes

^1^
It is important to note that the underlying assumption of some individual‐focused social inclusion interventions is not unproblematic: these programmes assume that you can equip individuals to improve their own social inclusion through for instance, improved mobility or social capital. This assumption may seem to elide the reality that large structural forces often drive social exclusion. However, they are an important avenue through which interventionists currently try to improve social inclusion outcomes, and so have been included here.
