# Supplementary material for: Effectiveness of interventions for improving social inclusion outcomes for people with disabilities in low‐ and middle‐income countries: A systematic review
Source: Campbell Syst Rev. 2023 Mar 21;19(1):e1316. doi: 10.1002/cl2.1316 (PMC10029810; doi:10.1002/cl2.1316)
Supplement: Supplementary file 1 — Supporting information. [file CL2-19-e1316-s001.docx]

Appendices

1 Search Strategy

**Disability EGM Update Searches – February 2020**

1. **Ovid MEDLINE(R) and In-Process & Other Non-Indexed Citations and Daily <1946 to February 25, 2020>Searched 26^th^ February 2020**

1 ((disable* or disabilit* or handicapped) adj5 (person* or people or child* or adolescen* or women or mother* or maternal or group*)).ti,kw. (16354)

2 ((physical* or intellectual* or learning or psychiatric* or sensory or motor or neuromotor or cognitive or mental* or developmental or communication or learning) adj2 (disabilit* or disabl* or handicap*)).ti,kw. (20515)

3 ((cognitive* or learning or mobility or sensory or visual* or vision or sight or hearing or physical* or mental* or intellectual*) adj2 (impair* or disabilit* or disabl* or handicap*)).ti,kw. (47712)

4 ((communication or language or speech or learning) adj5 disorder*).ti,kw. (4172)

5 ((depression or depressive or anxiety or psychiat* or well-being or quality of life or self-esteem or self perception) adj2 (impair* or disabilit* or disabl* or handicap*)).ti,kw. (2717)

6 mental health.ti,kw. (56608)

7 ((schizophreni* or psychos* or psychotic or schizoaffective or schizophreniform or dementia* or alzheimer*) adj2 (impair* or disabilit* or disabl* or handicap*)).ti,kw. (2932)

8 ((mental* or emotional* or psychiatric or neurologic*) adj2 (disorder* or ill or illness*)).ti,kw. (37898)

9 (autis* or dyslexi* or Down* syndrome or mongolism or trisomy 21).ti,kw. (54829)

10 ((intellectual* or educational* or mental* or psychological* or developmental) adj5 (impair* or retard* or deficien* or disable* or disabili* or handicap* or ill*)).ti,kw. (43259)

11 (((hearing or acoustic or ear*) adj5 (loss* or impair* or deficien* or disable* or disabili* or handicap*)) or deaf*).ti,kw. (43401)

12 (((visual* or vision or eye* or ocular) adj5 (loss* or impair* or deficien* or disable* or disabili* or handicap*)) or blind*).ti,kw. (73473)

13 ((cerebral pals* or spina bifida or muscular dystroph* or arthriti* or osteogenesis imperfecta or musculoskeletal abnormalit* or musculo-skeletal abnormalit* or muscular abnormalit* or skeletal abnormalit* or limb abnormalit* or brain injur* or amput* or clubfoot or polio* or paraplegi* or paralys* or paralyz* or hemiplegi* or stroke* or cerebrovascular accident*) adj2 (impair* or disabilit* or disabl* or handicap*)).ti,kw. (1278)

14 (physical* adj5 (impair* or deficien* or disable* or disabili* or handicap*)).ti,kw. (4015)

15 [exp disabled persons/ed, pc, rh, st, td, ut] (0)

16 exp *intellectual disability/ep, mo, pc or exp *developmental disabilities/ep, mo, pc or exp *child development disorders, pervasive/ep, mo, pc or exp *communication disorders/ep, mo, pc (9246)

17 exp *cerebral palsy/ep, mo, pc or exp *spina bifida cystica/ep, mo, pc or exp *spina bifida occulta/ep, mo, pc or exp *muscular dystrophies/ep, mo, pc or exp *arthritis/ep, mo, pc or exp *osteogenesis imperfecta/ep, mo, pc or exp *musculoskeletal abnormalities/ep, mo, pc or exp *brain injuries/ep, mo, pc or exp *amputation/ep, mo, pc or exp *clubfoot/ep, mo, pc or exp *poliomyelitis/ep, mo, pc or exp *paraplegia/ep, mo, pc or exp *hemiplegia/ep, mo, pc or exp *stroke/ep, mo, pc (43035)

18 exp *hearing loss/ep, mo, pc or exp *vision, low/ep, mo, pc or exp *deafness/ep, mo, pc or exp *blindness/ep, mo, pc (6464)

19 exp *“schizophrenia and disorders with psychotic features”/ep, mo, pc or exp *dementia/ep, mo, pc or exp *alzheimer disease/ep, mo, pc (17042)

20 exp *mental disorders/ep, mo, pc (119244)

21 exp *neurodevelopmental disorders/ep, mo, pc (12457)

22 or/1-20 (477974)

23 Developing Countries.sh,kf. (85006)

24 Africa/ or Asia/ or Caribbean/ or West Indies/ or Middle East/ or South America/ or Latin America/ or Central America/ (80823)

25 (Africa or Asia or Caribbean or West Indies or Middle East or South America or Latin America or Central America).tw. (190173)

26 (Afghanistan or Albania or Algeria or Angola or Argentina or Armenia or Armenian or Azerbaijan or Bangladesh or Benin or Byelarus or Byelorussian or Belarus or Belorussian or Belorussia or Belize or Bhutan or Bolivia or Bosnia or Herzegovina or Hercegovina or Botswana or Brazil or Bulgaria or Burkina Faso or Burkina Fasso or Upper Volta or Burundi or Urundi or Cambodia or Khmer Republic or Kampuchea or Cameroon or Cameroons or Cameron or Camerons or Cape Verde or Central African Republic or Chad or China or Colombia or Comoros or Comoro Islands or Comores or Mayotte or Congo or Zaire or Costa Rica or Cote d’Ivoire or Ivory Coast or Cuba or Djibouti or French Somaliland or Dominica or Dominican Republic or East Timor or East Timur or Timor Leste or Ecuador or Egypt or United Arab Republic or El Salvador or Eritrea or Ethiopia or Fiji or Gabon or Gabonese Republic or Gambia or Gaza or Georgia Republic or Georgian Republic or Ghana or Grenada or Guatemala or Guinea or Guiana or Guyana or Haiti or Honduras or India or Maldives or Indonesia or Iran or Iraq or Jamaica or Jordan or Kazakhstan or Kazakh or Kenya or Kiribati or Korea or Kosovo or Kyrgyzstan or Kirghizia or Kyrgyz Republic or Kirghiz or Kirgizstan or Lao PDR or Laos or Lebanon or Lesotho or Basutoland or Liberia or Libya or Macedonia or Madagascar or Malagasy Republic or Malaysia or Malaya or Malay or Sabah or Sarawak or Malawi or Mali or Marshall Islands or Mauritania or Mauritius or Agalega Islands or Mexico or Micronesia or Middle East or Moldova or Moldovia or Moldovian or Mongolia or Montenegro or Morocco or Ifni or Mozambique or Myanmar or Myanma or Burma or Namibia or Nepal or Netherlands Antilles or Nicaragua or Niger or Nigeria or Muscat or Pakistan or Palau or Palestine or Panama or Paraguay or Peru or Philippines or Philipines or Phillipines or Phillippines or Papua New Guinea or Romania or Rumania or Roumania or Rwanda or Ruanda or Saint Lucia or St Lucia or Saint Vincent or St Vincent or Grenadines or Samoa or Samoan Islands or Navigator Island or Navigator Islands or Sao Tome or Senegal or Serbia or Montenegro or Seychelles or Sierra Leone or Sri Lanka or Solomon Islands or Somalia or Sudan or Suriname or Surinam or Swaziland or South Africa or Syria or Tajikistan or Tadzhikistan or Tadjikistan or Tadzhik or Tanzania or Thailand or Togo or Togolese Republic or Tonga or Tunisia or Turkey or Turkmenistan or Turkmen or Uganda or Ukraine or Uzbekistan or Uzbek or Vanuatu or New Hebrides or Venezuela or Vietnam or Viet Nam or West Bank or Yemen or Zambia or Zimbabwe).tw. (1020832)

27 exp africa/ or algeria/ or egypt/ or libya/ or morocco/ or tunisia/ or cameroon/ or central african republic/ or chad/ or congo/ or “democratic republic of the congo”/ or equatorial guinea/ or gabon/ or burundi/ or djibouti/ or eritrea/ or ethiopia/ or kenya/ or rwanda/ or somalia/ or south sudan/ or sudan/ or tanzania/ or uganda/ or angola/ or botswana/ or lesotho/ or malawi/ or mozambique/ or namibia/ or south africa/ or swaziland/ or zambia/ or zimbabwe/ or benin/ or burkina faso/ or cape verde/ or cote d’ivoire/ or gambia/ or ghana/ or guinea/ or guinea-bissau/ or liberia/ or mali/ or mauritania/ or niger/ or nigeria/ or senegal/ or sierra leone/ or togo/ or americas/ or exp caribbean region/ or exp west indies/ or exp central america/ or belize/ or costa rica/ or el salvador/ or guatemala/ or honduras/ or nicaragua/ or panama/ or panama canal zone/ or latin america/ or mexico/ or exp south america/ or argentina/ or bolivia/ or brazil/ or chile/ or colombia/ or ecuador/ or french guiana/ or guyana/ or paraguay/ or peru/ or suriname/ or uruguay/ or venezuela/ or asia/ or asia, central/ or kazakhstan/ or kyrgyzstan/ or tajikistan/ or turkmenistan/ or uzbekistan/ or exp asia, southeastern/ or borneo/ or brunei/ or cambodia/ or timor-leste/ or indonesia/ or laos/ or malaysia/ or mekong valley/ or myanmar/ or philippines/ or singapore/ or thailand/ or vietnam/ or asia, western/ or bangladesh/ or bhutan/ or india/ or sikkim/ or middle east/ or afghanistan/ or bahrain/ or iran/ or iraq/ or israel/ or jordan/ or kuwait/ or lebanon/ or oman/ or qatar/ or saudi arabia/ or syria/ or turkey/ or united arab emirates/ or yemen/ or nepal/ or pakistan/ or sri lanka/ or far east/ or china/ or beijing/ or macau/ or tibet/ or korea/ or mongolia/ or taiwan/ or indian ocean islands/ or comoros/ or madagascar/ or mauritius/ or reunion/ or seychelles/ or pacific islands/ or exp melanesia/ or exp micronesia/ or polynesia/ or pitcairn island/ or exp samoa/ or tonga/ or prince edward island/ or west indies/ or “antigua and barbuda”/ or bahamas/ or barbados/ or cuba/ or dominica/ or dominican republic/ or grenada/ or guadeloupe/ or haiti/ or jamaica/ or martinique/ or netherlands antilles/ or puerto rico/ or “saint kitts and nevis”/ or saint lucia/ or “saint vincent and the grenadines”/ or “trinidad and tobago”/ or united states virgin islands/ or oceania/ (1095245)

28 ((developing or less* developed or under developed or underdeveloped or middle income or low* income or underserved or under served or deprived or poor*) adj (countr* or nation? or population? or world or state*)).ti,ab. (95470)

29 ((developing or less* developed or under developed or underdeveloped or middle income or low* income) adj (economy or economies)).ti,ab. (507)

30 (low* adj (gdp or gnp or gross domestic or gross national)).tw. (236)

31 (low adj3 middle adj3 countr*).tw. (14542)

32 (lmic or lmics or third world or lami countr*).tw. (6850)

33 transitional countr*.tw. (156)

34 or/23-33 (1649612)

35 ((systematic* or synthes*) adj3 (research or evaluation* or finding* or thematic* or report or descriptive or explanatory or narrative or meta* or review* or data or literature or studies or evidence or map or quantitative or study or studies or paper or impact or impacts or effect* or compar*)).ti,ab,kw. (328129)

36 (“meta regression” or “meta synth*“ or “meta-synth*“ or “meta analy*“ or “metaanaly*“ or “meta-analy*“ or “metanaly*“ or “metaregression” or “metaregression” or “methodologic* overview” or “pool* analys*“ or “pool* data” or “quantitative* overview” or “research integration”).ti,ab,kw. (174826)

37 (review adj3 (effectiveness or effects or systemat* or synth* or integrat* or map* or methodologic* or quantitative or evidence or literature)).ti,ab,kw. (404761)

38 (“meta ethnograph*“ or “meta synthesis” or (synthesis and (“qualitative literature” or “qualitative research”)) or “critical interpretive synthesis” or (“systematic review” and (“qualitative research” or “qualitative literature” or “qualitative stud*“)) or “thematic synthesis” or “framework synthesis” or “realist review” or “realist synthesis” or “qualitative systematic review*“ or “qualitative evidence synthes*“ or ((“quality assessment” or “critical appraisal” or “literature search*“) and (“qualitative research” or “qualitative literature” or “qualitative stud*“)) or (Noblit and Hare) or “meta narrative*“ or “narrative synthesis”).ti,ab,kw. (7484)

39 meta-analysis/ or evaluation studies/ or qualitative research/ or systematic review/ (481597)

40 controlled clinical trial/ or randomized controlled trial/ or equivalence trial/ or pragmatic clinical trial/ or case-control studies/ or retrospective studies/ or cohort studies/ or follow-up studies/ or longitudinal studies/ or prospective studies/ or epidemiologic methods/ or epidemiologic studies/ or controlled before-after studies/ or cross-sectional studies/ or interrupted time series analysis/ or control groups/ or cross-over studies/ or double-blind method/ or matched-pair analysis/ or meta-analysis as topic/ or random allocation/ or single-blind method/ or “retraction of publication”/ or case reports/ (4991800)

41 (random$ or placebo$ or single blind$ or double blind$ or triple blind$ or cohort$ or ((case$ or cohort or follow up or follow-up) adj2 (control$ or series or report$ or study or studies)) or retrospective$ or (observ$ adj3 (study or studies))).ti,ab,kw. (3137636)

42 or/35-41 (6813425)

43 22 and 34 and 42 (23469)

44 limit 43 to yr=“2018 -Current” (**4067)**

1. **Embase Classic+Embase (Ovid) <1947 to 2020 February 25>Searched 26^th^ February 2020**

1 ((disable* or disabilit* or handicapped) adj5 (person* or people or child* or adolescen* or women or mother* or maternal or group*)).ti,kw. (23241)

2 ((physical* or intellectual* or learning or psychiatric* or sensory or motor or neuromotor or cognitive or mental* or developmental or communication or learning) adj2 (disabilit* or disabl* or handicap*)).ti,kw. (31740)

3 ((cognitive* or learning or mobility or sensory or visual* or vision or sight or hearing or physical* or mental* or intellectual*) adj2 (impair* or disabilit* or disabl* or handicap*)).ti,kw. (81539)

4 ((communication or language or speech or learning) adj5 disorder*).ti,kw. (7284)

5 ((depression or depressive or anxiety or psychiat* or well-being or quality of life or self-esteem or self perception) adj2 (impair* or disabilit* or disabl* or handicap*)).ti,kw. (3071)

6 mental health.ti,kw. (75380)

7 ((schizophreni* or psychos* or psychotic or schizoaffective or schizophreniform or dementia* or alzheimer*) adj2 (impair* or disabilit* or disabl* or handicap*)).ti,kw. (4009)

8 ((mental* or emotional* or psychiatric or neurologic*) adj2 (disorder* or ill or illness*)).ti,kw. (58575)

9 (autis* or dyslexi* or Down* syndrome or mongolism or trisomy 21).ti,kw. (73218)

10 ((intellectual* or educational* or mental* or psychological* or developmental) adj5 (impair* or retard* or deficien* or disable* or disabili* or handicap* or ill*)).ti,kw. (63629)

11 (((hearing or acoustic or ear*) adj5 (loss* or impair* or deficien* or disable* or disabili* or handicap*)) or deaf*).ti,kw. (61540)

12 (((visual* or vision or eye* or ocular) adj5 (loss* or impair* or deficien* or disable* or disabili* or handicap*)) or blind*).ti,kw. (103804)

13 ((cerebral pals* or spina bifida or muscular dystroph* or arthriti* or osteogenesis imperfecta or musculoskeletal abnormalit* or musculo-skeletal abnormalit* or muscular abnormalit* or skeletal abnormalit* or limb abnormalit* or brain injur* or amput* or clubfoot or polio* or paraplegi* or paralys* or paralyz* or hemiplegi* or stroke* or cerebrovascular accident*) adj2 (impair* or disabilit* or disabl* or handicap*)).ti,kw. (1379)

14 (physical* adj5 (impair* or deficien* or disable* or disabili* or handicap*)).ti,kw. (5799)

15 exp *disability/dm, ep, pc, rh, th [Disease Management, Epidemiology, Prevention, Rehabilitation, Therapy] (3305)

16 exp *mental disease/dm, ep, pc, rh, th [Disease Management, Epidemiology, Prevention, Rehabilitation, Therapy] (241319)

17 exp *“disorders of higher cerebral function”/dm, ep, pc, rh, th [Disease Management, Epidemiology, Prevention, Rehabilitation, Therapy] (46137)

18 exp *developmental disorder/dm, ep, pc, rh, th [Disease Management, Epidemiology, Prevention, Rehabilitation, Therapy] (2226)

19 exp *disabled person/ (27928)

20 *cerebral palsy/dm, ep, pc, rh, th [Disease Management, Epidemiology, Prevention, Rehabilitation] (4546)

21 exp *spinal dysraphism/dm, ep, pc, rh, th [Disease Management, Epidemiology, Prevention, Rehabilitation, Therapy] (470)

22 exp *neural tube defect/dm, ep, pc, rh, th [Disease Management, Epidemiology, Prevention, Rehabilitation, Therapy] (2833)

23 exp *dystrophy/dm, ep, pc, rh, th [Disease Management, Epidemiology, Prevention, Rehabilitation, Therapy] (3481)

24 exp *arthritis/dm, ep, pc, rh, th [Disease Management, Epidemiology, Prevention, Rehabilitation, Therapy] (28190)

25 *osteogenesis imperfecta/dm, ep, pc, rh, th [Disease Management, Epidemiology, Prevention, Rehabilitation, Therapy] (299)

26 exp *musculoskeletal system malformation/dm, ep, pc, rh, th [Disease Management, Epidemiology, Prevention, Rehabilitation, Therapy] (5142)

27 exp *limb malformation/dm, ep, pc, rh, th [Disease Management, Epidemiology, Prevention, Rehabilitation, Therapy] (2389)

28 exp *brain malformation/dm, ep, pc, rh, th [Disease Management, Epidemiology, Prevention, Rehabilitation, Therapy] (2655)

29 exp *limb amputation/ (6728)

30 exp *clubfoot/dm, ep, pc, rh, th [Disease Management, Epidemiology, Prevention, Rehabilitation, Therapy] (697)

31 exp *poliomyelitis/dm, ep, pc, rh, th [Disease Management, Epidemiology, Prevention, Rehabilitation, Therapy] (4509)

32 exp *paralysis/dm, ep, pc, rh, th [Disease Management, Epidemiology, Prevention, Rehabilitation, Therapy] (18455)

33 exp *cerebrovascular accident/dm, ep, pc, rh, th [Disease Management, Epidemiology, Prevention, Rehabilitation, Therapy] (14445)

34 exp *hearing disorder/dm, ep, pc, rh, th [Disease Management, Epidemiology, Prevention, Rehabilitation, Therapy] (12848)

35 exp *hearing impairment/dm, ep, pc, rh, th [Disease Management, Epidemiology, Prevention, Rehabilitation, Therapy] (9118)

36 exp *visual disorder/dm, ep, pc, rh, th [Disease Management, Epidemiology, Prevention, Rehabilitation, Therapy] (13295)

37 or/1-36 (800216)

38 developing country/ (95013)

39 low income country/ (5340)

40 middle income country/ (7663)

41 ((developing or less* developed or under developed or underdeveloped or middle income or low* income or underserved or under served or deprived or poor*) adj (economy or economies)).ti,ab. (801)

42 ((developing or less* developed or under developed or underdeveloped or middle income or low* income or underserved or under served or deprived or poor*) adj (countr* or nation? or population? or world)).ti,ab. (124055)

43 (low* adj (gdp or gnp or gross domestic or gross national)).ti,ab. (344)

44 (low adj3 middle adj3 countr*).ti,ab. (17826)

45 (lmic or lmics or third world or lami countr*).ti,ab. (8820)

46 transitional countr*.ti,ab. (226)

47 global south.ti,ab. (351)

48 “Africa south of the Sahara”/ (13771)

49 (“africa south of the sahara” or sub-saharan africa or central africa or eastern africa or southern africa or western africa).ti,ab. (32858)

50 Botswana/ (2676)

51 (Botswana or Bechuanaland or Kalahari).ti,ab. (2905)

52 Equatorial Guinea/ (467)

53 (Equatorial Guinea or Spanish Guinea).ti,ab. (563)

54 Gabon/ (1749)

55 (Gabon or Gabonese Republic).ti,ab. (1919)

56 Mauritius/ (892)

57 (Mauritius or Agalega Islands).ti,ab. (1035)

58 Namibia/ (1579)

59 Namibia.ti,ab. (1611)

60 South Africa/ (51310)

61 South Africa.ti,ab. (39328)

62 Angola/ (1461)

63 angola.ti,ab. (1570)

64 Cameroon/ (7093)

65 Cameroon.ti,ab. (7783)

66 Cape Verde/ (353)

67 (Cape Verde or Cabo Verde).ti,ab. (598)

68 Congo/ (4008)

69 (congo not ((democratic republic adj3 congo) or congo red or crimean-congo)).ti,ab. (3388)

70 Cote d’Ivoire/ (3321)

71 (Cote d’Ivoire or Ivory Coast).ti,ab. (4378)

72 Ghana/ (11563)

73 (Ghana or Gold Coast).ti,ab. (12283)

74 Kenya/ (21482)

75 kenya.mp. (25409)

76 Lesotho/ (697)

77 (Lesotho or Basutoland).ti,ab. (767)

78 Mauritania/ (638)

79 Mauritania.ti,ab. (670)

80 Nigeria/ (38425)

81 Nigeria.ti,ab. (35599)

82 “Sao Tome and Principe”/ (77)

83 (sao tome adj2 principe).ti,ab. (155)

84 Sudan/ (7300)

85 (Sudan not south sudan).ti,ab. (9827)

86 Swaziland/ (915)

87 Swaziland.ti,ab. (943)

88 Zambia/ (6263)

89 (Zambia or Northern Rhodesia).ti,ab. (5971)

90 Benin/ (2499)

91 (Benin or Dahomey).ti,ab. (4713)

92 Burkina Faso/ (4263)

93 (Burkina Faso or Burkina Fasso or Upper Volta).ti,ab. (4940)

94 Burundi/ (852)

95 Burundi.ti,ab. (887)

96 Central African Republic/ (927)

97 (Central African Republic or Ubangi-Shari).ti,ab. (1108)

98 Chad/ (994)

99 Chad.ti,ab. (1402)

100 Comoros/ (332)

101 (Comoros or Comoro Islands or Mayotte or Iles Comores).ti,ab. (608)

102 “Democratic Republic Congo”/ (4183)

103 ((democratic republic adj2 congo) or belgian congo or zaire).ti,ab. (4885)

104 Eritrea/ (557)

105 Eritrea.ti,ab. (651)

106 Ethiopia/ (16392)

107 Ethiopia.ti,ab. (15662)

108 Gambia/ (2816)

109 Gambia.ti,ab. (2543)

110 Guinea/ (2716)

111 (Guinea not (New Guinea or Guinea Pig* or Guinea Fowl)).ti,ab. (4748)

112 Guinea-Bissau/ (1086)

113 (Guinea-Bissau or Portuguese Guinea).ti,ab. (1124)

114 Liberia/ (1781)

115 Liberia.ti,ab. (1793)

116 Madagascar/ (4596)

117 (Madagascar or Malagasy Republic).ti,ab. (5272)

118 Malawi/ (7318)

119 (Malawi or Nyasaland).ti,ab. (7711)

120 Mali/ (3518)

121 Mali.ti,ab. (4351)

122 Mozambique/ (3717)

123 (Mozambique or Mocambique or Portuguese East Africa).ti,ab. (4027)

124 Niger/ (2444)

125 (Niger not (Aspergillus or Peptococcus or Schizothorax or Cruciferae or Gobius or Lasius or Agelastes or Melanosuchus or radish or Parastromateus or Orius or Apergillus or Parastromateus or Stomoxys)).ti,ab. (4238)

126 Rwanda/ (3571)

127 (Rwanda or Ruanda).ti,ab. (3537)

128 Senegal/ (6989)

129 senegal.ti,ab. (6820)

130 Sierra Leone/ (2344)

131 Sierra Leone.mp. (3003)

132 exp Somalia/ (2010)

133 Somalia.ti,ab. (1454)

134 South Sudan/ (274)

135 south sudan.ti,ab. (597)

136 Tanzania/ (15236)

137 (Tanzania or Tanganyika or Zanzibar).ti,ab. (15351)

138 Togo/ (1458)

139 (Togo or Togolese Republic).ti,ab. (1669)

140 Uganda/ (18170)

141 Uganda.ti,ab. (17168)

142 Zimbabwe/ (6973)

143 (Zimbabwe or Rhodesia).ti,ab. (6427)

144 Maldives/ (277)

145 Maldives.ti,ab. (324)

146 Algeria/ (4938)

147 Algeria.ti,ab. (4911)

148 Iran/ (49002)

149 Iran.ti,ab. (50966)

150 exp Iraq/ (8629)

151 Iraq.ti,ab. (9387)

152 Jordan/ (6583)

153 Jordan.ti,ab. (7808)

154 Lebanon/ (5802)

155 Lebanon.ti,ab. (5437)

156 Libyan Arab Jamahiriya/ (1705)

157 Libya.ti,ab. (1501)

158 Argentina/ (22507)

159 Argentina.ti,ab. (21909)

160 Belize/ (775)

161 Belize.ti,ab. (858)

162 exp Brazil/ (113311)

163 Brazil.ti,ab. (99808)

164 Colombia/ (19388)

165 Colombia.ti,ab. (16497)

166 Costa Rica/ (4882)

167 Costa Rica.ti,ab. (5140)

168 Cuba/ (6958)

169 Cuba.ti,ab. (5931)

170 Dominica/ (185)

171 Dominica.ti,ab. (522)

172 Dominican Republic/ (2504)

173 Dominican Republic.ti,ab. (2297)

174 Ecuador/ (5348)

175 Ecuador.ti,ab. (5330)

176 Grenada/ (261)

177 Grenada.ti,ab. (372)

178 Guyana/ (1026)

179 Guyana.mp. (1578)

180 Jamaica/ (4350)

181 Jamaica.ti,ab. (3990)

182 Mexico/ (46440)

183 Mexico.ti,ab. (51211)

184 exp Panama/ (3314)

185 Panama.ti,ab. (4295)

186 Paraguay/ (1486)

187 Paraguay.mp. (2190)

188 Peru/ (12235)

189 Peru.ti,ab. (12771)

190 Saint Lucia/ (123)

191 (St Lucia or Saint Lucia).ti,ab. (385)

192 “Saint Vincent and the Grenadines”/ (72)

193 Grenadines.ti,ab. (88)

194 Suriname/ (1259)

195 Suriname.ti,ab. (686)

196 Venezuela/ (6804)

197 Venezuela.ti,ab. (6557)

198 Albania/ (1816)

199 Albania.ti,ab. (1661)

200 Azerbaijan/ (1832)

201 Azerbaijan.ti,ab. (1941)

202 Belarus/ (2831)

203 (belarus or byelarus or belorussia).ti,ab. (2243)

204 exp “Bosnia and Herzegovina”/ (2723)

205 (bosnia or herzegovina).ti,ab. (2923)

206 Bulgaria/ (9799)

207 Bulgaria.ti,ab. (6723)

208 Croatia/ (9946)

209 croatia.ti,ab. (9492)

210 Kazakhstan/ (3943)

211 (Kazakhstan or kazakh).ti,ab. (4003)

212 “Macedonia (Republic)”/ (1107)

213 Macedonia.ti,ab. (1939)

214 “Montenegro (republic)”/ (718)

215 Montenegro.ti,ab. (1135)

216 Romania/ (14558)

217 Romania.ti,ab. (9271)

218 exp Russian Federation/ (63691)

219 USSR/ (48320)

220 (Russia or Russian Federation or USSR or Union of Soviet Socialist Republics or Soviet Union).mp. (121177)

221 exp Serbia/ (5714)

222 serbia.ti,ab. (6678)

223 “Turkey (republic)”/ (36041)

224 turkey.ti,ab. not animal/ (42306)

225 Turkmenistan/ (630)

226 Turkmenistan.ti,ab. (369)

227 Yugoslavia/ (9598)

228 yugoslavia.ti,ab. (2903)

229 exp Samoan Islands/ (981)

230 american samoa.ti,ab. (412)

231 exp China/ (221199)

232 china.ti,ab. (200641)

233 Fiji/ (1509)

234 fiji.ti,ab. (2109)

235 Malaysia/ (21974)

236 malaysia.ti,ab. (20542)

237 Marshall Islands/ (165)

238 marshall islands.ti,ab. (348)

239 Nauru/ (67)

240 nauru.ti,ab. (164)

241 (“independent state of samoa” or (samoa not american samoa) or western samoa or navigator islands or samoan islands).ti,ab. (634)

242 Thailand/ (33871)

243 Thailand.ti,ab. (31471)

244 Tonga/ (360)

245 tonga.ti,ab. (470)

246 Tuvalu/ (45)

247 Tuvalu.ti,ab. (64)

248 Bangladesh/ (15971)

249 Bangladesh.ti,ab. (15950)

250 Bhutan/ (772)

251 Bhutan.ti,ab. (759)

252 exp India/ (150311)

253 India.ti,ab. (132004)

254 exp Pakistan/ (27273)

255 Pakistan.ti,ab. (23991)

256 Sri Lanka/ (8633)

257 Sri Lanka.ti,ab. (7623)

258 Djibouti/ (348)

259 (Djibouti or French Somaliland).ti,ab. (438)

260 Egypt/ (21006)

261 Egypt.ti,ab. (18683)

262 Jordan/ (6583)

263 Jordan.ti,ab. (7808)

264 Morocco/ (7791)

265 Morocco.ti,ab. (7288)

266 Syrian Arab Republic/ (2609)

267 (Syria or Syrian Arab Republic).ti,ab. (2362)

268 Tunisia/ (9911)

269 tunisia.mp. (12045)

270 Palestine/ (1670)

271 Gaza.ti,ab. (1223)

272 Yemen/ (2034)

273 Yemen.ti,ab. (2093)

274 Bolivia/ (3567)

275 Bolivia.ti,ab. (3704)

276 El Salvador/ (1944)

277 El Salvador.ti,ab. (1528)

278 Guatemala/ (4528)

279 Guatemala.ti,ab. (4254)

280 Honduras/ (1973)

281 Honduras.ti,ab. (2064)

282 Nicaragua/ (2255)

283 Nicaragua.ti,ab. (2241)

284 Armenia/ (1985)

285 Armenia.ti,ab. (1533)

286 “Georgia (Republic)”/ (1969)

287 Kosovo/ (496)

288 Kosovo.ti,ab. (1225)

289 Kyrgyzstan/ (1656)

290 (kyrgyzstan or kyrgyz republic or kirghizia or kirghiz).ti,ab. (1257)

291 Moldova/ (1169)

292 Moldova.ti,ab. (886)

293 Tajikistan/ (965)

294 tajikistan.ti,ab. (690)

295 exp Ukraine/ (16664)

296 Ukraine.ti,ab. (6681)

297 Uzbekistan/ (2223)

298 Uzbekistan.ti,ab. (1483)

299 Cambodia/ (4970)

300 cambodia.ti,ab. (4637)

301 exp Indonesia/ (17860)

302 indonesia.ti,ab. (17231)

303 Kiribati/ (112)

304 Kiribati.ti,ab. (181)

305 Laos/ (2166)

306 (laos or (lao adj1 democratic republic)).ti,ab. (2081)

307 “Marshall Islands”/ (165)

308 “Federated States of Micronesia”/ (956)

309 (marshall island* or caroline island* or ellice island* or gilbert island* or johnston island* or mariana island* or micronesia or pacific island*).ti,ab. (8316)

310 Mongolia/ (3163)

311 mongolia.ti,ab. (4696)

312 Myanmar/ (3882)

313 (myanmar or burma).ti,ab. (4557)

314 Papua New Guinea/ (6672)

315 Papua New Guinea.ti,ab. (4829)

316 Philippines/ (11887)

317 Philippines.ti,ab. (10107)

318 Timor-Leste/ (569)

319 Timor-Leste.ti,ab. (368)

320 Vanuatu/ (448)

321 Vanuatu.ti,ab. (655)

322 Viet Nam/ (16683)

323 (Viet Nam or vietnam).ti,ab. (17580)

324 Afghanistan/ (5860)

325 Afghanistan.ti,ab. (6831)

326 Nepal/ (11509)

327 Nepal.ti,ab. (11194)

328 Haiti/ (4193)

329 Haiti.ti,ab. (3779)

330 “North Korea”/ (558)

331 (north korea or (democratic people* republic adj2 korea)).ti,ab. (455)

332 or/38-331 [ALL LMICs] (1746480)

333 ((systematic* or synthes*) adj3 (research or evaluation* or finding* or thematic* or report or descriptive or explanatory or narrative or meta* or review* or data or literature or studies or evidence or map or quantitative or study or studies or paper or impact or impacts or effect* or compar*)).ti,ab,kw. (421979)

334 (“meta regression” or “meta synth*“ or “meta-synth*“ or “meta analy*“ or “metaanaly*“ or “meta-analy*“ or “metanaly*“ or “metaregression” or “metaregression” or “methodologic* overview” or “pool* analys*“ or “pool* data” or “quantitative* overview” or “research integration”).ti,ab,kw. (244165)

335 (review adj3 (effectiveness or effects or systemat* or synth* or integrat* or map* or methodologic* or quantitative or evidence or literature)).ti,ab,kw. (523786)

336 (“meta ethnograph*“ or “meta synthesis” or (synthesis and (“qualitative literature” or “qualitative research”)) or “critical interpretive synthesis” or (“systematic review” and (“qualitative research” or “qualitative literature” or “qualitative stud*“)) or “thematic synthesis” or “framework synthesis” or “realist review” or “realist synthesis” or “qualitative systematic review*“ or “qualitative evidence synthes*“ or ((“quality assessment” or “critical appraisal” or “literature search*“) and (“qualitative research” or “qualitative literature” or “qualitative stud*“)) or (Noblit and Hare) or “meta narrative*“ or “narrative synthesis”).ti,ab,kw. (9028)

337 “systematic review”/ or meta analysis/ or exp evaluation study/ or qualitative analysis/ or qualitative research/ (515422)

338 (random$ or placebo$ or single blind$ or double blind$ or triple blind$ or cohort$ or ((case$ or cohort or follow up or follow-up) adj2 (control$ or series or report$ or study or studies)) or retrospective$ or (observ$ adj3 (study or studies))).ti,ab,kw. (4777384)

339 controlled clinical trial/ or randomized controlled trial/ or case control study/ or population based case control study/ or retrospective study/ or cohort analysis/ or follow up/ or longitudinal study/ or prospective study/ or cross-sectional study/ or times series analysis/ or control group/ or randomization/ (4102624)

340 or/333-339 (7259102)

341 37 and 332 and 340 (21356)

342 limit 341 to yr=“2018 -Current” (4482)

343 limit 342 to embase (**3275**)

1. **PsycINFO (Ovid) <1806 to February Week 3 2020>Searched 26^th^ February 2020**

1 ((disable* or disabilit* or handicapped) adj5 (person* or people or child* or adolescen* or women or mother* or maternal or group*)).ti,hw. (22522)

2 ((physical* or intellectual* or learning or psychiatric* or sensory or motor or neuromotor or cognitive or mental* or developmental or communication or learning) adj2 (disabilit* or disabl* or handicap*)).ti,hw. (48731)

3 ((cognitive* or learning or mobility or sensory or visual* or vision or sight or hearing or physical* or mental* or intellectual*) adj2 (impair* or disabilit* or disabl* or handicap*)).ti,hw. (81483)

4 ((communication or language or speech or learning) adj5 disorder*).ti,hw. (19968)

5 ((depression or depressive or anxiety or psychiat* or well-being or quality of life or self-esteem or self perception) adj2 (impair* or disabilit* or disabl* or handicap*)).ti,hw. (1756)

6 mental health.ti,hw. (126256)

7 ((schizophreni* or psychos* or psychotic or schizoaffective or schizophreniform or dementia* or alzheimer*) adj2 (impair* or disabilit* or disabl* or handicap*)).ti,hw. (2036)

8 ((mental* or emotional* or psychiatric or neurologic*) adj2 (disorder* or ill or illness*)).ti,hw. (97032)

9 (autis* or dyslexi* or Down* syndrome or mongolism or trisomy 21).ti,hw. (56753)

10 ((intellectual* or educational* or mental* or psychological* or developmental) adj5 (impair* or retard* or deficien* or disable* or disabili* or handicap* or ill*)).ti,hw. (62656)

11 (((hearing or acoustic or ear*) adj5 (loss* or impair* or deficien* or disable* or disabili* or handicap*)) or deaf*).ti,hw. (20671)

12 (((visual* or vision or eye* or ocular) adj5 (loss* or impair* or deficien* or disable* or disabili* or handicap*)) or blind*).ti,hw. (20068)

13 ((cerebral pals* or spina bifida or muscular dystroph* or arthriti* or osteogenesis imperfecta or musculoskeletal abnormalit* or musculo-skeletal abnormalit* or muscular abnormalit* or skeletal abnormalit* or limb abnormalit* or brain injur* or amput* or clubfoot or polio* or paraplegi* or paralys* or paralyz* or hemiplegi* or stroke* or cerebrovascular accident*) adj2 (impair* or disabilit* or disabl* or handicap*)).ti,hw. (276)

14 (physical* adj5 (impair* or deficien* or disable* or disabili* or handicap*)).ti,hw. (4552)

15 exp *disabilities/ (45529)

16 exp *mental disorders/ (753876)

17 exp *communication disorders/ (53729)

18 exp *physical disorders/ (510075)

19 exp *intellectual development disorder/ or exp *brain damage/ or *cognitive impairment/ (72524)

20 poliomyelitis/ (259)

21 exp amputation/ (1470)

22 exp *paralysis/ or exp *central nervous system disorders/ or *dysarthria/ or exp *spinal cord injuries/ (197900)

23 exp *movement disorders/ (27400)

24 or/1-23 (1315904)

25 developing countries/ or emerging economies/ (6282)

26 (Africa or Asia or Caribbean or West Indies or Middle East or South America or Latin America or Central America).lo,tw. (49922)

27 (Afghanistan or Albania or Algeria or Angola or Argentina or Armenia or Armenian or Azerbaijan or Bangladesh or Benin or Byelarus or Byelorussian or Belarus or Belorussian or Belorussia or Belize or Bhutan or Bolivia or Bosnia or Herzegovina or Hercegovina or Botswana or Brazil or Bulgaria or Burkina Faso or Burkina Fasso or Upper Volta or Burundi or Urundi or Cambodia or Khmer Republic or Kampuchea or Cameroon or Cameroons or Cameron or Camerons or Cape Verde or Central African Republic or Chad or China or Colombia or Comoros or Comoro Islands or Comores or Mayotte or Congo or Zaire or Costa Rica or Cote d’Ivoire or Ivory Coast or Cuba or Djibouti or French Somaliland or Dominica or Dominican Republic or East Timor or East Timur or Timor Leste or Ecuador or Egypt or United Arab Republic or El Salvador or Eritrea or Ethiopia or Fiji or Gabon or Gabonese Republic or Gambia or Gaza or Georgia Republic or Georgian Republic or Ghana or Grenada or Guatemala or Guinea or Guiana or Guyana or Haiti or Honduras or India or Maldives or Indonesia or Iran or Iraq or Jamaica or Jordan or Kazakhstan or Kazakh or Kenya or Kiribati or Korea or Kosovo or Kyrgyzstan or Kirghizia or Kyrgyz Republic or Kirghiz or Kirgizstan or Lao PDR or Laos or Lebanon or Lesotho or Basutoland or Liberia or Libya or Macedonia or Madagascar or Malagasy Republic or Malaysia or Malaya or Malay or Sabah or Sarawak or Malawi or Mali or Marshall Islands or Mauritania or Mauritius or Agalega Islands or Mexico or Micronesia or Middle East or Moldova or Moldovia or Moldovian or Mongolia or Montenegro or Morocco or Ifni or Mozambique or Myanmar or Myanma or Burma or Namibia or Nepal or Netherlands Antilles or Nicaragua or Niger or Nigeria or Muscat or Pakistan or Palau or Palestine or Panama or Paraguay or Peru or Philippines or Philipines or Phillipines or Phillippines or Papua New Guinea or Romania or Rumania or Roumania or Rwanda or Ruanda or Saint Lucia or St Lucia or Saint Vincent or St Vincent or Grenadines or Samoa or Samoan Islands or Navigator Island or Navigator Islands or Sao Tome or Senegal or Serbia or Montenegro or Seychelles or Sierra Leone or Sri Lanka or Solomon Islands or Somalia or Sudan or Suriname or Surinam or Swaziland or South Africa or Syria or Tajikistan or Tadzhikistan or Tadjikistan or Tadzhik or Tanzania or Thailand or Togo or Togolese Republic or Tonga or Tunisia or Turkey or Turkmenistan or Turkmen or Uganda or Ukraine or Uzbekistan or Uzbek or Vanuatu or New Hebrides or Venezuela or Vietnam or Viet Nam or West Bank or Yemen or Zambia or Zimbabwe).lo,tw. (272978)

28 ((developing or less* developed or under developed or underdeveloped or middle income or low* income or underserved or under served or deprived or poor*) adj (countr* or nation? or population? or world or state*)).ti,ab. (17560)

29 ((developing or less* developed or under developed or underdeveloped or middle income or low* income) adj (economy or economies)).ti,ab. (366)

30 (low* adj (gdp or gnp or gross domestic or gross national)).tw. (45)

31 (lmic or lmics or third world or lami countr*).tw. (1862)

32 transitional countr*.tw. (66)

33 or/25-32 (297854)

34 ((systematic* or synthes*) adj3 (research or evaluation* or finding* or thematic* or report or descriptive or explanatory or narrative or meta* or review* or data or literature or studies or evidence or map or quantitative or study or studies or paper or impact or impacts or effect* or compar*)).ti,ab. (62108)

35 (“meta regression” or “meta synth*“ or “meta-synth*“ or “meta analy*“ or “metaanaly*“ or “meta-analy*“ or “metanaly*“ or “metaregression” or “metaregression” or “methodologic* overview” or “pool* analys*“ or “pool* data” or “quantitative* overview” or “research integration”).ti,ab. (38042)

36 (review adj3 (effectiveness or effects or systemat* or synth* or integrat* or map* or methodologic* or quantitative or evidence or literature)).ti,ab. (87936)

37 (“meta ethnograph*“ or “meta synthesis” or (synthesis and (“qualitative literature” or “qualitative research”)) or “critical interpretive synthesis” or (“systematic review” and (“qualitative research” or “qualitative literature” or “qualitative stud*“)) or “thematic synthesis” or “framework synthesis” or “realist review” or “realist synthesis” or “qualitative systematic review*“ or “qualitative evidence synthes*“ or ((“quality assessment” or “critical appraisal” or “literature search*“) and (“qualitative research” or “qualitative literature” or “qualitative stud*“)) or (Noblit and Hare) or “meta narrative*“ or “narrative synthesis”).ti,ab. (3267)

38 experimental design/ or clinical trials/ or cohort analysis/ or followup studies/ or exp longitudinal studies/ or qualitative research/ or quantitative methods/ or quasi experimental methods/ or cohort analysis/ or retrospective studies/ or time series/ or case report/ or meta analysis/ or systematic review/ (91294)

39 or/34-38 (226160)

40 24 and 33 and 39 (4225)

41 limit 40 to yr=“2018 -Current” (**624**)

1. **CAB Global Health (Ovid) <1910 to 2020 Week 07>Searched 26^th^ Feb 2020**

1 ((disable* or disabilit* or handicapped) adj5 (person* or people or child* or adolescen* or women or mother* or maternal or group*)).ti. (1594)

2 ((physical* or intellectual* or learning or psychiatric* or sensory or motor or neuromotor or cognitive or mental* or developmental or communication or learning) adj2 (disabilit* or disabl* or handicap*)).ti. (1204)

3 ((cognitive* or learning or mobility or sensory or visual* or vision or sight or hearing or physical* or mental* or intellectual*) adj2 (impair* or disabilit* or disabl* or handicap*)).ti. (3692)

4 ((communication or language or speech or learning) adj5 disorder*).ti. (104)

5 ((depression or depressive or anxiety or psychiat* or well-being or quality of life or self-esteem or self perception) adj2 (impair* or disabilit* or disabl* or handicap*)).ti. (126)

6 mental health.ti. (6986)

7 ((schizophreni* or psychos* or psychotic or schizoaffective or schizophreniform or dementia* or alzheimer*) adj2 (impair* or disabilit* or disabl* or handicap*)).ti. (167)

8 ((mental* or emotional* or psychiatric or neurologic*) adj2 (disorder* or ill or illness*)).ti. (3181)

9 (autis* or dyslexi* or Down* syndrome or mongolism or trisomy 21).ti. (2888)

10 ((intellectual* or educational* or mental* or psychological* or developmental) adj5 (impair* or retard* or deficien* or disable* or disabili* or handicap* or ill*)).ti. (2519)

11 (((hearing or acoustic or ear*) adj5 (loss* or impair* or deficien* or disable* or disabili* or handicap*)) or deaf*).ti. (2517)

12 (((visual* or vision or eye* or ocular) adj5 (loss* or impair* or deficien* or disable* or disabili* or handicap*)) or blind*).ti. (9722)

13 ((cerebral pals* or spina bifida or muscular dystroph* or arthriti* or osteogenesis imperfecta or musculoskeletal abnormalit* or musculo-skeletal abnormalit* or muscular abnormalit* or skeletal abnormalit* or limb abnormalit* or brain injur* or amput* or clubfoot or polio* or paraplegi* or paralys* or paralyz* or hemiplegi* or stroke* or cerebrovascular accident*) adj2 (impair* or disabilit* or disabl* or handicap*)).ti. (32)

14 (physical* adj5 (impair* or deficien* or disable* or disabili* or handicap*)).ti. (561)

15 people with disabilities/ or children with disabilities/ or people with mental disabilities/ or people with physical disabilities/ (4785)

16 abnormalities/ or exp congenital abnormalities/ or exp deformities/ or exp disabilities/ or exp malformations/ (32518)

17 exp mental disorders/ or exp mental health/ or learning disabilities/ or paralysis/ or paraparesis/ or paraplegia/ or poliomyelitis/ or hearing impairment/ or deafness/ or people with hearing impairment/ or vision disorders/ or blindness/ or people with visual impairment/ (103874)

18 or/1-17 (146038)

19 exp africa/ (259467)

20 exp Central America/ or exp Latin America/ or exp South America/ (192441)

21 mexico/ (23681)

22 exp central asia/ (13399)

23 east asia/ or china/ or korea democratic people’s republic/ or korea republic/ or mongolia/ (257544)

24 exp south asia/ or himalaya/ (161877)

25 exp south east asia/ or pacific rim/ (104598)

26 exp caribbean/ (22864)

27 exp pacific islands/ (14813)

28 exp developing countries/ (970661)

29 (Africa or Asia or Caribbean or West Indies or South America or Latin America or Central America).tw. (1090170)

30 (Afghanistan or Albania or Algeria or Angola or Argentina or Armenia or Armenian or Azerbaijan or Bangladesh or Benin or Byelarus or Byelorussian or Belarus or Belorussian or Belorussia or Belize or Bhutan or Bolivia or Bosnia or Herzegovina or Hercegovina or Botswana or Brazil or Bulgaria or Burkina Faso or Burkina Fasso or Upper Volta or Burundi or Urundi or Cambodia or Khmer Republic or Kampuchea or Cameroon or Cameroons or Cameron or Camerons or Cape Verde or Central African Republic or Chad or China or Colombia or Comoros or Comoro Islands or Comores or Mayotte or Congo or Zaire or Costa Rica or Cote d’Ivoire or Ivory Coast or Cuba or Djibouti or French Somaliland or Dominica or Dominican Republic or East Timor or East Timur or Timor Leste or Ecuador or Egypt or United Arab Republic or El Salvador or Eritrea or Ethiopia or Fiji or Gabon or Gabonese Republic or Gambia or Gaza or Georgia Republic or Georgian Republic or Ghana or Grenada or Guatemala or Guinea or Guiana or Guyana or Haiti or Honduras or India or Maldives or Indonesia or Iran or Iraq or Jamaica or Jordan or Kazakhstan or Kazakh or Kenya or Kiribati or Korea or Kosovo or Kyrgyzstan or Kirghizia or Kyrgyz Republic or Kirghiz or Kirgizstan or Lao PDR or Laos or Lebanon or Lesotho or Basutoland or Liberia or Libya or Macedonia or Madagascar or Malagasy Republic or Malaysia or Malaya or Malay or Sabah or Sarawak or Malawi or Mali or Marshall Islands or Mauritania or Mauritius or Agalega Islands or Mexico or Micronesia or Middle East or Moldova or Moldovia or Moldovian or Mongolia or Montenegro or Morocco or Ifni or Mozambique or Myanmar or Myanma or Burma or Namibia or Nepal or Netherlands Antilles or Nicaragua or Niger or Nigeria or Muscat or Pakistan or Palau or Palestine or Panama or Paraguay or Peru or Philippines or Philipines or Phillipines or Phillippines or Papua New Guinea or Romania or Rumania or Roumania or Rwanda or Ruanda or Saint Lucia or St Lucia or Saint Vincent or St Vincent or Grenadines or Samoa or Samoan Islands or Navigator Island or Navigator Islands or Sao Tome or Senegal or Serbia or Montenegro or Seychelles or Sierra Leone or Sri Lanka or Solomon Islands or Somalia or Sudan or Suriname or Surinam or Swaziland or South Africa or Syria or Tajikistan or Tadzhikistan or Tadjikistan or Tadzhik or Tanzania or Thailand or Togo or Togolese Republic or Tonga or Tunisia or Turkey or Turkmenistan or Turkmen or Uganda or Ukraine or Uzbekistan or Uzbek or Vanuatu or New Hebrides or Venezuela or Vietnam or Viet Nam or West Bank or Yemen or Zambia or Zimbabwe).tw. (1062515)

31 ((developing or less* developed or under developed or underdeveloped or middle income or low* income or underserved or under served or deprived or poor*) adj (countr* or nation? or population? or world or state*)).ti,ab. (55654)

32 ((developing or less* developed or under developed or underdeveloped or middle income or low* income) adj (economy or economies)).ti,ab. (301)

33 (low* adj (gdp or gnp or gross domestic or gross national)).tw. (73)

34 (low adj3 middle adj3 countr*).tw. (8189)

35 (lmic or lmics or third world or lami countr*).tw. (24530)

36 transitional countr*.tw. (98)

37 or/19-35 (1221707)

38 ((systematic* or synthes*) adj3 (research or evaluation* or finding* or thematic* or report or descriptive or explanatory or narrative or meta* or review* or data or literature or studies or evidence or map or quantitative or study or studies or paper or impact or impacts or effect* or compar*)).ti,ab. (52329)

39 (“meta regression” or “meta synth*“ or “meta-synth*“ or “meta analy*“ or “metaanaly*“ or “meta-analy*“ or “metanaly*“ or “metaregression” or “metaregression” or “methodologic* overview” or “pool* analys*“ or “pool* data” or “quantitative* overview” or “research integration”).ti,ab. (35021)

40 (review adj3 (effectiveness or effects or systemat* or synth* or integrat* or map* or methodologic* or quantitative or evidence or literature)).ti,ab. (60987)

41 (“meta ethnograph*“ or “meta synthesis” or (synthesis and (“qualitative literature” or “qualitative research”)) or “critical interpretive synthesis” or (“systematic review” and (“qualitative research” or “qualitative literature” or “qualitative stud*“)) or “thematic synthesis” or “framework synthesis” or “realist review” or “realist synthesis” or “qualitative systematic review*“ or “qualitative evidence synthes*“ or ((“quality assessment” or “critical appraisal” or “literature search*“) and (“qualitative research” or “qualitative literature” or “qualitative stud*“)) or (Noblit and Hare) or “meta narrative*“ or “narrative synthesis”).ti,ab. (1545)

42 meta-analysis/ or evaluation studies/ or qualitative research/ (26345)

43 controlled clinical trial/ or randomized controlled trial/ or equivalence trial/ or pragmatic clinical trial/ or case-control studies/ or retrospective studies/ or cohort studies/ or follow-up studies/ or longitudinal studies/ or prospective studies/ or epidemiologic methods/ or epidemiologic studies/ or controlled before-after studies/ or cross-sectional studies/ or interrupted time series analysis/ or control groups/ or cross-over studies/ or double-blind method/ or matched-pair analysis/ or meta-analysis as topic/ or random allocation/ or single-blind method/ or “retraction of publication”/ or case reports/ (205801)

44 (random$ or placebo$ or single blind$ or double blind$ or triple blind$ or cohort$ or ((case$ or cohort or follow up or follow-up) adj2 (control$ or series or report$ or study or studies)) or retrospective$ or (observ$ adj3 (study or studies))).ti,ab. (572678)

45 meta-analysis/ or systematic reviews/ (41842)

46 qualitative analysis/ (2128)

47 clinical trials/ or randomized controlled trials/ (57929)

48 case-control studies/ or experimental design/ or retrospective studies/ or longitudinal studies/ or cohort studies/ or follow up/ or time series/ (101230)

49 or/38-48 (715947)

50 18 and 37 and 49 (17493)

51 limit 50 to yr=“2018 -Current” (**2912**)

1. **CINAHL (Ebsco) – Searched 26^th^ February 2020**

S41 S19 AND S30 AND S40 Limiters - Published Date: 20180201-20201231; Exclude MEDLINE records

Database - CINAHL Plus with Full Text **1,632**

S40 S31 OR S32 OR S33 OR S34 OR S35 OR S36 OR S37 OR S38 OR S39 Limiters - Published Date: 20180201-20201231

Database - CINAHL Plus with Full Text 143,470

S39 (MH “Random Assignment”) Limiters - Published Date: 20180201-20201231

Database - CINAHL Plus with Full Text 7,738

S38 (MH “Crossover Design”) OR (MH “Qualitative Studies+“) OR (MH “Quasi-Experimental Studies+“) OR (MH “Quantitative Studies”) OR (MH “Retrospective Design”) Limiters - Published Date: 20180201-20201231

Database - CINAHL Plus with Full Text 66,891

S37 (MH “Systematic Review”) Limiters - Published Date: 20180201-20201231

Database - CINAHL Plus with Full Text 17,614

S36 (MH “Meta Analysis”) Limiters - Published Date: 20180201-20201231

Database - CINAHL Plus with Full Text 9,381

S35 (MH “Controlled Before-After Studies”) OR (MH “Interrupted Time Series Analysis”) OR (MH “Pretest-Posttest Design”) OR (MH “Randomized Controlled Trials”) OR (MH “Clinical Trials”) OR (MH “Intervention Trials”) OR (MH “Double-Blind Studies”) OR (MH “Preventive Trials”) OR (MH “Community Trials”) Limiters - Published Date: 20180201-20201231

Database - CINAHL Plus with Full Text 32,692

S34 TI(“meta ethnograph*“ or “meta synthesis” or (synthesis and (“qualitative literature” or “qualitative research”)) or “critical interpretive synthesis” or (“systematic review” and (“qualitative research” or “qualitative literature” or “qualitative stud*“)) or “thematic synthesis” or “framework synthesis” or “realist review” or “realist synthesis” or “qualitative systematic review*“ or “qualitative evidence synthes*“ or ((“quality assessment” or “critical appraisal” or “literature search*“) and (“qualitative research” or “qualitative literature” or “qualitative stud*“)) or (Noblit and Hare) or “meta narrative*“ or “narrative synthesis”) OR AB(“meta ethnograph*“ or “meta synthesis” or (synthesis and (“qualitative literature” or “qualitative research”)) or “critical interpretive synthesis” or (“systematic review” and (“qualitative research” or “qualitative literature” or “qualitative stud*“)) or “thematic synthesis” or “framework synthesis” or “realist review” or “realist synthesis” or “qualitative systematic review*“ or “qualitative evidence synthes*“ or ((“quality assessment” or “critical appraisal” or “literature search*“) and (“qualitative research” or “qualitative literature” or “qualitative stud*“)) or (Noblit and Hare) or “meta narrative*“ or “narrative synthesis”) Limiters - Published Date: 20180201-20201231

Database - CINAHL Plus with Full Text 1,596

S33 TI(review N3 (effectiveness or effects or systemat* or synth* or integrat* or map* or methodologic* or quantitative or evidence or literature)) OR AB(review N3 (effectiveness or effects or systemat* or synth* or integrat* or map* or methodologic* or quantitative or evidence or literature)) Limiters - Published Date: 20180201-20201231

Database - CINAHL Plus with Full Text 37,760

S32 TI(“meta regression” or “meta synth*“ or “meta-synth*“ or “meta analy*“ or “metaanaly*“ or “meta-analy*“ or “metanaly*“ or “metaregression” or “metaregression” or “methodologic* overview” or “pool* analys*“ or “pool* data” or “quantitative* overview” or “research integration”) OR AB(“meta regression” or “meta synth*“ or “meta-synth*“ or “meta analy*“ or “metaanaly*“ or “meta-analy*“ or “metanaly*“ or “metaregression” or “metaregression” or “methodologic* overview” or “pool* analys*“ or “pool* data” or “quantitative* overview” or “research integration”) Limiters - Published Date: 20180201-20201231

Database - CINAHL Plus with Full Text 19,606

S31 TI((systematic* or synthes*) N3 (research or evaluation* or finding* or thematic* or report or descriptive or explanatory or narrative or meta* or review* or data or literature or studies or evidence or map or quantitative or study or studies or paper or impact or impacts or effect* or compar*)) OR AB((systematic* or synthes*) N3 (research or evaluation* or finding* or thematic* or report or descriptive or explanatory or narrative or meta* or review* or data or literature or studies or evidence or map or quantitative or study or studies or paper or impact or impacts or effect* or compar*)) Limiters - Published Date: 20180201-20201231

Database - CINAHL Plus with Full Text 29,586

S30 S20 OR S21 OR S22 OR S23 OR S24 OR S25 OR S26 OR S27 OR S28 OR S29 Limiters - Published Date: 20180201-20201231

Database - CINAHL Plus with Full Text 71,730

S29 TI (Africa or Asia or Caribbean or “West Indies” or “South America” or “Latin America” or “Central America”) OR AB (Africa or Asia or Caribbean or “West Indies” or “South America” or “Latin America” or “Central America”) OR SU (Africa or Asia or Caribbean or “West Indies” or “South America” or “Latin America” or “Central America”) OR GE (Africa or Asia or Caribbean or “West Indies” or “South America” or “Latin America” or “Central America”) Limiters - Published Date: 20180201-20201231

Database - CINAHL Plus with Full Text 9,582

S28 TI ( (“transitional country” or “transitional countries”)) OR AB ( (“transitional country” or “transitional countries”)) OR SU ( (“transitional country” or “transitional countries”)) Limiters - Published Date: 20180201-20201231

Database - CINAHL Plus with Full Text 8

S27 TI ( (lmic or lmics or “third world” or “lami country” or “lami countries”)) OR AB ( (lmic or lmics or “third world” or “lami country” or “lami countries”)) OR SU ( (lmic or lmics or “third world” or “lami country” or “lami countries”)) Limiters - Published Date: 20180201-20201231

Database - CINAHL Plus with Full Text 752

S26 TI (low N3 middle N3 countr*) OR AB (low N3 middle N3 countr*) OR SU (low N3 middle N3 countr*) Limiters - Published Date: 20180201-20201231

Database - CINAHL Plus with Full Text 2,772

S25 TI ( low* N1 (gdp or gnp or “gross domestic” or “gross national”)) OR AB ( low* N1 (gdp or gnp or “gross domestic” or “gross national”)) OR SU ( low* N1 (gdp or gnp or “gross domestic” or “gross national”)) Limiters - Published Date: 20180201-20201231

Database - CINAHL Plus with Full Text 11

S24 TI ( (developing or less* N1 developed or “under developed” or underdeveloped or “middle income” or low* N1 income) N1 (economy or economies)) OR AB ( (developing or less* N1 developed or “under developed” or underdeveloped or “middle income” or low* N1 income) N1 (economy or economies)) OR SU ( (developing or less* N1 developed or “under developed” or underdeveloped or “middle income” or low* N1 income) N1 (economy or economies)) Limiters - Published Date: 20180201-20201231

Database - CINAHL Plus with Full Text 29

S23 TI ( (developing or less* N1 developed or “under developed” or underdeveloped or “middle income” or low* N1 income or underserved or “under served” or deprived or poor*) N1 (countr* or nation* or population* or world)) OR AB ( (developing or less* N1 developed or “under developed” or underdeveloped or “middle income” or low* N1 income or underserved or “under served” or deprived or poor*) N1 (countr* or nation* or population* or world)) OR SU ( (developing or less* N1 developed or “under developed” or underdeveloped or “middle income” or low* N1 income or underserved or “under served” or deprived or poor*) N1 (countr* or nation* or population* or world)) Limiters - Published Date: 20180201-20201231

Database - CINAHL Plus with Full Text 6,976

S22 AB Afghanistan OR Albania OR Algeria OR Angola OR Antigua OR Barbuda OR Argentina OR Armenia OR Armenian OR Aruba OR Azerbaijan OR Bahrain OR Bangladesh OR Barbados OR Benin OR Belize OR Bhutan OR Bolivia OR Botswana OR Brazil OR Brasil OR “Burkina Faso” OR “Burkina Fasso” OR “Upper Volta” OR Burundi OR Urundi OR Cambodia OR “Khmer Republic” OR Kampuchea OR Cameroon OR Cameroons OR Cameron OR Camerons OR “Cape Verde” OR “Central African Republic” OR Chad OR Chile OR China OR Colombia OR Comoros OR “Comoro Islands” OR Comores OR Mayotte OR Congo OR Zaire OR “Costa Rica” OR “Cote d’Ivoire” OR “Ivory Coast” OR Cuba OR “Djibouti” OR “French Somaliland” OR Dominica OR “Dominican Republic” OR “East Timor” OR “East Timur” OR “Timor Leste” OR Ecuador OR Egypt OR “United Arab Republic” OR “El Salvador” OR Eritrea OR Ethiopia OR Fiji OR Gabon OR “Gabonese Republic” OR Gambia OR Gaza OR “Georgia Republic” OR “Georgian Republic” OR Ghana OR “Gold Coast” OR Grenada OR Guatemala OR Guinea OR Guam OR Guiana OR Guyana OR Haiti OR Honduras OR India OR Maldives OR Indonesia OR Iran OR Iraq OR Jamaica OR Jordan OR Kazakhstan OR Kazakh OR Kenya OR Kiribati OR Korea OR Kosovo OR Kyrgyzstan OR Kirghizia OR “Kyrgyz Republic” OR Kirghiz OR Kirgizstan OR “Lao PDR” OR Laos OR Lebanon OR Lesotho OR Basutoland OR Liberia OR Libya OR Madagascar OR “Malagasy Republic” OR Malaysia OR Malaya OR Malay OR Sabah OR Sarawak OR Malawi OR Nyasaland OR Mali OR “Marshall Islands” OR Mauritania OR Mauritius OR “Agalega Islands” OR Mexico OR Micronesia OR “Middle East” OR Moldova OR Moldovia OR Moldovian OR Mongolia OR Montenegro OR Morocco OR Ifni OR Mozambique OR Myanmar OR Myanma OR Burma OR Namibia OR Nepal OR Antilles OR “New Caledonia” OR Nicaragua OR Niger OR Nigeria OR “Mariana Islands” OR Oman OR Muscat OR Pakistan OR Palau OR Palestine OR Panama OR Paraguay OR Peru OR Philippines OR Philipines OR Phillipines OR Phillippines OR “Puerto Rico” OR Rwanda OR Ruanda OR “Saint Kitts” OR “St Kitts” OR Nevis OR “Saint Lucia” OR “St Lucia” OR “Saint Vincent” OR “St Vincent” OR “Grenadines” OR “Samoa” OR “Samoan Islands” OR “Navigator Island” OR “Navigator Islands” OR “Sao Tome” OR “Saudi Arabia” OR Senegal OR Seychelles OR “Sierra Leone” OR “Sri Lanka” OR “Solomon Islands” OR Somalia OR Sudan OR Suriname OR Surinam OR Swaziland OR Syria OR Tajikistan OR Tadzhikistan OR Tadjikistan OR Tadzhik OR Tanzania OR Thailand OR Togo OR “Togolese Republic” OR Tonga OR Trinidad OR Tobago OR Tunisia OR Turkey OR Turkmenistan OR Turkmen OR Uganda OR Ukraine OR Uruguay OR Uzbekistan OR Uzbek OR Vanuatu OR “New Hebrides” OR Venezuela OR Vietnam OR “Viet Nam” OR “West Bank” OR Yemen OR Zambia OR Zimbabwe OR Jamahiriya OR Jamahiryria OR Libia OR Mocambique OR Principe OR Syrian OR “Indian Ocean” OR Melanesia OR “Western Sahara” Limiters - Published Date: 20180201-20201231

Database - CINAHL Plus with Full Text 62,549

S21 TI Afghanistan OR Albania OR Algeria OR Angola OR Antigua OR Barbuda OR Argentina OR Armenia OR Armenian OR Aruba OR Azerbaijan OR Bahrain OR Bangladesh OR Barbados OR Benin OR Belize OR Bhutan OR Bolivia OR Botswana OR Brazil OR Brasil OR “Burkina Faso” OR “Burkina Fasso” OR “Upper Volta” OR Burundi OR Urundi OR Cambodia OR “Khmer Republic” OR Kampuchea OR Cameroon OR Cameroons OR Cameron OR Camerons OR “Cape Verde” OR “Central African Republic” OR Chad OR Chile OR China OR Colombia OR Comoros OR “Comoro Islands” OR Comores OR Mayotte OR Congo OR Zaire OR “Costa Rica” OR “Cote d’Ivoire” OR “Ivory Coast” OR Cuba OR “Djibouti” OR “French Somaliland” OR Dominica OR “Dominican Republic” OR “East Timor” OR “East Timur” OR “Timor Leste” OR Ecuador OR Egypt OR “United Arab Republic” OR “El Salvador” OR Eritrea OR Ethiopia OR Fiji OR Gabon OR “Gabonese Republic” OR Gambia OR Gaza OR “Georgia Republic” OR “Georgian Republic” OR Ghana OR “Gold Coast” OR Grenada OR Guatemala OR Guinea OR Guam OR Guiana OR Guyana OR Haiti OR Honduras OR India OR Maldives OR Indonesia OR Iran OR Iraq OR Jamaica OR Jordan OR Kazakhstan OR Kazakh OR Kenya OR Kiribati OR Korea OR Kosovo OR Kyrgyzstan OR Kirghizia OR “Kyrgyz Republic” OR Kirghiz OR Kirgizstan OR “Lao PDR” OR Laos OR Lebanon OR Lesotho OR Basutoland OR Liberia OR Libya OR Madagascar OR “Malagasy Republic” OR Malaysia OR Malaya OR Malay OR Sabah OR Sarawak OR Malawi OR Nyasaland OR Mali OR “Marshall Islands” OR Mauritania OR Mauritius OR “Agalega Islands” OR Mexico OR Micronesia OR “Middle East” OR Moldova OR Moldovia OR Moldovian OR Mongolia OR Montenegro OR Morocco OR Ifni OR Mozambique OR Myanmar OR Myanma OR Burma OR Namibia OR Nepal OR Antilles OR “New Caledonia” OR Nicaragua OR Niger OR Nigeria OR “Mariana Islands” OR Oman OR Muscat OR Pakistan OR Palau OR Palestine OR Panama OR Paraguay OR Peru OR Philippines OR Philipines OR Phillipines OR Phillippines OR “Puerto Rico” OR Rwanda OR Ruanda OR “Saint Kitts” OR “St Kitts” OR Nevis OR “Saint Lucia” OR “St Lucia” OR “Saint Vincent” OR “St Vincent” OR “Grenadines” OR “Samoa” OR “Samoan Islands” OR “Navigator Island” OR “Navigator Islands” OR “Sao Tome” OR “Saudi Arabia” OR Senegal OR Seychelles OR “Sierra Leone” OR “Sri Lanka” OR “Solomon Islands” OR Somalia OR Sudan OR Suriname OR Surinam OR Swaziland OR Syria OR Tajikistan OR Tadzhikistan OR Tadjikistan OR Tadzhik OR Tanzania OR Thailand OR Togo OR “Togolese Republic” OR Tonga OR Trinidad OR Tobago OR Tunisia OR Turkey OR Turkmenistan OR Turkmen OR Uganda OR Ukraine OR Uruguay OR Uzbekistan OR Uzbek OR Vanuatu OR “New Hebrides” OR Venezuela OR Vietnam OR “Viet Nam” OR “West Bank” OR Yemen OR Zambia OR Zimbabwe OR Jamahiriya OR Jamahiryria OR Libia OR Mocambique OR Principe OR Syrian OR “Indian Ocean” OR Melanesia OR “Western Sahara” Limiters - Published Date: 20180201-20201231

Database - CINAHL Plus with Full Text 62,515

S20 SU Afghanistan OR Albania OR Algeria OR Angola OR Antigua OR Barbuda OR Argentina OR Armenia OR Armenian OR Aruba OR Azerbaijan OR Bahrain OR Bangladesh OR Barbados OR Benin OR Belize OR Bhutan OR Bolivia OR Botswana OR Brazil OR Brasil OR “Burkina Faso” OR “Burkina Fasso” OR “Upper Volta” OR Burundi OR Urundi OR Cambodia OR “Khmer Republic” OR Kampuchea OR Cameroon OR Cameroons OR Cameron OR Camerons OR “Cape Verde” OR “Central African Republic” OR Chad OR Chile OR China OR Colombia OR Comoros OR “Comoro Islands” OR Comores OR Mayotte OR Congo OR Zaire OR “Costa Rica” OR “Cote d’Ivoire” OR “Ivory Coast” OR Cuba OR “Djibouti” OR “French Somaliland” OR Dominica OR “Dominican Republic” OR “East Timor” OR “East Timur” OR “Timor Leste” OR Ecuador OR Egypt OR “United Arab Republic” OR “El Salvador” OR Eritrea OR Ethiopia OR Fiji OR Gabon OR “Gabonese Republic” OR Gambia OR Gaza OR “Georgia Republic” OR “Georgian Republic” OR Ghana OR “Gold Coast” OR Grenada OR Guatemala OR Guinea OR Guam OR Guiana OR Guyana OR Haiti OR Honduras OR India OR Maldives OR Indonesia OR Iran OR Iraq OR Jamaica OR Jordan OR Kazakhstan OR Kazakh OR Kenya OR Kiribati OR Korea OR Kosovo OR Kyrgyzstan OR Kirghizia OR “Kyrgyz Republic” OR Kirghiz OR Kirgizstan OR “Lao PDR” OR Laos OR Lebanon OR Lesotho OR Basutoland OR Liberia OR Libya OR Madagascar OR “Malagasy Republic” OR Malaysia OR Malaya OR Malay OR Sabah OR Sarawak OR Malawi OR Nyasaland OR Mali OR “Marshall Islands” OR Mauritania OR Mauritius OR “Agalega Islands” OR Mexico OR Micronesia OR “Middle East” OR Moldova OR Moldovia OR Moldovian OR Mongolia OR Montenegro OR Morocco OR Ifni OR Mozambique OR Myanmar OR Myanma OR Burma OR Namibia OR Nepal OR Antilles OR “New Caledonia” OR Nicaragua OR Niger OR Nigeria OR “Mariana Islands” OR Oman OR Muscat OR Pakistan OR Palau OR Palestine OR Panama OR Paraguay OR Peru OR Philippines OR Philipines OR Phillipines OR Phillippines OR “Puerto Rico” OR Rwanda OR Ruanda OR “Saint Kitts” OR “St Kitts” OR Nevis OR “Saint Lucia” OR “St Lucia” OR “Saint Vincent” OR “St Vincent” OR “Grenadines” OR “Samoa” OR “Samoan Islands” OR “Navigator Island” OR “Navigator Islands” OR “Sao Tome” OR “Saudi Arabia” OR Senegal OR Seychelles OR “Sierra Leone” OR “Sri Lanka” OR “Solomon Islands” OR Somalia OR Sudan OR Suriname OR Surinam OR Swaziland OR Syria OR Tajikistan OR Tadzhikistan OR Tadjikistan OR Tadzhik OR Tanzania OR Thailand OR Togo OR “Togolese Republic” OR Tonga OR Trinidad OR Tobago OR Tunisia OR Turkey OR Turkmenistan OR Turkmen OR Uganda OR Ukraine OR Uruguay OR Uzbekistan OR Uzbek OR Vanuatu OR “New Hebrides” OR Venezuela OR Vietnam OR “Viet Nam” OR “West Bank” OR Yemen OR Zambia OR Zimbabwe OR Jamahiriya OR Jamahiryria OR Libia OR Mocambique OR Principe OR Syrian OR “Indian Ocean” OR Melanesia OR “Western Sahara” Limiters - Published Date: 20180201-20201231

Database - CINAHL Plus with Full Text 62,544

S19 S1 OR S2 OR S3 OR S4 OR S5 OR S6 OR S7 OR S8 OR S9 OR S10 OR S11 OR S12 OR S13 OR S14 OR S15 OR S16 OR S17 OR S18 Limiters - Published Date: 20180201-20201231

Database - CINAHL Plus with Full Text 71,585

S18 MM “Disabled+“) OR (MM “Child, Disabled”) OR (MM “Health Services for Persons with Disabilities”) OR (MM “Mentally Disabled Persons”) Limiters - Published Date: 20180201-20201231

Database - CINAHL Plus with Full Text 3,976

S17 (MM “Vision Disorders”) OR (MM “Blindness+“) OR (MH “Vision, Subnormal/ED/EP/PC/TH/RH”) Limiters - Published Date: 20180201-20201231

Database - CINAHL Plus with Full Text 1,088

S16 (MM “Hearing Disorders+/ED/EP/PC/RH/TH”) Limiters - Published Date: 20180201-20201231

Database - CINAHL Plus with Full Text 786

S15 (MM “Mental Disorders+/EP/ED/PC/RH/TH”) Limiters - Published Date: 20180201-20201231

Database - CINAHL Plus with Full Text 13,979

S14 TI(physical* N5 (impair* or deficien* or disable* or disabili* or handicap*)) OR AB(physical* N5 (impair* or deficien* or disable* or disabili* or handicap*)) Limiters - Published Date: 20180201-20201231

Database - CINAHL Plus with Full Text 1,736

S13 TI((“cerebral pals*“ or “spina bifida” or “muscular dystroph*“ or arthriti* or “osteogenesis imperfecta” or “musculoskeletal abnormalit*“ or “musculo-skeletal abnormalit*“ or “muscular abnormalit*“ or “skeletal abnormalit*“ or “limb abnormalit*“ or “brain injur*“ or amput* or clubfoot or polio* or paraplegi* or paralys* or paralyz* or hemiplegi* or stroke* or “cerebrovascular accident*“) N2 (impair* or disabilit* or disabl* or handicap*)) OR AB((“cerebral pals*“ or “spina bifida” or “muscular dystroph*“ or arthriti* or “osteogenesis imperfecta” or “musculoskeletal abnormalit*“ or “musculo-skeletal abnormalit*“ or “muscular abnormalit*“ or “skeletal abnormalit*“ or “limb abnormalit*“ or “brain injur*“ or amput* or clubfoot or polio* or paraplegi* or paralys* or paralyz* or hemiplegi* or stroke* or “cerebrovascular accident*“) N2 (impair* or disabilit* or disabl* or handicap*)) Limiters - Published Date: 20180201-20201231

Database - CINAHL Plus with Full Text 583

S12 TI(((visual* or vision or eye* or ocular) N5 (loss* or impair* or deficien* or disable* or disabili* or handicap*)) or blind*) OR AB(((visual* or vision or eye* or ocular) N5 (loss* or impair* or deficien* or disable* or disabili* or handicap*)) or blind*) Limiters - Published Date: 20180201-20201231

Database - CINAHL Plus with Full Text 14,417

S11 TI(((hearing or acoustic or ear*) N5 (loss* or impair* or deficien* or disable* or disabili* or handicap*)) or deaf*) OR AB(((hearing or acoustic or ear*) N5 (loss* or impair* or deficien* or disable* or disabili* or handicap*)) or deaf*) Limiters - Published Date: 20180201-20201231

Database - CINAHL Plus with Full Text 5,154

S10 TI((intellectual* or educational* or mental* or psychological* or developmental) N5 (impair* or retard* or deficien* or disable* or disabili* or handicap* or ill*)) OR AB((intellectual* or educational* or mental* or psychological* or developmental) N5 (impair* or retard* or deficien* or disable* or disabili* or handicap* or ill*)) Limiters - Published Date: 20180201-20201231

Database - CINAHL Plus with Full Text 7,736

S9 TI(autis* or dyslexi* or “Down* syndrome” or mongolism or “trisomy 21”) OR AB(autis* or dyslexi* or “Down* syndrome” or mongolism or “trisomy 21”) Limiters - Published Date: 20180201-20201231

Database - CINAHL Plus with Full Text 5,411

S8 TI((mental* or emotional* or psychiatric or neurologic*) N2 (disorder* or ill or illness*)) OR AB((mental* or emotional* or psychiatric or neurologic*) N2 (disorder* or ill or illness*)) Limiters - Published Date: 20180201-20201231

Database - CINAHL Plus with Full Text 9,379

S7 TI((schizophreni* or psychos* or psychotic or schizoaffective or schizophreniform or dementia* or alzheimer*) N2 (impair* or disabilit* or disabl* or handicap*)) OR AB((schizophreni* or psychos* or psychotic or schizoaffective or schizophreniform or dementia* or alzheimer*) N2 (impair* or disabilit* or disabl* or handicap*)) Limiters - Published Date: 20180201-20201231

Database - CINAHL Plus with Full Text 1,140

S6 TI(“mental health”) OR AB(“mental health”) Limiters - Published Date: 20180201-20201231

Database - CINAHL Plus with Full Text 17,746

S5 TI((depression or depressive or anxiety or psychiat* or well-being or “quality of life” or self-esteem or “self perception”) N2 (impair* or disabilit* or disabl* or handicap*)) OR AB((depression or depressive or anxiety or psychiat* or well-being or “quality of life” or self-esteem or “self perception”) N2 (impair* or disabilit* or disabl* or handicap*) Limiters - Published Date: 20180201-20201231

Database - CINAHL Plus with Full Text 1,511

S4 TI((communication or language or speech or learning) N5 disorder*) OR AB((communication or language or speech or learning) N5 disorder*) Limiters - Published Date: 20180201-20201231

Database - CINAHL Plus with Full Text 1,107

S3 TI((cognitive* or learning or mobility or sensory or visual* or vision or sight or hearing or physical* or mental* or intellectual*) N2 (impair* or disabilit* or disabl* or handicap*)) OR AB((cognitive* or learning or mobility or sensory or visual* or vision or sight or hearing or physical* or mental* or intellectual*) N2 (impair* or disabilit* or disabl* or handicap*)) Limiters - Published Date: 20180201-20201231

Database - CINAHL Plus with Full Text 11,556

S2 TI((physical* or intellectual* or learning or psychiatric* or sensory or motor or neuromotor or cognitive or mental* or developmental or communication or learning) N2 (disabilit* or disabl* or handicap*)) OR AB ((physical* or intellectual* or learning or psychiatric* or sensory or motor or neuromotor or cognitive or mental* or developmental or communication or learning) N2 (disabilit* or disabl* or handicap*)) Limiters - Published Date: 20180201-20201231

Database - CINAHL Plus with Full Text 4,472

S1 TI((disable* or disabilit* or handicapped) N5 (person* or people or child* or adolescen* or women or mother* or maternal or group*)) OR AB ((disable* or disabilit* or handicapped) N5 (person* or people or child* or adolescen* or women or mother* or maternal or group*)) Limiters - Published Date: 20180201-20201231

Database - CINAHL Plus with Full Text 4,528

1. **ERIC (Ebsco) – Searched 26^th^ February 2020**

S34 S16 AND S27 AND S33

Database - ERIC 83

S33 S28 OR S29 OR S30 OR S31 OR S32 Limiters - Date Published: 20180201-20201231

Database - ERIC 5,619

S32 (DE “Meta Analysis” OR DE “Multiple Regression Analysis” OR DE “Mixed Methods Research” OR DE “Randomized Controlled Trials” OR DE “Evaluation Methods” OR DE “Qualitative Research” OR DE “Quasiexperimental Design” OR DE “Control Groups” OR DE “Matched Groups” OR DE “Pretests Posttests”) Limiters - Date Published: 20180201-20201231

Database - ERIC 3,695

S31 TI(“meta ethnograph*“ or “meta synthesis” or (synthesis and (“qualitative literature” or “qualitative research”)) or “critical interpretive synthesis” or (“systematic review” and (“qualitative research” or “qualitative literature” or “qualitative stud*“)) or “thematic synthesis” or “framework synthesis” or “realist review” or “realist synthesis” or “qualitative systematic review*“ or “qualitative evidence synthes*“ or ((“quality assessment” or “critical appraisal” or “literature search*“) and (“qualitative research” or “qualitative literature” or “qualitative stud*“)) or (Noblit and Hare) or “meta narrative*“ or “narrative synthesis”) OR AB(“meta ethnograph*“ or “meta synthesis” or (synthesis and (“qualitative literature” or “qualitative research”)) or “critical interpretive synthesis” or (“systematic review” and (“qualitative research” or “qualitative literature” or “qualitative stud*“)) or “thematic synthesis” or “framework synthesis” or “realist review” or “realist synthesis” or “qualitative systematic review*“ or “qualitative evidence synthes*“ or ((“quality assessment” or “critical appraisal” or “literature search*“) and (“qualitative research” or “qualitative literature” or “qualitative stud*“)) or (Noblit and Hare) or “meta narrative*“ or “narrative synthesis”) Limiters - Date Published: 20180201-20201231

Database - ERIC 67

S30 TI(review N3 (effectiveness or effects or systemat* or synth* or integrat* or map* or methodologic* or quantitative or evidence or literature)) OR AB(review N3 (effectiveness or effects or systemat* or synth* or integrat* or map* or methodologic* or quantitative or evidence or literature)) Limiters - Date Published: 20180201-20201231

Database - ERIC 1,487

S29 TI(“meta regression” or “meta synth*“ or “meta-synth*“ or “meta analy*“ or “metaanaly*“ or “meta-analy*“ or “metanaly*“ or “metaregression” or “metaregression” or “methodologic* overview” or “pool* analys*“ or “pool* data” or “quantitative* overview” or “research integration”) OR AB(“meta regression” or “meta synth*“ or “meta-synth*“ or “meta analy*“ or “metaanaly*“ or “meta-analy*“ or “metanaly*“ or “metaregression” or “metaregression” or “methodologic* overview” or “pool* analys*“ or “pool* data” or “quantitative* overview” or “research integration”) Limiters - Date Published: 20180201-20201231

Database - ERIC 396

S28 TI((systematic* or synthes*) N3 (research or evaluation* or finding* or thematic* or report or descriptive or explanatory or narrative or meta* or review* or data or literature or studies or evidence or map or quantitative or study or studies or paper or impact or impacts or effect* or compar*)) OR AB((systematic* or synthes*) N3 (research or evaluation* or finding* or thematic* or report or descriptive or explanatory or narrative or meta* or review* or data or literature or studies or evidence or map or quantitative or study or studies or paper or impact or impacts or effect* or compar*)) Limiters - Date Published: 20180201-20201231

Database - ERIC 1,205

S27 S17 OR S18 OR S19 OR S20 OR S21 OR S22 OR S23 OR S24 OR S25 OR S26 Limiters - Date Published: 20180201-20201231

Database - ERIC 10,382

S26 TI (Africa or Asia or Caribbean or “West Indies” or “South America” or “Latin America” or “Central America”) OR AB (Africa or Asia or Caribbean or “West Indies” or “South America” or “Latin America” or “Central America”) OR SU (Africa or Asia or Caribbean or “West Indies” or “South America” or “Latin America” or “Central America”) OR GE (Africa or Asia or Caribbean or “West Indies” or “South America” or “Latin America” or “Central America”) Limiters - Date Published: 20180201-20201231

Database - ERIC 1,074

S25 TI ( (“transitional country” or “transitional countries”)) OR AB ( (“transitional country” or “transitional countries”)) OR SU ( (“transitional country” or “transitional countries”)) Limiters - Date Published: 20180201-20201231

Database - ERIC 2

S24 TI ( (lmic or lmics or “third world” or “lami country” or “lami countries”)) OR AB ( (lmic or lmics or “third world” or “lami country” or “lami countries”)) OR SU ( (lmic or lmics or “third world” or “lami country” or “lami countries”)) Limiters - Date Published: 20180201-20201231

Database - ERIC 18

S23 TI (low N3 middle N3 countr*) OR AB (low N3 middle N3 countr*) OR SU (low N3 middle N3 countr*) Limiters - Date Published: 20180201-20201231

Database - ERIC 52

S22 TI ( low* N1 (gdp or gnp or “gross domestic” or “gross national”)) OR AB ( low* N1 (gdp or gnp or “gross domestic” or “gross national”)) OR SU ( low* N1 (gdp or gnp or “gross domestic” or “gross national”)) Limiters - Date Published: 20180201-20201231

Database - ERIC 2

S21 TI ( (developing or less* N1 developed or “under developed” or underdeveloped or “middle income” or low* N1 income) N1 (economy or economies)) OR AB ( (developing or less* N1 developed or “under developed” or underdeveloped or “middle income” or low* N1 income) N1 (economy or economies)) OR SU ( (developing or less* N1 developed or “under developed” or underdeveloped or “middle income” or low* N1 income) N1 (economy or economies)) Limiters - Date Published: 20180201-20201231

Database - ERIC 12

S20 TI ( (developing or less* N1 developed or “under developed” or underdeveloped or “middle income” or low* N1 income or underserved or “under served” or deprived or poor*) N1 (countr* or nation* or population* or world)) OR AB ( (developing or less* N1 developed or “under developed” or underdeveloped or “middle income” or low* N1 income or underserved or “under served” or deprived or poor*) N1 (countr* or nation* or population* or world)) OR SU ( (developing or less* N1 developed or “under developed” or underdeveloped or “middle income” or low* N1 income or underserved or “under served” or deprived or poor*) N1 (countr* or nation* or population* or world)) Limiters - Date Published: 20180201-20201231

Database - ERIC 447

S19 AB Afghanistan OR Albania OR Algeria OR Angola OR Antigua OR Barbuda OR Argentina OR Armenia OR Armenian OR Aruba OR Azerbaijan OR Bahrain OR Bangladesh OR Barbados OR Benin OR Belize OR Bhutan OR Bolivia OR Botswana OR Brazil OR Brasil OR “Burkina Faso” OR “Burkina Fasso” OR “Upper Volta” OR Burundi OR Urundi OR Cambodia OR “Khmer Republic” OR Kampuchea OR Cameroon OR Cameroons OR Cameron OR Camerons OR “Cape Verde” OR “Central African Republic” OR Chad OR Chile OR China OR Colombia OR Comoros OR “Comoro Islands” OR Comores OR Mayotte OR Congo OR Zaire OR “Costa Rica” OR “Cote d’Ivoire” OR “Ivory Coast” OR Cuba OR “Djibouti” OR “French Somaliland” OR Dominica OR “Dominican Republic” OR “East Timor” OR “East Timur” OR “Timor Leste” OR Ecuador OR Egypt OR “United Arab Republic” OR “El Salvador” OR Eritrea OR Ethiopia OR Fiji OR Gabon OR “Gabonese Republic” OR Gambia OR Gaza OR “Georgia Republic” OR “Georgian Republic” OR Ghana OR “Gold Coast” OR Grenada OR Guatemala OR Guinea OR Guam OR Guiana OR Guyana OR Haiti OR Honduras OR India OR Maldives OR Indonesia OR Iran OR Iraq OR Jamaica OR Jordan OR Kazakhstan OR Kazakh OR Kenya OR Kiribati OR Korea OR Kosovo OR Kyrgyzstan OR Kirghizia OR “Kyrgyz Republic” OR Kirghiz OR Kirgizstan OR “Lao PDR” OR Laos OR Lebanon OR Lesotho OR Basutoland OR Liberia OR Libya OR Madagascar OR “Malagasy Republic” OR Malaysia OR Malaya OR Malay OR Sabah OR Sarawak OR Malawi OR Nyasaland OR Mali OR “Marshall Islands” OR Mauritania OR Mauritius OR “Agalega Islands” OR Mexico OR Micronesia OR “Middle East” OR Moldova OR Moldovia OR Moldovian OR Mongolia OR Montenegro OR Morocco OR Ifni OR Mozambique OR Myanmar OR Myanma OR Burma OR Namibia OR Nepal OR Antilles OR “New Caledonia” OR Nicaragua OR Niger OR Nigeria OR “Mariana Islands” OR Oman OR Muscat OR Pakistan OR Palau OR Palestine OR Panama OR Paraguay OR Peru OR Philippines OR Philipines OR Phillipines OR Phillippines OR “Puerto Rico” OR Rwanda OR Ruanda OR “Saint Kitts” OR “St Kitts” OR Nevis OR “Saint Lucia” OR “St Lucia” OR “Saint Vincent” OR “St Vincent” OR “Grenadines” OR “Samoa” OR “Samoan Islands” OR “Navigator Island” OR “Navigator Islands” OR “Sao Tome” OR “Saudi Arabia” OR Senegal OR Seychelles OR “Sierra Leone” OR “Sri Lanka” OR “Solomon Islands” OR Somalia OR Sudan OR Suriname OR Surinam OR Swaziland OR Syria OR Tajikistan OR Tadzhikistan OR Tadjikistan OR Tadzhik OR Tanzania OR Thailand OR Togo OR “Togolese Republic” OR Tonga OR Trinidad OR Tobago OR Tunisia OR Turkey OR Turkmenistan OR Turkmen OR Uganda OR Ukraine OR Uruguay OR Uzbekistan OR Uzbek OR Vanuatu OR “New Hebrides” OR Venezuela OR Vietnam OR “Viet Nam” OR “West Bank” OR Yemen OR Zambia OR Zimbabwe OR Jamahiriya OR Jamahiryria OR Libia OR Mocambique OR Principe OR Syrian OR “Indian Ocean” OR Melanesia OR “Western Sahara” Limiters - Date Published: 20180201-20201231

Database - ERIC 9,570

S18 TI Afghanistan OR Albania OR Algeria OR Angola OR Antigua OR Barbuda OR Argentina OR Armenia OR Armenian OR Aruba OR Azerbaijan OR Bahrain OR Bangladesh OR Barbados OR Benin OR Belize OR Bhutan OR Bolivia OR Botswana OR Brazil OR Brasil OR “Burkina Faso” OR “Burkina Fasso” OR “Upper Volta” OR Burundi OR Urundi OR Cambodia OR “Khmer Republic” OR Kampuchea OR Cameroon OR Cameroons OR Cameron OR Camerons OR “Cape Verde” OR “Central African Republic” OR Chad OR Chile OR China OR Colombia OR Comoros OR “Comoro Islands” OR Comores OR Mayotte OR Congo OR Zaire OR “Costa Rica” OR “Cote d’Ivoire” OR “Ivory Coast” OR Cuba OR “Djibouti” OR “French Somaliland” OR Dominica OR “Dominican Republic” OR “East Timor” OR “East Timur” OR “Timor Leste” OR Ecuador OR Egypt OR “United Arab Republic” OR “El Salvador” OR Eritrea OR Ethiopia OR Fiji OR Gabon OR “Gabonese Republic” OR Gambia OR Gaza OR “Georgia Republic” OR “Georgian Republic” OR Ghana OR “Gold Coast” OR Grenada OR Guatemala OR Guinea OR Guam OR Guiana OR Guyana OR Haiti OR Honduras OR India OR Maldives OR Indonesia OR Iran OR Iraq OR Jamaica OR Jordan OR Kazakhstan OR Kazakh OR Kenya OR Kiribati OR Korea OR Kosovo OR Kyrgyzstan OR Kirghizia OR “Kyrgyz Republic” OR Kirghiz OR Kirgizstan OR “Lao PDR” OR Laos OR Lebanon OR Lesotho OR Basutoland OR Liberia OR Libya OR Madagascar OR “Malagasy Republic” OR Malaysia OR Malaya OR Malay OR Sabah OR Sarawak OR Malawi OR Nyasaland OR Mali OR “Marshall Islands” OR Mauritania OR Mauritius OR “Agalega Islands” OR Mexico OR Micronesia OR “Middle East” OR Moldova OR Moldovia OR Moldovian OR Mongolia OR Montenegro OR Morocco OR Ifni OR Mozambique OR Myanmar OR Myanma OR Burma OR Namibia OR Nepal OR Antilles OR “New Caledonia” OR Nicaragua OR Niger OR Nigeria OR “Mariana Islands” OR Oman OR Muscat OR Pakistan OR Palau OR Palestine OR Panama OR Paraguay OR Peru OR Philippines OR Philipines OR Phillipines OR Phillippines OR “Puerto Rico” OR Rwanda OR Ruanda OR “Saint Kitts” OR “St Kitts” OR Nevis OR “Saint Lucia” OR “St Lucia” OR “Saint Vincent” OR “St Vincent” OR “Grenadines” OR “Samoa” OR “Samoan Islands” OR “Navigator Island” OR “Navigator Islands” OR “Sao Tome” OR “Saudi Arabia” OR Senegal OR Seychelles OR “Sierra Leone” OR “Sri Lanka” OR “Solomon Islands” OR Somalia OR Sudan OR Suriname OR Surinam OR Swaziland OR Syria OR Tajikistan OR Tadzhikistan OR Tadjikistan OR Tadzhik OR Tanzania OR Thailand OR Togo OR “Togolese Republic” OR Tonga OR Trinidad OR Tobago OR Tunisia OR Turkey OR Turkmenistan OR Turkmen OR Uganda OR Ukraine OR Uruguay OR Uzbekistan OR Uzbek OR Vanuatu OR “New Hebrides” OR Venezuela OR Vietnam OR “Viet Nam” OR “West Bank” OR Yemen OR Zambia OR Zimbabwe OR Jamahiriya OR Jamahiryria OR Libia OR Mocambique OR Principe OR Syrian OR “Indian Ocean” OR Melanesia OR “Western Sahara” Limiters - Date Published: 20180201-20201231

Database - ERIC 9,569

S17 SU Afghanistan OR Albania OR Algeria OR Angola OR Antigua OR Barbuda OR Argentina OR Armenia OR Armenian OR Aruba OR Azerbaijan OR Bahrain OR Bangladesh OR Barbados OR Benin OR Belize OR Bhutan OR Bolivia OR Botswana OR Brazil OR Brasil OR “Burkina Faso” OR “Burkina Fasso” OR “Upper Volta” OR Burundi OR Urundi OR Cambodia OR “Khmer Republic” OR Kampuchea OR Cameroon OR Cameroons OR Cameron OR Camerons OR “Cape Verde” OR “Central African Republic” OR Chad OR Chile OR China OR Colombia OR Comoros OR “Comoro Islands” OR Comores OR Mayotte OR Congo OR Zaire OR “Costa Rica” OR “Cote d’Ivoire” OR “Ivory Coast” OR Cuba OR “Djibouti” OR “French Somaliland” OR Dominica OR “Dominican Republic” OR “East Timor” OR “East Timur” OR “Timor Leste” OR Ecuador OR Egypt OR “United Arab Republic” OR “El Salvador” OR Eritrea OR Ethiopia OR Fiji OR Gabon OR “Gabonese Republic” OR Gambia OR Gaza OR “Georgia Republic” OR “Georgian Republic” OR Ghana OR “Gold Coast” OR Grenada OR Guatemala OR Guinea OR Guam OR Guiana OR Guyana OR Haiti OR Honduras OR India OR Maldives OR Indonesia OR Iran OR Iraq OR Jamaica OR Jordan OR Kazakhstan OR Kazakh OR Kenya OR Kiribati OR Korea OR Kosovo OR Kyrgyzstan OR Kirghizia OR “Kyrgyz Republic” OR Kirghiz OR Kirgizstan OR “Lao PDR” OR Laos OR Lebanon OR Lesotho OR Basutoland OR Liberia OR Libya OR Madagascar OR “Malagasy Republic” OR Malaysia OR Malaya OR Malay OR Sabah OR Sarawak OR Malawi OR Nyasaland OR Mali OR “Marshall Islands” OR Mauritania OR Mauritius OR “Agalega Islands” OR Mexico OR Micronesia OR “Middle East” OR Moldova OR Moldovia OR Moldovian OR Mongolia OR Montenegro OR Morocco OR Ifni OR Mozambique OR Myanmar OR Myanma OR Burma OR Namibia OR Nepal OR Antilles OR “New Caledonia” OR Nicaragua OR Niger OR Nigeria OR “Mariana Islands” OR Oman OR Muscat OR Pakistan OR Palau OR Palestine OR Panama OR Paraguay OR Peru OR Philippines OR Philipines OR Phillipines OR Phillippines OR “Puerto Rico” OR Rwanda OR Ruanda OR “Saint Kitts” OR “St Kitts” OR Nevis OR “Saint Lucia” OR “St Lucia” OR “Saint Vincent” OR “St Vincent” OR “Grenadines” OR “Samoa” OR “Samoan Islands” OR “Navigator Island” OR “Navigator Islands” OR “Sao Tome” OR “Saudi Arabia” OR Senegal OR Seychelles OR “Sierra Leone” OR “Sri Lanka” OR “Solomon Islands” OR Somalia OR Sudan OR Suriname OR Surinam OR Swaziland OR Syria OR Tajikistan OR Tadzhikistan OR Tadjikistan OR Tadzhik OR Tanzania OR Thailand OR Togo OR “Togolese Republic” OR Tonga OR Trinidad OR Tobago OR Tunisia OR Turkey OR Turkmenistan OR Turkmen OR Uganda OR Ukraine OR Uruguay OR Uzbekistan OR Uzbek OR Vanuatu OR “New Hebrides” OR Venezuela OR Vietnam OR “Viet Nam” OR “West Bank” OR Yemen OR Zambia OR Zimbabwe OR Jamahiriya OR Jamahiryria OR Libia OR Mocambique OR Principe OR Syrian OR “Indian Ocean” OR Melanesia OR “Western Sahara” Limiters - Date Published: 20180201-20201231

Database - ERIC 9,572

S16 S1 OR S2 OR S3 OR S4 OR S5 OR S6 OR S7 OR S8 OR S9 OR S10 OR S11 OR S12 OR S13 OR S14 OR S15 Limiters - Date Published: 20180201-20201231

Database - ERIC 5,429

S15 (DE “Intellectual Disability” OR DE “Down Syndrome” OR DE “Mild Intellectual Disability” OR DE “Moderate Intellectual Disability” OR DE “Severe Intellectual Disability” OR DE “Severe Intellectual Disability” OR DE “Moderate Intellectual Disability” OR DE “Mild Intellectual Disability” OR DE “Severity (of Disability)” OR DE “Disability Identification” OR DE “Disability Discrimination” OR DE “Developmental Disabilities” OR DE “Learning Disabilities” OR DE “Mental Health” OR DE “Mental Disorders” OR DE “Anxiety Disorders” OR DE “Dementia” OR DE “Emotional Disturbances” OR DE “Neurosis” OR DE “Pervasive Developmental Disorders” OR DE “Psychosis” OR DE “Mental Health Programs” OR DE “Disability Identification” OR DE “Deafness” OR DE “Deaf Blind” OR DE “Autism” OR DE “Asperger Syndrome” OR DE “Partial Vision” OR DE “Blindness” OR DE “Depression (Psychology)” OR DE “Speech Impairments” OR DE “Articulation Impairments” OR DE “Delayed Speech” OR DE “Stuttering” OR DE “Voice Disorders” OR DE “Stuttering”) Limiters - Date Published: 20180201-20201231

Database - ERIC 4,343

S14 TI(physical* N5 (impair* or deficien* or disable* or disabili* or handicap*)) OR AB(physical* N5 (impair* or deficien* or disable* or disabili* or handicap*)) Limiters - Date Published: 20180201-20201231

Database - ERIC 91

S13 TI((“cerebral pals*“ or “spina bifida” or “muscular dystroph*“ or arthriti* or “osteogenesis imperfecta” or “musculoskeletal abnormalit*“ or “musculo-skeletal abnormalit*“ or “muscular abnormalit*“ or “skeletal abnormalit*“ or “limb abnormalit*“ or “brain injur*“ or amput* or clubfoot or polio* or paraplegi* or paralys* or paralyz* or hemiplegi* or stroke* or “cerebrovascular accident*“) N2 (impair* or disabilit* or disabl* or handicap*)) OR AB((“cerebral pals*“ or “spina bifida” or “muscular dystroph*“ or arthriti* or “osteogenesis imperfecta” or “musculoskeletal abnormalit*“ or “musculo-skeletal abnormalit*“ or “muscular abnormalit*“ or “skeletal abnormalit*“ or “limb abnormalit*“ or “brain injur*“ or amput* or clubfoot or polio* or paraplegi* or paralys* or paralyz* or hemiplegi* or stroke* or “cerebrovascular accident*“) N2 (impair* or disabilit* or disabl* or handicap*)) Limiters - Date Published: 20180201-20201231

Database - ERIC 7

S12 TI(((visual* or vision or eye* or ocular) N5 (loss* or impair* or deficien* or disable* or disabili* or handicap*)) or blind*) OR AB(((visual* or vision or eye* or ocular) N5 (loss* or impair* or deficien* or disable* or disabili* or handicap*)) or blind*) Limiters - Date Published: 20180201-20201231

Database - ERIC 261

S11 TI(((hearing or acoustic or ear*) N5 (loss* or impair* or deficien* or disable* or disabili* or handicap*)) or deaf*) OR AB(((hearing or acoustic or ear*) N5 (loss* or impair* or deficien* or disable* or disabili* or handicap*)) or deaf*) Limiters - Date Published: 20180201-20201231

Database - ERIC 366

S10 TI((intellectual* or educational* or mental* or psychological* or developmental) N5 (impair* or retard* or deficien* or disable* or disabili* or handicap* or ill*)) OR AB((intellectual* or educational* or mental* or psychological* or developmental) N5 (impair* or retard* or deficien* or disable* or disabili* or handicap* or ill*)) Limiters - Date Published: 20180201-20201231

Database - ERIC 1,013

S9 TI(autis* or dyslexi* or “Down* syndrome” or mongolism or “trisomy 21”) OR AB(autis* or dyslexi* or “Down* syndrome” or mongolism or “trisomy 21”) Limiters - Date Published: 20180201-20201231

Database - ERIC 1,766

S8 TI((mental* or emotional* or psychiatric or neurologic*) N2 (disorder* or ill or illness*)) OR AB((mental* or emotional* or psychiatric or neurologic*) N2 (disorder* or ill or illness*)) Limiters - Date Published: 20180201-20201231

Database - ERIC 254

S7 TI((schizophreni* or psychos* or psychotic or schizoaffective or schizophreniform or dementia* or alzheimer*) N2 (impair* or disabilit* or disabl* or handicap*)) OR AB((schizophreni* or psychos* or psychotic or schizoaffective or schizophreniform or dementia* or alzheimer*) N2 (impair* or disabilit* or disabl* or handicap*)) Limiters - Date Published: 20180201-20201231

Database - ERIC 5

S6 TI(“mental health”) OR AB(“mental health”) Limiters - Date Published: 20180201-20201231

Database - ERIC 721

S5 TI((depression or depressive or anxiety or psychiat* or well-being or “quality of life” or self-esteem or “self perception”) N2 (impair* or disabilit* or disabl* or handicap*)) OR AB((depression or depressive or anxiety or psychiat* or well-being or “quality of life” or self-esteem or “self perception”) N2 (impair* or disabilit* or disabl* or handicap*) Limiters - Date Published: 20180201-20201231

Database - ERIC 31

S4 TI((communication or language or speech or learning) N5 disorder*) OR AB((communication or language or speech or learning) N5 disorder*) Limiters - Date Published: 20180201-20201231

Database - ERIC 275

S3 TI((cognitive* or learning or mobility or sensory or visual* or vision or sight or hearing or physical* or mental* or intellectual*) N2 (impair* or disabilit* or disabl* or handicap*)) OR AB((cognitive* or learning or mobility or sensory or visual* or vision or sight or hearing or physical* or mental* or intellectual*) N2 (impair* or disabilit* or disabl* or handicap*)) Limiters - Date Published: 20180201-20201231

Database - ERIC 1,222

S2 TI((physical* or intellectual* or learning or psychiatric* or sensory or motor or neuromotor or cognitive or mental* or developmental or communication or learning) N2 (disabilit* or disabl* or handicap*)) OR AB ((physical* or intellectual* or learning or psychiatric* or sensory or motor or neuromotor or cognitive or mental* or developmental or communication or learning) N2 (disabilit* or disabl* or handicap*)) Limiters - Date Published: 20180201-20201231

Database - ERIC 1,123

S1 TI((disable* or disabilit* or handicapped) N5 (person* or people or child* or adolescen* or women or mother* or maternal or group*)) OR AB ((disable* or disabilit* or handicapped) N5 (person* or people or child* or adolescen* or women or mother* or maternal or group*)) Limiters - Date Published: 20180201-20201231

Database - ERIC **889**

1. **Scopus – Searched 26^th^ February 2020**

( ( TITLE ( ( ( intellectual* OR educational* OR mental* OR psychological* OR developmental) W/5 ( impair* OR retard* OR deficien* OR disable* OR disabili* OR handicap* OR ill*)))) OR ( TITLE ( ( ( hearing OR acoustic OR ear*) W/5 ( loss* OR impair* OR deficien* OR disable* OR disabili* OR handicap*)) OR deaf*)) OR ( TITLE ( ( ( ( visual* OR vision OR eye* OR ocular) W/5 ( loss* OR impair* OR deficien* OR disable* OR disabili* OR handicap*)) OR blind*))) OR ( TITLE ( ( ( “cerebral pals*“ OR “spina bifida” OR “muscular dystroph*“ OR arthriti* OR “osteogenesis imperfecta” OR “musculoskeletal abnormalit*“ OR “musculo-skeletal abnormalit*“ OR “muscular abnormalit*“ OR “skeletal abnormalit*“ OR “limb abnormalit*“ OR “brain injur*“ OR amput* OR clubfoot OR polio* OR paraplegi* OR paralys* OR paralyz* OR hemiplegi* OR stroke* OR “cerebrovascular accident*“) W/2 ( impair* OR disabilit* OR disabl* OR handicap*)))) OR ( TITLE ( ( physical* W/5 ( impair* OR deficien* OR disable* OR disabili* OR handicap*)))) OR ( ( TITLE ( ( ( disable* OR disabilit* OR handicapped) W/5 ( person* OR people OR child* OR adolescen* OR women OR mother* OR maternal OR group*)))) OR ( TITLE ( ( ( physical* OR intellectual* OR learning OR psychiatric* OR sensory OR motor OR neuromotor OR cognitive OR mental* OR developmental OR communication OR learning) W/2 ( disabilit* OR disabl* OR handicap*)))) OR ( TITLE ( ( ( cognitive* OR learning OR mobility OR sensory OR visual* OR vision OR sight OR hearing OR physical* OR mental* OR intellectual*) W/2 ( impair* OR disabilit* OR disabl* OR handicap*)))) OR ( TITLE ( ( ( communication OR language OR speech OR learning) W/5 disorder*))) OR ( TITLE ( ( ( depression OR depressive OR anxiety OR psychiat* OR well-being OR “quality of life” OR self-esteem OR “self perception”) W/2 ( impair* OR disabilit* OR disabl* OR handicap*)))) OR ( TITLE ( “mental health”)) OR ( TITLE ( ( ( schizophreni* OR psychos* OR psychotic OR schizoaffective OR schizophreniform OR dementia* OR alzheimer*) W/2 ( impair* OR disabilit* OR disabl* OR handicap*)))) OR ( TITLE ( ( ( mental* OR emotional* OR psychiatric OR neurologic*) W/2 ( disorder* OR ill OR illness*)))) OR ( TITLE ( ( autis* OR dyslexi* OR “Down* syndrome” OR mongolism OR “trisomy 21”))))) AND ( TITLE-ABS-KEY ( afghanistan OR albania OR algeria OR angola OR argentina OR armenia OR armenian OR aruba OR azerbaijan OR bangladesh OR benin OR byelarus OR byelorussian OR belarus OR belorussian OR belorussia OR belize OR bhutan OR bolivia OR bosnia OR herzegovina OR hercegovina OR botswana OR brasil OR brazil OR bulgaria OR “Burkina Faso” OR “Burkina Fasso” OR “Upper Volta” OR burundi OR urundi OR cambodia OR “Khmer Republic” OR kampuchea OR cameroon OR cameroons OR cameron OR camerons OR “Cape Verde” OR “Central African Republic” OR chad OR china OR colombia OR comoros OR “Comoro Islands” OR comores OR mayotte OR congo OR zaire OR “Costa Rica*“ OR “Cote d’Ivoire” OR “Ivory Coast” OR cuba OR djibouti OR “French Somaliland” OR dominica OR “Dominican Republic” OR “East Timor” OR “East Timur” OR “Timor Leste” OR ecuador OR egypt OR “United Arab Republic” OR “El Salvador” OR eritrea OR ethiopia OR fiji OR gabon OR “Gabonese Republic” OR gambia OR gaza OR “Georgia Republic” OR “Georgian Republic” OR ghana OR grenada OR guatemala OR guinea OR guiana OR guyana OR haiti OR honduras OR india OR maldives OR indonesia OR iran OR iraq OR jamaica OR jordan OR kazakhstan OR kazakh OR kenya OR kiribati OR korea OR kosovo OR kyrgyzstan OR kirghizia OR “Kyrgyz Republic” OR kirghiz OR kirgizstan OR “Lao PDR” OR laos OR lebanon OR lesotho OR basutoland OR liberia OR libya OR macedonia OR madagascar OR “Malagasy Republic” OR malaysia OR malaya OR malay OR sabah OR sarawak OR malawi OR mali OR “Marshall Islands” OR mauritania OR mauritius OR “Agalega Islands” OR mexico OR micronesia OR “Middle East” OR moldova OR moldovia OR moldovian OR mongolia OR montenegro OR morocco OR ifni OR mozambique OR myanmar OR myanma OR burma OR namibia OR nepal OR “Netherlands Antilles” OR “New Caledonia” OR nicaragua OR niger OR nigeria OR pakistan OR palau OR palestine OR panama OR paraguay OR peru OR philippines OR philipines OR phillipines OR phillippines OR “Puerto Ric*“ OR romania OR rumania OR roumania OR rwanda OR ruanda OR “Saint Lucia” OR “St Lucia” OR “Saint Vincent” OR “St Vincent” OR grenadines OR samoa OR “Samoan Islands” OR “Navigator Island” OR “Navigator Islands” OR “Sao Tome” OR senegal OR serbia OR montenegro OR seychelles OR “Sierra Leone” OR “Sri Lanka” OR “Solomon Islands” OR somalia OR “South Africa” OR sudan OR suriname OR surinam OR swaziland OR syria OR tajikistan OR tadzhikistan OR tadjikistan OR tadzhik OR tanzania OR thailand OR togo OR togolese AND republic OR tonga OR tunisia OR turkey OR turkmenistan OR turkmen OR uganda OR ukraine OR uzbekistan OR uzbek OR vanuatu OR “New Hebrides” OR venezuela OR vietnam OR “Viet Nam” OR “West Bank” OR yemen OR yugoslavia OR zambia OR zimbabwe OR “Developing Countries” OR africa OR asia OR caribbean OR “West Indies” OR “South America” OR “Latin America” OR “Central America” OR ( ( developing OR “less* developed” OR “under developed” OR underdeveloped OR “middle income” OR “low* income” OR underserved OR “under served” OR deprived OR poor*) W/1 ( countr* OR nation* OR population* OR world)) OR ( ( developing OR “less* developed” OR “under developed” OR underdeveloped OR “middle income” OR “low* income”) W/1 ( economy OR economies)) OR ( low* W/1 ( gdp OR gnp OR “gross domestic” OR “gross national”)) OR ( low W/3 middle W/3 countr*) OR lmic OR lmics OR “third world” OR “lami countr*“ OR “transitional countr*“)) AND ( ( TITLE-ABS-KEY ( ( ( systematic* OR synthes*) W/3 ( research OR evaluation* OR finding* OR thematic* OR report OR descriptive OR explanatory OR narrative OR meta* OR review* OR data OR literature OR studies OR evidence OR map OR quantitative OR study OR studies OR paper OR impact OR impacts OR effect* OR compar*)))) OR ( TITLE-ABS-KEY ( ( “meta regression” OR “meta synth*“ OR “meta-synth*“ OR “meta analy*“ OR “metaanaly*“ OR “meta-analy*“ OR “metanaly*“ OR “metaregression” OR “metaregression” OR “methodologic* overview” OR “pool* analys*“ OR “pool* data” OR “quantitative* overview” OR “research integration”))) OR ( TITLE-ABS-KEY ( ( review W/3 ( effectiveness OR effects OR systemat* OR synth* OR integrat* OR map* OR methodologic* OR quantitative OR evidence OR literature)))) OR ( TITLE-ABS-KEY ( ( “meta ethnograph*“ OR “meta synthesis” OR ( synthesis AND ( “qualitative literature” OR “qualitative research”)) OR “critical interpretive synthesis” OR ( “systematic review” AND ( “qualitative research” OR “qualitative literature” OR “qualitative stud*“)) OR “thematic synthesis” OR “framework synthesis” OR “realist review” OR “realist synthesis” OR “qualitative systematic review*“ OR “qualitative evidence synthes*“ OR ( ( “quality assessment” OR “critical appraisal” OR “literature search*“) AND ( “qualitative research” OR “qualitative literature” OR “qualitative stud*“)) OR ( noblit AND hare) OR “meta narrative*“ OR “narrative synthesis”))) OR ( TITLE-ABS-KEY ( ( random$ OR placebo$ OR “single blind$“ OR “double blind$“ OR “triple blind$“ OR cohort$ OR ( ( case$ OR cohort OR “follow up” OR follow-up) W/2 ( control$ OR series OR report$ OR study OR studies)) OR retrospective$ OR ( observ$ W/3 ( study OR studies)))))) AND ( LIMIT-TO ( PUBYEAR, 2020) OR LIMIT-TO ( PUBYEAR, 2019) OR LIMIT-TO ( PUBYEAR, 2018)) - **339**

1. **Web of Science (Social Sciences Citation Index) – Searched 26^th^ February 2020**

# 30 1,524

#29 AND #23 AND #15

Indexes=SSCI Timespan=2018-2020

# 29 129,728

#28 OR #27 OR #26 OR #25 OR #24

# 28 82,678

TS=(random$ or placebo$ or “single blind$“ or “double blind$“ or “triple blind$“ or cohort$ or ((case$ or cohort or “follow up” or follow-up) NEAR/2 (control$ or series or report$ or study or studies)) or retrospective$ or (observ$ NEAR/3 (study or studies)))

# 27 2,265

TS=(“meta ethnograph*“ or “meta synthesis” or (synthesis and (“qualitative literature” or “qualitative research”)) or “critical interpretive synthesis” or (“systematic review” and (“qualitative research” or “qualitative literature” or “qualitative stud*“)) or “thematic synthesis” or “framework synthesis” or “realist review” or “realist synthesis” or “qualitative systematic review*“ or “qualitative evidence synthes*“ or ((“quality assessment” or “critical appraisal” or “literature search*“) and (“qualitative research” or “qualitative literature” or “qualitative stud*“)) or (Noblit and Hare) or “meta narrative*“ or “narrative synthesis”)

# 26 32,626

TS=(review NEAR/3 (effectiveness or effects or systemat* or synth* or integrat* or map* or methodologic* or quantitative or evidence or literature))

# 25 25,301

TS=(“meta regression” or “meta synth*“ or “meta-synth*“ or “meta analy*“ or “metaanaly*“ or “meta-analy*“ or “metanaly*“ or “metaregression” or “metaregression” or “methodologic* overview” or “pool* analys*“ or “pool* data” or “quantitative* overview” or “research integration”)

# 24 26,382

TS=((systematic* or synthes*) NEAR/3 (research or evaluation* or finding* or thematic* or report or descriptive or explanatory or narrative or meta* or review* or data or literature or studies or evidence or map or quantitative or study or studies or paper or impact or impacts or effect* or compar*))

# 23 218,259

#22 OR #21 OR #20 OR #19 OR #18 OR #17 OR #16

# 22 1,125

TS=(lmic or lmics or “third world” or “lami countr*“ or “transitional countr*“)

# 21 2,953

TS=(low NEAR/3 middle NEAR/3 countr*)

# 20 286

TS=(low* NEAR (gdp or gnp or “gross domestic” or “gross national”))

# 19 2,100

TS=((developing or “less* developed” or “under developed” or underdeveloped or “middle income” or “low* income”) NEAR (economy or economies))

# 18 15,712

TS=((developing or “less* developed” or “under developed” or underdeveloped or “middle income” or “low* income” or underserved or “under served” or deprived or poor*) NEAR/1 (countr* or nation? or population? or world or state*))

# 17 207,412

TS=((Afghanistan or Albania or Algeria or Angola or Argentina or Armenia or Armenian or Aruba or Azerbaijan or Bahrain or Bangladesh or Benin or Byelarus or Byelorussian or Belarus or Belorussian or Belorussia or Belize or Bhutan or Bolivia or Bosnia or Herzegovina or Hercegovina or Botswana or Brasil or Brazil or Bulgaria or “Burkina Faso” or “Burkina Fasso” or “Upper Volta” or Burundi or Urundi or Cambodia or “Khmer Republic” or Kampuchea or Cameroon or Cameroons or Cameron or Camerons or “Cape Verde” or “Central African Republic” or Chad or China or Colombia or Comoros or “Comoro Islands” or Comores or Mayotte or Congo or Zaire or “Costa Rica*“ or “Cote d’Ivoire” or “Ivory Coast” or Cuba or Djibouti or “French Somaliland” or Dominica or “Dominican Republic” or “East Timor” or “East Timur” or “Timor Leste” or Ecuador or Egypt or “United Arab Republic” or “El Salvador” or Eritrea or Ethiopia or Fiji or Gabon or “Gabonese Republic” or Gambia or Gaza or “Georgia Republic” or “Georgian Republic” or Ghana or Grenada or Guatemala or Guinea or Guiana or Guyana or Haiti or Honduras or Hungary or India or Maldives or Indonesia or Iran or Iraq or Jamaica or Jordan or Kazakhstan or Kazakh or Kenya or Kiribati or Korea or Kosovo or Kyrgyzstan or Kirghizia or “Kyrgyz Republic” or Kirghiz or Kirgizstan or “Lao PDR” or Laos or Lebanon or Lesotho or Basutoland or Liberia or Libya or Macedonia or Madagascar or “Malagasy Republic” or Malaysia or Malaya or Malay or Sabah or Sarawak or Malawi or Mali or “Marshall Islands” or Mauritania or Mauritius or “Agalega Islands” or Mexico or Micronesia or “Middle East” or Moldova or Moldovia or Moldovian or Mongolia or Montenegro or Morocco or Ifni or Mozambique or Myanmar or Myanma or Burma or Namibia or Nepal or “Netherlands Antilles” or “New Caledonia” or Nicaragua or Niger or Nigeria or Muscat or Pakistan or Palau or Palestine or Panama or Paraguay or Peru or Philippines or Philipines or Phillipines or Phillippines or “Puerto Ric*“ or Romania or Rumania or Roumania or Rwanda or Ruanda or “Saint Lucia” or “St Lucia” or “Saint Vincent” or “St Vincent” or Grenadines or Samoa or “Samoan Islands” or “Navigator Island” or “Navigator Islands” or “Sao Tome” or Senegal or Serbia or Montenegro or Seychelles or “Sierra Leone” or “Sri Lanka” or “Solomon Islands” or Somalia or “South Africa” or Sudan or Suriname or Surinam or Swaziland or Syria or Tajikistan or Tadzhikistan or Tadjikistan or Tadzhik or Tanzania or Thailand or Togo or Togolese Republic or Tonga or Tunisia or Turkey or Turkmenistan or Turkmen or Uganda or Ukraine or Uzbekistan or Uzbek or Vanuatu or “New Hebrides” or Venezuela or Vietnam or “Viet Nam” or “West Bank” or Yemen or Yugoslavia or Zambia or Zimbabwe) NOT (“African-American*“ OR “African-American*“ OR “Mexican American*“ OR “American Indian*“ OR “Asian American*“ OR “native american*“)) OR CU=((Afghanistan or Albania or Algeria or Angola or Argentina or Armenia or Armenian or Aruba or Azerbaijan or Bahrain or Bangladesh or Benin or Byelarus or Byelorussian or Belarus or Belorussian or Belorussia or Belize or Bhutan or Bolivia or Bosnia or Herzegovina or Hercegovina or Botswana or Brasil or Brazil or Bulgaria or “Burkina Faso” or “Burkina Fasso” or “Upper Volta” or Burundi or Urundi or Cambodia or “Khmer Republic” or Kampuchea or Cameroon or Cameroons or Cameron or Camerons or “Cape Verde” or “Central African Republic” or Chad or China or Colombia or Comoros or “Comoro Islands” or Comores or Mayotte or Congo or Zaire or “Costa Rica*“ or “Cote d’Ivoire” or “Ivory Coast” or Cuba or Djibouti or “French Somaliland” or Dominica or “Dominican Republic” or “East Timor” or “East Timur” or “Timor Leste” or Ecuador or Egypt or “United Arab Republic” or “El Salvador” or Eritrea or Ethiopia or Fiji or Gabon or “Gabonese Republic” or Gambia or Gaza or “Georgia Republic” or “Georgian Republic” or Ghana or Grenada or Guatemala or Guinea or Guiana or Guyana or Haiti or Honduras or Hungary or India or Maldives or Indonesia or Iran or Iraq or Jamaica or Jordan or Kazakhstan or Kazakh or Kenya or Kiribati or Korea or Kosovo or Kyrgyzstan or Kirghizia or “Kyrgyz Republic” or Kirghiz or Kirgizstan or “Lao PDR” or Laos or Lebanon or Lesotho or Basutoland or Liberia or Libya or Macedonia or Madagascar or “Malagasy Republic” or Malaysia or Malaya or Malay or Sabah or Sarawak or Malawi or Mali or “Marshall Islands” or Mauritania or Mauritius or “Agalega Islands” or Mexico or Micronesia or “Middle East” or Moldova or Moldovia or Moldovian or Mongolia or Montenegro or Morocco or Ifni or Mozambique or Myanmar or Myanma or Burma or Namibia or Nepal or “Netherlands Antilles” or “New Caledonia” or Nicaragua or Niger or Nigeria or Muscat or Pakistan or Palau or Palestine or Panama or Paraguay or Peru or Philippines or Philipines or Phillipines or Phillippines or “Puerto Ric*“ or Romania or Rumania or Roumania or Rwanda or Ruanda or “Saint Lucia” or “St Lucia” or “Saint Vincent” or “St Vincent” or Grenadines or Samoa or “Samoan Islands” or “Navigator Island” or “Navigator Islands” or “Sao Tome” or Senegal or Serbia or Montenegro or Seychelles or “Sierra Leone” or “Sri Lanka” or “Solomon Islands” or Somalia or “South Africa” or Sudan or Suriname or Surinam or Swaziland or Syria or Tajikistan or Tadzhikistan or Tadjikistan or Tadzhik or Tanzania or Thailand or Togo or Togolese Republic or Tonga or Tunisia or Turkey or Turkmenistan or Turkmen or Uganda or Ukraine or Uzbekistan or Uzbek or Vanuatu or “New Hebrides” or Venezuela or Vietnam or “Viet Nam” or “West Bank” or Yemen or Yugoslavia or Zambia or Zimbabwe) NOT (“African-American*“ OR “African-American*“ OR “Mexican American*“ OR “American Indian*“ OR “Asian American*“ OR “native american*“))

# 16 31,104

TS=(Africa or Asia or Caribbean or “West Indies” or “Middle East” or “South America” or “Latin America” or “Central America”) or CU=(Africa or Asia or Caribbean or “West Indies” or “Middle East” or “South America” or “Latin America” or “Central America”)

# 15 31,763

#14 OR #13 OR #12 OR #11 OR #10 OR #9 OR #8 OR #7 OR #6 OR #5 OR #4 OR #3 OR #2 OR #1

# 14 408

TI=(physical* NEAR/5 (impair* or deficien* or disable* or disabili* or handicap*))

# 13 110

TI=((“cerebral pals*“ or “spina bifida” or “muscular dystroph*“ or arthriti* or “osteogenesis imperfecta” or “musculoskeletal abnormalit*“ or “musculo-skeletal abnormalit*“ or “muscular abnormalit*“ or “skeletal abnormalit*“ or “limb abnormalit*“ or “brain injur*“ or amput* or clubfoot or polio* or paraplegi* or paralys* or paralyz* or hemiplegi* or stroke* or “cerebrovascular accident*“) NEAR/2 (impair* or disabilit* or disabl* or handicap*))

# 12 1,845

TI=(((visual* or vision or eye* or ocular) NEAR/5 (loss* or impair* or deficien* or disable* or disabili* or handicap*)) or blind*)

# 11 1,194

TI=(((hearing or acoustic or ear*) NEAR/5 (loss* or impair* or deficien* or disable* or disabili* or handicap*)) or deaf*)

# 10 4,909

TI=((intellectual* or educational* or mental* or psychological* or developmental) NEAR/5 (impair* or retard* or deficien* or disable* or disabili* or handicap* or ill*))

# 9 7,175

TI=(autis* or dyslexi* or “Down* syndrome” or mongolism or “trisomy 21”)

# 8 4,557

TI=((mental* or emotional* or psychiatric or neurologic*) NEAR/2 (disorder* or ill or illness*))

# 7 378

TI=((schizophreni* or psychos* or psychotic or schizoaffective or schizophreniform or dementia* or alzheimer*) NEAR/2 (impair* or disabilit* or disabl* or handicap*))

# 6 9,358

TI=(“mental health”)

# 5 1,830

TS=((depression or depressive or anxiety or psychiat* or well-being or “quality of life” or self-esteem or “self perception”) NEAR/2 (impair* or disabilit* or disabl* or handicap*))

# 4 506

TI=((communication or language or speech or learning) NEAR/5 disorder*)

# 3 5,906

TI=((cognitive* or learning or mobility or sensory or visual* or vision or sight or hearing or physical* or mental* or intellectual*) NEAR/2 (impair* or disabilit* or disabl* or handicap*))

# 2 3,336

TI=((physical* or intellectual* or learning or psychiatric* or sensory or motor or neuromotor or cognitive or mental* or developmental or communication or learning) NEAR/2 (disabilit* or disabl* or handicap*))

# 1 2,568

TI=((disable* or disabilit* or handicapped) NEAR/5 (person* or people or child* or adolescen* or women or mother* or maternal or group*))

1. **WHO Global Health Index (2016-2020) – Searched 26^th^ February 2020**

**Total after duplicates removed: 1054**

Search 1: tw: (((disable* OR disabilit* OR handicapped OR “mental health” OR impair*) AND ((systematic* OR synthes*) AND (research OR evaluation* OR finding* OR thematic* OR report OR descriptive OR explanatory OR narrative OR meta* OR review* OR data OR literature OR studies OR evidence OR map OR quantitative OR study OR studies OR paper OR impact OR impacts OR effect* OR compar*)))) AND (instance:“ghl”) AND ( db:(“LILACS” OR “WPRIM” OR “WHOLIS” OR “IMEMR” OR “AIM”) AND mj:(“Mental Health” OR “Disabled Persons” OR “Hearing Loss” OR “Quality of Life” OR “Mental Disorders” OR “Intellectual Disability” OR “Disability Evaluation” OR “Mental Health Services”) AND year_cluster:(“2020” OR “2019” OR “2018” OR “2017” OR “2016”))

Search 2: tw:((disable* OR disabilit* OR handicapped OR “mental health” OR impair*) AND (review AND (effectiveness OR effects OR systemat* OR synth* OR integrat* OR map* OR methodologic* OR quantitative OR evidence OR literature))) AND (instance:“ghl”) AND ( db:(“LILACS” OR “WPRIM” OR “WHOLIS” OR “IMEMR” OR “AIM”) AND mj:(“Mental Health” OR “Disabled Persons” OR “Hearing Loss” OR “Quality of Life” OR “Mental Disorders” OR “Intellectual Disability” OR “Disability Evaluation” OR “Mental Health Services”) AND year_cluster:(“2020” OR “2019” OR “2018” OR “2017” OR “2016”))

Search 3: tw:((disable* OR disabilit* OR handicapped OR “mental health” OR impair*) AND (random* OR placebo* OR “single blind*“ OR “double blind*“ OR “triple blind*“ OR cohort*)) AND (instance:“ghl”) AND ( db:(“LILACS” OR “WPRIM” OR “WHOLIS” OR “IMEMR” OR “AIM”) AND mj:(“Mental Health” OR “Disabled Persons” OR “Hearing Loss” OR “Quality of Life” OR “Mental Disorders” OR “Intellectual Disability” OR “Disability Evaluation” OR “Mental Health Services”) AND year_cluster:(“2020” OR “2019” OR “2018” OR “2017” OR “2016”))

Search 4: tw:(((disable* OR disabilit* OR handicapped OR “mental health” OR impair*) AND (“meta regression” OR “meta synth*“ OR meta-synth* OR “meta analy*“ OR metaanaly* OR meta-analy* OR metanaly*))) AND (instance:“ghl”) AND ( db:(“LILACS” OR “WPRIM” OR “WHOLIS” OR “IMEMR” OR “AIM”) AND mj:(“Mental Health” OR “Disabled Persons” OR “Hearing Loss” OR “Quality of Life” OR “Mental Disorders” OR “Intellectual Disability” OR “Disability Evaluation” OR “Mental Health Services”) AND year_cluster:(“2020” OR “2019” OR “2018” OR “2017” OR “2016”))

Search 5: tw:((disable* OR disabilit* OR handicapped OR “mental health” OR impair*) AND (((case* OR cohort OR “follow up” OR follow-up) AND (control* OR series OR report* OR study OR studies)) OR retrospective* OR (observ* AND (study OR studies)))) AND (instance:“ghl”) AND ( db:(“LILACS” OR “WPRIM” OR “WHOLIS” OR “IMEMR” OR “AIM”) AND mj:(“Mental Health” OR “Disabled Persons” OR “Hearing Loss” OR “Quality of Life” OR “Mental Disorders” OR “Intellectual Disability” OR “Disability Evaluation” OR “Mental Health Services”) AND year_cluster:(“2020” OR “2019” OR “2018” OR “2017” OR “2016”))

## 2 Coding Tool

- Publication status
  - Published
  - Ongoing
- Region
  - East Asia and Pacific
  - Europe and Central Asia
  - Latin America and Caribbean
  - Middle East and North Africa
  - Sub-Saharan Africa
  - South Asia
- Country (specify)
  - Iran
  - India
  - Albania
  - South Africa
  - Turkey
  - Uganda
  - Indonesia
  - Egypt
  - China
- Type of disability
  - Hearing
  - Physical
  - Visual
  - Intellectual/learning and developmental/behavioural
  - Psychosocial/Mental
  - Can’t tell/not reported
- Target group
  - People with disability_Child*Child (0-17.9 years)*
  - People with disability_adults
  - People with disability_elderly
  - Family member/caregiver
  - Service provider/professional/teachers
  - Community member
  - Other (specify)
- Participants SES
  - Low
  - Medium
  - High
  - Mixed
  - Can’t tell/not reported
- Gender of target group
  - Male
  - Female
  - Both
  - Can’t tell/not reported
- Study design
  - Randomised controlled trial
  - Controlled before and after
  - RDD
  - ITS
  - Matched designs
  - Others
- Subject assignment
  - Individual random
  - Whole group random
  - Individual matched random
  - Non-matched and non-random
  - Other (specify)
  - Can’t tell/not reported
- Geographical setting of the interventions
  - Urban
  - Rural
  - Mixed
  - Can’t tell/not reported
- Interventions
  - Personal assistance
    - Formal personal assistance and support (including training)*Formal assistance may be provided on a formal basis by governmental and nongovernmental organizations and the private sector. Allowances, such as disability pensions, guardianship awards or caregiver allowances, may be available to fund personal assistance. (Khasnabis, Al Jubah, Brodtkorb, Chervin, P Goerdt,*
    - Informal personal assistance and support (including training)*Informal assistance includes assistance by family members, friends, neighbours and/or volunteers (Khasnabis, Al Jubah, Brodtkorb, Chervin, P Goerdt,)*
  - Relationship, marriage and family
    - Networking and social support*Includes linking people with disabilities to appropriate support networks in the community, e.g. disabled people’s organizations and self-help groups. (Khasnabis, Al Jubah, Brodtkorb, Chervin, P Goerdt,*
    - Improving community attitude*It involve working with the media to promote positive images and role models of people with disabilities; and information on services available (Khasnabis, Al Jubah, Brodtkorb, Chervin, P Goerdt,*
    - Community living*It involves interventions to support people with disabilities to access their preferred living arrangements and support people with disabilities who are homeless to find appropriate accommodation, preferably in the community*
    - Social and communication skill training*Social skills training is a therapeutic approach used to improve interpersonal relations. The therapy focuses on verbal and nonverbal behaviors common in social relationships.*
    - Violence prevention interventions*This includes all the interventions to prevent violence such as raising awareness, establishing links to local stakeholders for support, access to health care services etc. (Khasnabis, Al Jubah, Brodtkorb, Chervin, P Goerdt,*
  - Culture and arts
    - Access and participation in cultural programs, arts, drama and theatres*People with disabilities enjoy access and participation to cultural materials in accessibleformats; to television programmes, films, theatre and other cultural activities, in accessibleformats; to places for cultural performances or services, such as theatres, museums, cinemas, libraries and tourism services, and, as far as possible, to monuments and sites of national cultural importance. (Article 30 of the UN CRPD)*
    - Access and participation in religious activities*People with disabilities enjoy access and participation in religious and spiritual activities in accessible formats, e.g. making prayers, songs, chanting, and sermons accessible with signed translation, and making religious texts available in large print, audio and Braille; Places of worship are physically accessible and that religious practices are modified to accommodate people with disabilities. (Article 30 of the UN CRPD)*
  - Recreation, leisure and sports
    - Access and participation in sports events*This includes strategies that encourages people with disabilities to have access and provide opportunities to participate in mainstream sporting activities at all levels through inclusive sports event; have an opportunity to organize, develop and participate in disability-specific sporting and recreational activities through provision of support and links with DPOs for people with disabilities, assisting them to develop strategic, national and international partnerships and have access to adapted sports equipment. (Khasnabis, Al Jubah, Brodtkorb, Chervin, P Goerdt,)*
    - Access and participation in recreation and leisure*This includes strategies that encourages people with disabilities to have access and provide opportunities to participate in mainstream sporting activities to provide opportunities to participate actively or passively in recreation, tourism and leisure. (Khasnabis, Al Jubah, Brodtkorb, Chervin, P Goerdt,*
  - Access to justice
    - Accessibility of legal system and justice*Accessibility”, in this publication refers to a feature or quality of any physical or virtual environment, space, facility or service that is capable of accommodating the needs of people with disabilities to understand, get access to or interact with legal system. Accessibility also refers to technical standards that are mandated nationally or internationally for the design and construction of a physical or virtual environment, space, facility and service. Examples include accessible built infrastructure of courts such as ramps etc.*
    - Access to legal system and justice*Refers to people’s ability to access the systems, procedures, information, and locations used in the administration of justice (Lord JE, 2008) This includes activities such as legal awareness through DPOs and media, legal aid.*
  - AT and rehabilitation
    - Assistive technology
    - Rehabilitation*Rehabilitation is a process intended to eliminate or at least minimize – restrictions on the activities of people with disabilities, permitting them to become more independent and enjoy the highest possible quality of life (Bailey R, 2005). This will include activities as provision of mobility, hearing, visual devices, and therapies to use these devices.*
    - Medical care*Provision of medical services to ensure that people with disabilities can access services designed to identify, prevent, minimize and/or correct health conditions and impairments. (Khasnabis, Al Jubah, Brodtkorb, Chervin, P Goerdt,)*
  - Policies and programmes
    - International legislations and policies*These include international legislations and policies through which countries abolish discrimination against persons with disabilities and eliminate barriers towards the full enjoyment of their rights and their inclusion in society. (UN Department of Economic and Social Affairs*
    - Social inclusion policies*This includes inclusive policies on employment, educational and provision of housing and accommodation to people with disabilities*
- Outcomes
  - Social
    - Social identity*Social identity is defined asthat part of a person’s self-concept which derives fromthe knowledge of his or hermembership in a social group(or groups) together with the value and emotional significance attached to that membership.Social identity can spur intergroup discrimination and other formsof intergroup conflict (Simon & Trötschel)*
    - Personal assistance*People with disabilities have individual support plans in place, have access to training to enable them to manage their personal assistance needs, or that support is available for families who provide personal assistance on an informal basis.*
  - Skills for social inclusion
    - Social and communication skills*Kratchowill and French* *(1984) view social skills as learned verbal and non-verbal behaviour performed within a specific social context of an aggressiveness-shyness continuum, and view adjustment in relation to an individual’s social perceptual accuracy (thatis, the ability to understand subtle nuances and define critical elements in social environment). Examples include civic, social engagement and interaction and professional social skills.*
    - Communication skills*It is the act of transferring information. It may be vocally (using voice), written (using printed or digital media such as books, magazines, websites or emails), visually (using logos, maps, charts or graphs) or non-verbally (using body language, gestures and the tone and pitch of voice). This includes availability and use of communication aids and speech and reading devices*
    - Social behavior*Social behavior can be defined as all behavior that influences, or is influenced by, other members of the same species. The term thus covers all behavior that tends to bring individuals together as well as all forms of aggressive behavior (Grant, 1963). This includes conduct problems, peer problems, pro-social behaviours*
  - Broad based social inclusion and participation measure
    - Social inclusion*Social inclusion is defined as the process of improving the terms of participation in society, particularly for people who are disadvantaged, through enhancing opportunities, access to resources, voice and respect for rights. (UN 2010). These will include measures such as people with disabilities spending more time out of the house, and travelling further away from the house (as well as earning more and spending* *less time begging)*
    - Community integration*Community Integration is the opportunity to live in the community and be valued for one’s uniqueness and abilities, like everyone else. (Salzer, 2006). Community integration is designed to help people with disabilities to optimize their personal, social, and vocational competency to live successfully in the community.*
    - Community participation*People with disabilities have access, accessibility and opportunities to participate in community activities such as leisure activities, such as hobbies, arts, and sports, political and civic activities or organizations and productive activities, like* *employment or education; consumption, or access to goods and services; religiousand cultural activities and groups (McConkey, 2007; Verdonschot et al., 2009)..*
    - Access to justice*People with disabilities get access to or interact with legal system*
  - Relationships
    - Interpersonal and Family relationship*People with disabilities valuerelationships with family members, staff, friends, acquaintances, and intimate partners (Clarkson, Murphy, Coldwell, & Dawson, 2009) and other peoplewith disabilities (McVilly et al., 2006b), and feeling a sense of belonging to a network when they have different people fulfilling different needs (McVilly et al., 2006a).This also includes aspects of participation in household, behaviour of the family towards the people with disability (e.g. more sensitive to child’s interests, responded moreappropriately, expressed more warmth)*
    - Peer and community relationships*Community members are aware and accept that people with disabilities can have meaningful relationships, marry and have children. (Community-Based Rehabilitation: CBR Guidelines)*
    - Violence and abuse*People with disabilities are protected against violence, and all relevant stakeholders work together to address the issue. (Community-Based Rehabilitation: CBR Guidelines)*
- Duration of study (specify)
- Duration of interventions (specify?)
- Upon what kind of statistical analysis were the major findings of the study based?
  - Descriptive analysis
  - t-test
  - ANOVA
  - ANCOVA
  - Regression
  - Other (specify)
- Intervention was delivered by?
  - Intervention therapist/coach/occupational therapist
  - Community members
- Sample size of intervention group
- Sample size of control group
- Outcomes details
  - Are descriptive statistics reported for the primary outcome?
    - Yes
      - If yes, please add for the intervention* group*Descriptive statistics for the intervention group. *If there is more than one intervention group please add this below.*
        - Number (n)*What is the number for the intervention group in the data analysed for this outcome? Add numeric data only to the info box.*
        - Pre-test mean*Please record the pre-test mean (if provided) for the intervention group for this outcome. Add numeric data only to the info box.*
        - Pre-test standard deviation*Please record the pre-test standard deviation (if provided) for the intervention group for this outcome. Add numeric data only to the info box.*
        - Post-test mean*Please report the post-test mean for this outcome for the intervention group (if provided) for this outcome. Add numeric data only to the info box.*
        - Post test standard deviation*Please record the post-test standard deviation for the intervention group for this outcome (if provided). Add numeric data only to the info box.*
        - Gain score mean (if reported)*Please add the gain score (pre-test to post test) mean for the intervention group. Add numeric data only to the info box.*
        - Gain score standard deviation (if reported)*Please add the gain score (pre-test to post test) standard deviation for the intervention group. Add numeric data only to the info box.*
        - Any other information?*Please add any other statistical information reported about this outcome for the intervention group (e.g. standard error (SE)), or use to add notes about the numeric data in the categories above.*
      - If yes please add for the control group*Descriptive statistics for the intervention group*
        - Number (n)*What is the number for the control group in the data analysed for this outcome? Add numeric data only to the info box.*
        - Pre-test mean*Please record the pre-test mean (if provided) for the control group for this outcome. Add numeric data only to the info box.*
        - Pre-test standard deviation*Please record the pre-test standard deviation (if provided) for the control group for this outcome. Add numeric data only to the info box.*
        - Post-test mean*Please report the post-test mean for this outcome for the control group (if provided) for this outcome.*
        - Post test standard deviation*Please record the post-test standard deviation for the control group for this outcome (if provided).*
        - Gain score mean (if reported)*Add numeric data only to the info box.*
        - Gain score standard deviation (if reported)*Add numeric data only to the info box.*
        - Any other information?*Please add any other statistical information reported about this outcome for the intervention group (e.g. standard error (SE)).*
    - No
  - Is there follow up data?*Please provide details of any assessment to measure long lasting effects (e.g. delayed post-test or long term follow up)*
    - Yes
    - No
- Critical appraisal
  - Study design (Potential confounders taken into account)
    - LOW: Before versus after. Naïve matching
    - MEDIUM: IV, RDD, PSM, double difference
    - HIGH: RCT, natural experiment
  - Blinding (RCTs only)
    - LOW: No mention of blinding
    - MEDIUM: Blinding for analysis
    - HIGH: Blinding of data collection (where feasible) and blinding for analysis
  - Losses to follow up are presented and acceptable
    - LOW: Attrition not reported, OR falls well outside WWC acceptable combined levels*
    - MEDIUM: Overall and differential attrition close to WWC combined levels*
    - HIGH: Overall and differential attrition within WWC combined levels*
  - Disability/impairment measure is clearly defined and reliable
    - LOW: No definition OR overall attrition > 50%
    - MEDIUM: Unclear definition OR Single question item only (e.g. are you disabled)
    - HIGH: Clear definition, e.g. Washington Group questions, detailed measure of impairment
  - Outcome measures are clearly defined and reliable
    - LOW: No definition
    - MEDIUM: Unclear definition
    - HIGH: Clear definition using existing measure where possible
  - Baseline balance (N.A. for before versus after)
    - LOW: No baseline balance test (except RCT) OR reported and significant differences on more than five measures. PSM without establishing common support.
    - MEDIUM: Baseline balance test, imbalance on 5 or fewer measures
    - HIGH: RCT, RDD
  - Overall confidence in study findings
    - LOW: Low on any item
    - MEDIUM: Medium or high confidence on all items
    - HIGH: RCT with high confidence on all items

## 3 Characterstics of included studies

| Study | Country/context | Design | Participants and sample size | Intervention | Social inclusion outcomes | Results |
| --- | --- | --- | --- | --- | --- | --- |
| Abazari et al., 2017 | Iran | Controlled before and after | Participant were children under the age of 7 years with Autism Spectrum Disorder. Sample size: 20, 10 in experimental and 10 in control group. | Social and communication skills training: The expressed emotion-based Floortime intervention program was presented in 23 sessions of 2 hours each, once aweek | Interpersonal and family relationship. Demographic characteristics questionnaire, ASSQ test,Stanford Binet intelligence test, Family Questionnaire (FQ),Quiz Questions Expressed Emotion, Gilliam autism scale,Social skills test (villand) were used as measuring tools | Asignificant difference was revealed between parents of allthree sub-scales, namely total score of expressed emotion(P=0.0001), extreme emotional involvement (P=0.0001),and critique (P=0.003). |
| Amaresha et al., 2018 | India | Randomised controlled trial | Siblings of persons with schizophrenia. Sample size: 80, 40 in experimental group of brief need based psychoeducation group and 40 in control group of treatment-as-usual. | Brief psychoeducation therapy | Social identify and self-stigma | There was a significant increase in knowledge and reduction in self-stigma with medium effect size through baseline to the third month follow-up as compared to the treatment as usual group. |
| Azari et al., 2019 | Iran, Two rehabilitation centers (Navid-e-asr and Omid-e-asr rehabilitation centers) in Tehran | Randomized Controlled Trial with a mixed within between-subjects design and | Participants were parents of children aged 3-10 with Autism Spectrum Disorder  Sample size: 38 families-19 were randomised into intervention group and 19 to control group | Informal personal assistance and support (including training). The intervention includes contextually reflective occupational therapy (CI-ASD) which contains three elements, namely - sensory processing patterns, coaching, and social support. The coach provided 2 training group sessions and 10 individual sessions of coaching (over 11 wk) for each mother to recognize strategies for improving their child’s participation to achieve functional goals. | Personal assistance: Greater gains in children’s participation and parenting efficacy, as measured through Canadian Occupational Performance Measure, Goal Attainment Scaling, and Parenting Sense of Efficacy Measure | The participants in the intervention group indicate greater gains in children’s participation and parenting efficacy, relative to the control group. Repeated measures ANOVA showed that the time, group and time x group interaction had significant effects on COPM performance (P ˂ .001), COPM satisfaction (P ˂ .001), GAS scores (P ˂ .001) and PSEM scores (P ˂ .013). Based on the t-test conducted, the study reveals that both control and treatment groups show an improvement in participation in children, with a statistically significant difference in favour of the treatment group. A significant difference in parenting efficacy was also found in the intervention group, compared to the wait-list group, as manifested in the Parenting Self-Efficacy measure. |
|  |  |  |  |  |  |  |
| Dai et al., 2018 | Alabania | Randomized Controlled trial | Children with disabilities (18-70 months) and parents as mediators of interventions Sample size: 29 Treatment (n=13) Control (n=16) | Informal personal assistance and support. Parent training programme-6 DVD modules with clips of ABA based interventions | Use of personal assistance and parent knowledge | Parents rated. The programme reported that children responded well and quiz score for parents in the treatmenet group increased by 8 points. |
| de Villiers et al., 2013 | South Africa, special schools | Controlled before and after | Chidren with physical disabilities, average age 15 years (13-17 years). Sample size: 24 participants (Treatment (n=12) Control (n=12) | Access and participation in recreation and leisure. Wheelchair dancing | Social identity. Feelings of Inadequacy Scale (JFS) was used, to assess multiple components of self-esteem5 | 72.7% of the intervention group displayed an increase in self-esteem, as compared to only 54.6% of the control group, however this was not statistically significant using the 95% CI. However, it was clinically evaluated by the occupational therapist via Byslma’s 7-point criteria that the intervention group had improved in their self-esteem and outlook. |
| Devries, et al., 2018 | Uganda, Luwero District Primary Schools | Cluster randomized controlled trial | 42 schools, 1,899 students(including 278 children with some functional difficulties, and 104 children with disabilities. Total number of clusters were 42 and participants per cluster were less than 130 | Violence prevention intervention. Good School Toolkit: a school-based multicomponent intervention to reduce violence against students by school staff involving behaviour change techniques that engage students, school staff and parents, including setting school-wide goals, developing action plans, reflection on experiences of violence to encourage empathy, and improving knowledge on positive forms of discipline. Schools work through six steps in the Toolkit manual. The programme is led by a small team of staff and student ‘protagonists' | Primary outcomes: past week physical violence by school staff (self-reported by students and school staff) | The trial showed that after the intervention the prevalence of physical violence perpetrated by school staff in the past week towards students with some functional difficulties and students with disabilities was lower in intervention schools than in the control schools. The intervention also reduced violence perpetrated by peers. |
| Esmaili et al., 2019) | Iran | Randomized controlled trial | Children with learning disability in the age of 7-11 years. Sample size: 49 children, treatment group=25 and control group =24. Percentage female (28% and 29%). | Social and communication skill training. Peer-play activities (9 weeks) Control: 24 children receiving no treatment in the intervention phase | Social and communication skills. ehavior Rating Inventory of Executive Function (BRIEF) - Behaviour Regulation Index (BRI) and Metacognition Index (MCI), and the Child Occupational Self Assessment (COSA) - Value Scale and Competence Scale | There was a significant improvement in BRIEF indices post-intervention, with mean scores improving by 8,52 for BRI, and MCI 15.32 as compared to the control group. Thus, the results show that peer-play activities can be used to enhance executive function in children with SLD. |
| Golzari et al., 2015 | Iran, Special Education Organization in Shiraz city | Randomized controlled trial | Children with autism spectrum disorder in the age 6-12 years. Sample size: 30 male students with ASD who were selected through convenience sampling and randomly assigned to an experimental group (n = 15) or a control group (n = 15). | Social stories intervention. experimental group participated in 16 sessions of social stories training. | Social skills assessed using the Social Skills Rating Form, a subscale of the Triad Social Skills Assessment (TSSA; second edition) | Social stories intervention improved the social skills of the children with ASD in the experimental group compared with the control group. |
| Govindraj et al., 2018 | India, Tertiary care neuropsychiatric hospital in South India | Single group pre-post | Patients with schizophrenia in the age group of 18-45 years. Sample size: 15 | Add-on yoga therapy,  participants  attended  twenty sessions of yoga over 6 weeks. Each session lasted  for 1 h. | Social congnition | There was a significant improvement in the social cognition composite score after 20 sessions of yoga (t = −5.37, P≤ 0.001). |
| Hanlon et al., 2020 | Ethiopia, Sododistrict, in theGurage Zone  ofthe Southern Nations | Intervention cohort design | Adults with severe mental illness. Sample size: 294, T1: 247 and T2: 245 | Programme for Improving  Mental health carE (PRIME) | Community discrimination, violence and abuse | Being restrained in the pre-  vious 12 months reduced from 25.3 to 10.6%, and discrimination scores reduced significantly |
| Juneja et al., 2012 | India, two child development centre in New Delhi | Controlled before and after | Children with ASD less than 6 years. Sample size: 36 | Social and communication skills training | Social and communication skills. | There was a significant improvement in the  development quotient, social quotient, expressive language quotient after the intervention. |
| Kalgotra, 2017 | India | Controlled before and after | 21 children with intellectual disability. Treatment group=11 and control group=10, males=15 and female=6. | Music therapy (Sing and play music.) 60 minutes during school hours, 5 days a week for 24 weeks | Social behaviour- Music therapy had a positive impact on the person with an intellectual disability, including a decrease in disruptive behaviours | Significant changes were observed in the domains of violent and destructive behaviour and misbehaviours with others domains of children with intellectual disability. |
| Karaman et al., 2020 | Turkey | Randomised controlled trial | Sample size: 64, treatment group-43 and control group-21 | Adjunct Psychosocial Skills Training (PSST), involves verbal and nonverbal components of social  behavior, role-playing method, corrective and supportive feedback. | Social behaviour and social and communication skills. | Intervention had a positive  effect on the social functioning. |
| Karanth et al., 2010 | India, Bangalore city | Randomised controlled trial | Children with autism spectrum disorder. Sample size: 30 children in the age range of 2.2 to 5.5 years | The Communication DEALL (Developmental Eclectic Approach to Language Learning). tensive stimulation (3 hours/day, 5 days/week, over an academic year) | Social and communication skills. | There was improvement in communication skills and behavioural issues in children. |
| Khalil, 2019 | Egypt, in hospital setting | Controlled before and after | 60 adults with psychosocial impairment. Treatment group=30 and control group=30. Age range 18-65 | Social and communication skill training. Behavioural family psychoeducational programme (BFPEP) | Skills for social inclusion. Statistically significant improvements were detected in social functioning and | Statistically significant improvements were detected in social functioning and quality of life of cases compared to controls |
| Koo & Thomas, 2019 | India | Controlled before and after | 18 Children with intellectual/developmental disability (ASD). Treatment group=9 and control group=9. Age range, 4-12 years. | Access and participation in cultural programs, arts, drama, and theatre. Art therapy sessions were given for 10 weeks. | Skills for social inclusion. | Art therapy was significantly effective in enhancing social and communication skills |
| Lal, 2007 | India | Uncontrolled before and after with naive matching | 30 children with intellectual/developmental disability (ASD) in the age range 5-11 years.. Treatment group=15 and control group=15. | Social and communication skills training. 14 one-to-one sessions focused on development of comprehension, labelling, description, joint attention, and active interaction through visual supports | Skills for social inclusion. | Visual strategies were found to be effective in the development of communication skills of children with ASD |
| Lee et al., 2019 | China | Uncontrolled before and after | 8 children with intellectual/developmental disability (ASD). Age range, 7-8 years, male 7, and female=1.. | Social and communication skill interventions. 10 group sessions and 4 | Skills for social inclusion | The results indicated that the children’s emotional skills, behavioral and emotional competence, and adaptive functioning in communication were significantly improved after the intervention. |
| Li et al., 2018 | China | Randomised controlled trial | 294 people with Schizophrenia. Treatment group=199 and control group=185 participants. Age range, 18-50 years. | Formal personal assistance and support; Improving community attitude and social and communication skills training. | Skills for social inclusion and broad-based social inclusion and participation measures. Participants were measured at baseline, 6 months, and 9 months | At 6 months and 9 months, mean scores of overcoming stigma in intervention group were significantly higher than the control group (both p < 0.001). |
|  |  |  |  |  |  |  |
| Li et al., 2019 | China, four district in Guangzhou city | Randomised controlled trial | 293 care assistant workers from four districts of Guangzhou  an intervention group (n = 139) and a control group (n = 154). | Anti-stigma training related to mental illness | Community attitude and peer and community relationship measured using Perceived Devaluation and Discrimination Scale (PDD), Mental illness: Clini‑  cians’ Attitudes (MICA) and Mental Health Knowledge Schedule (MAKS). | Anti‑stigma training was effective in reducing the perception of deval‑  uation‑discrimination against people with mental illness and decreasing the level of negative stigma‑related mental  health attitudes among care assistant workers. |
| Liang et al., 2020 | China | Randomised controlled trial | Children with ASD less than 48 months. 80 children, 40 in treatment and 40 in control group | Parent training intervention. Group received 12 weeks of  training, including 8 group sessions (2–3 h/session and  1 session/week) and 2 individual training (0.5 h/person  and once every 2 weeks). | Social and communication skills measured using by Autism Behavior Checklist (ABC),  Childhood Autism Rating Scale (CARS), and Gesell Developmental Schedule (GDS): | Intervention markedly improve behavioral problems, core symptoms, adaptability, language compeence, and social development capability. |
| Lund, 2013 | Kenya, Rural areas of central Kenya in the Meru South and Nyeri North districts | Uncontrolled before and after | 203 adults with psychosocial impairment | Networking and social support and AT and rehabilitation. Basic needs mental health and development programme (mental health services, community engagement meetings, SHGs, livelihood training, and income-generating activities | Social Relationships-Interpersonal and family relationships and peer and community relationships. | There was an improvement in the social relationships score at 12 months (11.6) and again at 24 months (14.4) |
| Manohar et al., 2019 | India | Randomised controlled trial | Children with autism spectrum disorder in the age of 2-6 years. 50 children, 26 intervention, 24 control | Parent-mediated naturalistic developmental behavioural approach | Parental stress and coping measured by FISC and for autism symptom severity measured by CARS | Significant improvement in perceived  stress and coping at the end of 12 weeks of intervention, and improvement in child joint attention, imitation and social engagement within 12 weeks. |
| McConachie et al., 2010 | Bangladesh | Randomised controlled trial | 85 children with cerebral palsy between the ages of 1.5 and 5 years | Parent training programme and social and communication skill training | Social and communication skills; changes in children’s  adaptive skills, maternal stress and adaptation to the child, satisfaction with  social support, and knowledge of handling a physically disabled child. | Improvement in social and communication skills of children, and mothers’ adaptation did increase. |
| Nair et al., 2014 | India | Pre and post | Parents of children with ASD | Low-intensity, parent-mediated early intervention | Parental stress and coping measured by FISC and for autism symptom severity measured by CARS and receptive expressive emergent language scale | Parent-mediated interventions improved ASD symptoms as well as social and language skills, sensory-motor and adaptive skills |
| Pajareya, 2011 | Thailand | Randomised Controlled Trial | 32 pre-school children with intellectual and learning disorder (ASD) (aged 2–6 years). Treatment group=16 and control group=16 | Informal personal assistance and support. Home-based DIR/Floortime™ intervention – a parent skills intervention; one-day training workshop, plus three-hour DVD lecture and manual. 20 hours training per week for three months. | Emotional (functional emotional assessment and functional emotional development), which includes measures of social skills (e.g. engagement and relating and interaction). | There was an improvement in the emotional scores and emotional development scores of children in the intervention group compared with controls |
| Pop, 2013 | Romania | Randomised controlled trial | 20 children with intellectual/developmental disability (ASD) (4-9 years). Computer-assisted treatment group=6, robot assisted treatment group=7, control group=7 | Social stories were delivered on a computer (SS-PC sessions) and those where the social stories were delivered by the social robot (SS-RAT sessions). Six sessions were given. | Social behaviour and social and communication skills. There was a positive effect in increasing the independency in expressing social abilities of children with ASD | here was a positive effect in increasing the independency in expressing social abilities of children with ASD |
| Rahmani et al., 2015 | Iran | Quasiexperimental | 4 family members of patients with schizophrenia  (37 Experimental and 37 Control) | Group Psychoeducational Programme | Opinion about Mental Illness (OMI) | Improvement in family  attitude toward mental  illness |
| Ravindren et al.,, 2018 | India | Uncontrolled before and after | 14 Male adults with psychosocial impairment. Mean age: 49 | Social and communication skill training was provided for 6 months. | Functional outcomes that included social and communication skills showed improvement in all scales | The median in communication scale changed from 3.12 to 4.63 with an effect size of 0.82 |
| Rami et al., 2018 | Egypt | Randomised controlled trial | 30 adults with psychosocial impairments with their caregivers. Age:18-65 years | Culturally sensitive Behavioral Family Psycho-Educational Program (BFPEP). Intervention component included psychoeducation + communication enhancement training + problem-solving skills training 14 one-hour sessions (weekly in the first 2 months, twice/month in the second 2 months, then every 3 weeks for the last 2 months | I mprovement of clinical variables including social functions. The Social Functioning Questionnaire (SFQ) | A statistically significant difference (p < 0.05) between pre- and post-treatment scores in people with schizophrenia in the case group receiving the BFPEP on the SFQ and all their subscales, indicating better social functioning at post-treatment. |
| Rami et al., 2018 | China | Cluster randomised controlled trial | 326 people with psychosocial impairment. Intervention group 1: family psychoeducation+depot medication. Intervention group 2: depot medication. Control group: Treatment as usual. | Psychoeducational intervention (family education, family workshops, crisis intervention when necessary) with medication; and medication. 9 monthly sessions+ 3 multiple family workshops | Interpersonal and family relationship. Relatives’ beliefs about the illness and towards the person. | The results showed an improvement in the relatives’ caring attitudes towards the person with disability, a gain in knowledge, and an increase in treatment compliance in the psychoeducational family intervention group. |
| Shin et al., 2009 | Vietnam | Randomised controlled trial | 30 pre-school children with intellectual impairment (aged 3–6). Treatment group=16 and control group=14 | Informal personal assistance and training: Individual in-home parent training based on the Portage curriculum training parents to work with their children in the absence of professional resources. One 1-h session per week for 52 wk | There were no significant differences at six months between children in the intervention and control groups in terms of social skills, interpersonal relationships, and play and leisure time | Both treatment and control groups showed gains across time in daily skills difference not significant. d = 0.09 (95% CI 20.63 to 0.80) |
| Shore et al., 2012 | India, Vietnam | Uncontrolled before and after | 206 People with physical impairment. Age range=4-102 | Low-cost wheel chair | Receiving a simple and durable wheelchair improved the reported health, quality of life, and amount of time spent outside the home, interaction and relationships, domestic life, and community, social, and civic engagement following 12 months of use. | Increase in independence associated with the use of wheelchair (independence score changed from 3.9 to 5.0 over 12 months |
| Wang et al., 2008 | China | Randomised controlled trial | 27 families of children with intellectual/developmental disability (ASD) aged <10 years). Treatment group=15 and control group=12 | Parent training intervention | Following the training, parents in the training group, compared with those in the control group, were more sensitive to their children’s interests, responded to their children’s behaviour more appropriately, were more accepting of their children and their behaviour | The analyses indicated that the parents in the training group (Mean = 4.08), compared with those in the control group (Mean = 3.36), scored significantly higher on the responsiveness dimension of MBRS during posttest |
| Yildiz et al., 2004 | Turkey | Controlled before and after | Adult with schizophrenia, 30 participants, 15 in treatment and 15 in control group | psychosocial skills training program | Patients were assessed using the Positive and Negative  Syndrome Scale (PANSS), Quality of Life Scale (QLS), Social Functioning  Scale (SFS), and Global Assessment ofFunction (GAF | There was significant improvement in ‘social functioning’ and ‘quality of  life’ for patients with  schizophrenia. |
| Zuurmond, 2018 | Ghana | Uncontrolled before and after | 75 children with physical impairment (cerebral palsy) and their caregivers. | Participatory parent training programme | Caregivers reported significant improvements in knowledge and confidence in caring for their child. | Caregivers reported significant improvements in knowledge and confidence in caring for their child (p<0.001), in some aspects of child feeding practices (p<0.001) and in their child’s physical and emotional heath (p< 0.001) |
|  |  |  | Age: age 18 months-12 years. | Eleven once-a-month group training sessions each approximately three hours |  |  |

## 4 List of excluded studies

**Excluded on intervention**

1. Carew, M. T., Deluca, M., Groce, N., & Kett, M. (2019). The impact of an inclusive education intervention on teacher preparedness to educate children with disabilities within the Lakes Region of Kenya. *International Journal of Inclusive Education*, *23*(3), 229-244.

2. Cenk, S. C., Muslu, G. K., & Sarlak, D. (2016). The effectiveness of structured, supported education programs for families with intellectually disabled children: The example of Turkey. *Archives of psychiatric nursing*, *30*(6), 704-709.

**Excluded due to studies from high-income countries**

**1.** Feng, H., Lo, Y. Y., Tsai, S., & Cartledge, G. (2008). The effects of theory-of-mind and social skill training on the social competence of a sixth-grade student with autism. *Journal of positive behavior interventions*, *10*(4), 228-242.

2. Kim, J. M., & Mahoney, G. (2004). The effects of mother’s style of interaction on children’s engagement: Implications for using responsive interventions with parents. *Topics in Early Childhood Special Education*, *24*(1), 31-38.

**Excluded due to target population not as per the inclusion criteria**

1. Özcan, C. T., Oflaz, F., Türkbay, T., & Clevenger, S. M. F. (2013). The effectiveness of an interpersonal cognitive problem-solving strategy on behavior and emotional problems in children with attention deficit hyperactivity. *Nöro Psikiyatri Arşivi*, *50*(3), 244.

2. Wolmer, L., Laor, N., Dedeoglu, C., Siev, J., & Yazgan, Y. (2005). Teacher‐mediated intervention after disaster: a controlled three‐year follow‐up of children’s functioning. *Journal of Child Psychology and Psychiatry*, *46*(11), 1161-1168.

**Excluded on outcome (studies did not report social inclusion outcomes)**

**1.** Kumar, C. N., Thirthalli, J., Suresha, K. K., Venkatesh, B. K., Arunachala, U., & Gangadhar, B. N. (2017). Antipsychotic treatment, psychoeducation & regular follow up as a public health strategy for schizophrenia: Results from a prospective study. *The Indian Journal of Medical Research*, *146*(1), 34.

2. Kulhara, P., Chakrabarti, S., Avasthi, A., Sharma, A., & Sharma, S. (2009). Psychoeducational intervention for caregivers of Indian patients with schizophrenia: a randomised‐controlled trial. *Acta Psychiatrica Scandinavica*, *119*(6), 472-483.
